# Classification of early and late stage liver hepatocellular carcinoma patients from their genomics and epigenomics profiles

**DOI:** 10.1371/journal.pone.0221476

**Published:** 2019-09-06

**Authors:** Harpreet Kaur, Sherry Bhalla, Gajendra P. S. Raghava

**Affiliations:** 1 Bioinformatics Centre, CSIR-Institute of Microbial Technology, Chandigarh, India; 2 Department of Computational Biology, Indraprastha Institute of Information Technology, New Delhi, India; 3 Centre for Systems Biology and Bioinformatics, Panjab University, Chandigarh, India; Johns Hopkins University School of Medicine, UNITED STATES

## Abstract

**Background:**

Liver Hepatocellular Carcinoma (LIHC) is one of the major cancers worldwide, responsible for millions of premature deaths every year. Prediction of clinical staging is vital to implement optimal therapeutic strategy and prognostic prediction in cancer patients. However, to date, no method has been developed for predicting the stage of LIHC from the genomic profile of samples.

**Methods:**

The Cancer Genome Atlas (TCGA) dataset of 173 early stage (stage-I), 177 late stage (stage-II, Stage-III and stage-IV) and 50 adjacent normal tissue samples for 60,483 RNA transcripts and 485,577 methylation CpG sites, was extensively analyzed to identify the key transcriptomic expression and methylation-based features using different feature selection techniques. Further, different classification models were developed based on selected key features to categorize different classes of samples implementing different machine learning algorithms.

**Results:**

In the current study, in silico models have been developed for classifying LIHC patients in the early vs. late stage and cancerous vs. normal samples using RNA expression and DNA methylation data. TCGA datasets were extensively analyzed to identify differentially expressed RNA transcripts and methylated CpG sites that can discriminate early vs. late stages and cancer vs. normal samples of LIHC with high precision. Naive Bayes model developed using 51 features that combine 21 CpG methylation sites and 30 RNA transcripts achieved maximum MCC (Matthew’s correlation coefficient) 0.58 with an accuracy of 78.87% on the validation dataset in discrimination of early and late stage. Additionally, the prediction models developed based on 5 RNA transcripts and 5 CpG sites classify LIHC and normal samples with an accuracy of 96–98% and AUC (Area Under the Receiver Operating Characteristic curve) 0.99. Besides, multiclass models also developed for classifying samples in the normal, early and late stage of cancer and achieved an accuracy of 76.54% and AUC of 0.86.

**Conclusion:**

Our study reveals stage prediction of LIHC samples with high accuracy based on the genomics and epigenomics profiling is a challenging task in comparison to the classification of cancerous and normal samples. Comprehensive analysis, differentially expressed RNA transcripts, methylated CpG sites in LIHC samples and prediction models are available from CancerLSP (http://webs.iiitd.edu.in/raghava/cancerlsp/).

## Introduction

Liver Hepatocellular Carcinoma (LIHC) or Hepatocellular Carcinoma (HCC) is the fifth most common cancer and considered as the second major cause of cancer-related mortality with nearly 7,88,000 deaths occurring worldwide in the year 2015 [1]. Further in the United States, there is an estimation of approximately 31,780 deaths and 42,030 new cases in 2018. It is nearly two times more frequent in males than in females. Moreover, a higher number of LIHC cases is reported in Africa and Asia than in Europe [2]. These observations indicate that many factors like viral hepatitis infection (hepatitis B or C) or cirrhosis, smoking, alcohol and lifestyle, *etc*. contribute the pathogenesis of LIHC [3]. Despite improved screening and discoveries, LIHC exhibits rapid clinical course with elevated mortality rate. Patients with LIHC are usually identified at advanced stages due to the lack of pathognomonic symptoms, which consequently limits the potential treatment options and leads to early death [4]. Furthermore, there is a high recurrence rate of 70%, even after curative resection treatment [5]. Therefore, the absolute cure of this disease is quite challenging, indicating an urgent need for the identification of sensitive diagnostic and prognostic markers for LIHC [6,7].

Traditionally in most of the developing countries, alpha-fetoprotein (AFP) is extensively employed as LIHC biomarker. Its level becomes detectable in patients once a tumor is in an advanced stage [8,9]. Besides, AFP-L3, a glycoform of AFP (AFP reacts with Lens culinaris agglutinin) has also been employed as LIHC biomarker due to its higher sensitivity and specificity than alone AFP. The lack of reliability, insufficient sensitivity and specificity are the major limitations associated with these markers [10]. Des-gamma-carboxyprothrombin (DCP) is another vital biomarker. Its levels have shown to be upregulated in advanced stages [11–13].

In recent times, next-generation sequencing technology and bioinformatics analysis emergence have facilitated the identification of tumor diagnostic and prognostic biomarkers candidates of LIHC [14]. Anomalous expression of cancer-associated genes is one of the main causes of tumorigenesis and plays a vital role in hepatocarcinogenesis [15]. Evidently, various reports have shown the elevated expression of *USP22*, *CBX6*, *NRAGE*, *ACTL6*, and *CHMP4B* genes correlate with larger tumor size, advanced tumor stages, poor prognosis and short survival time of patients in LIHC [16–20]. Moreover, the downregulation of *BTG1*, *FOXF2*, and *CYP3A5* genes, have been observed to link with poorly differentiated and aggressive tumors, shorter disease survival rates and shorter recurrence times in LIHC [21–23]. Beside this, recently, long non-coding RNAs have also been found to be aberrantly expressed and implicated in LIHC pathogenesis. For instance, *ZEB1- AS1* and *ANRIL* get upregulated in higher histological grade and stage in LIHC [24–28]. Hence, the potential reversibility of epigenetic abnormalities and restoration of the expression of tumor suppressor genes or other genes by specific inhibitors offer a rational therapeutic approach for LIHC. Although in recent past, numerous tumor drivers for LIHC identified like TERT, CTNNB1, *etc*.; however, most of them have not been translated into efficient modalities [29].

In addition, various studies have shown that the alterations in epigenetics and miRNA pattern lead to progression from precancerous lesions to LIHC [30,31]. The epigenetic modifications including DNA hypermethylation or hypomethylation, dysregulation of histone modification patterns, chromatin remodelling *etc*. are associated with LIHC [32]. The promoter hypermethylation leads to inactivation of various tumor suppressor genes such as *SOCS1*, *hMLH1*, *GSTP1*, *MGMT*, *CDH1*, and *TIMP3*, *etc*. [33].

Detection of cancer at an early stage is vital to reduce the mortality rate by providing appropriate treatment based on the cancer stage. In previous studies, researchers focused mainly on the identification of differentially expressed RNA transcripts or genes in cancerous vs. normal or cancer vs. other liver disease conditions to elucidate marker for LIHC [34–40]. To the best of our knowledge, no method has been developed for predicting the stage of LIHC from the genomic profile of samples. In this study, a systematic attempt has been made to a develop model for discriminating early and late stage of LIHC samples. First, we identified CpG sites that are differentially methylated in the early vs. late stage of LIHC and cancerous vs. normal. Second, we identified aberrantly expressed RNA transcripts that can differentiate early stage from the late stage of LIHC and cancerous from non-tumorous samples of LIHC. Ultimately, models were developed based on different machine learning techniques for predicting the stage of LIHC samples using the above derived genomic and epigenomic features. Using diverse feature spaces, we were able to establish models discriminating early and late stage of LIHC cancer. In addition, we tried to develop models to distinguish normal and LIHC tissue samples. Our models successfully predict LIHC samples with high accuracy. It indicates that it is easy to predict LIHC samples, but it is challenging to classify them in early and late stage. We also attempted to develop multiclass prediction models to categorize samples in three classes: i) normal or control samples, ii) LIHC early stage and iii) LIHC late stage tissue samples.

## Methods

### Data description

#### Main dataset of early & late stage samples

We extracted the expression and methylation profiles of Liver Hepatocellular Carcinoma (LIHC) samples from GDC Data Portal (https://portal.gdc.cancer.gov/). In addition, manifest, metadata, clinical data, biospecimen files were downloaded to extract clinical information using Biospecimen Core Resource (BCR) IDs of patients. Finally, we obtained 173 stage-I, 87 stage-II, 85 stage-III and 5 stage- IV stage samples. Clinical characteristics of these patients displayed in Figure A in S2 File. As the number of stage-IV samples in the dataset is small, we have considered stage-II, stage-III and stage-IV samples as late stage samples, while stage-I samples as early stage samples as stage I samples are of localized cancer which shows no sign of metastasis. We also downloaded the methylation profiles acquired using the Illumina Human- Methylation450K DNA Analysis BeadChip assay, based on genotyping of bisulfite-converted genomic DNA at individual CpG-sites. This data provided Beta values, a quantitative measure of DNA methylation [41]. Besides, for each subject, RNA expression in terms of FPKM values for 60,483 RNA transcripts was reported. In this study, we have used FPKM values of RNA transcripts as quantification values.

#### Pre-processing of data

**Methylation Data**. There are a total of 485,577 Methylation CpG sites (Probe IDs associated with CpG sites) for each tissue sample. The methylation score for every CpG site was defined in terms of beta value. Approximately 23–25% CpG sites excluded from the study for which beta value is missing among any of samples using an in-house bash script. Hence CpG sites number reduced to 374,292 for staging analysis and 369,221 for Cancer v/s Normal analysis.

**Normalization of RNA Expression**. The expression values of RNA transcripts from the GDC portal were obtained in terms of FPKM (fragments per kilobase of transcript per million mapped reads). There is a wide range of variation in FPKM values, thus we transformed values using log2 after addition of 1.0 as a constant number to each of FPKM value. Further, features with low variance were removed and data was z-score normalized using the *caret* package in R [42]. The following equations were used for computing the transformation and normalization:

$$x={Log}_{2}(FPKM+1)$$ (1)

$$Z_{\_score}=\frac{x-\mu }{sd}$$ (2)

Where *Z_*_*score*_ is the normalized score, *x* is the log-transformed expression, μ is the mean of expression and *sd* is the standard deviation of expression. The validation data was Z score normalized using the mean and variance of the training data.

As methylation beta values vary between 0 to 1; thus, normalization is not performed on methylation data. Further to build a hybrid model based on methylation of CpG sites and RNA expression of transcripts, we have transformed FPKM value to 0 to 1 using the Min-Max normalization method of *caret* package in R [42].

### Identification of differentially methylated CpG sites and expressed RNA transcripts

To identify differentially CpG methylated sites; first, we computed the mean methylation score for each CpG site in early and late stage samples. Secondly, we calculated whether the difference in the mean of methylation score in early and late stage samples was statistically significant or not using the *t*-*test*. Here, the Welch t-test or Yuen-Welch test was implemented using in-house R and bash scripts. In literature, Welch’s t-test considered robust for skewed distributions and large sample sizes [43,44]. Similarly, differentially expressed RNA transcripts in early and late stage samples from a total of 60,483 RNA transcripts were identified.

### Feature selection techniques

One of the challenges in developing a prediction model is to identify essential features from the large dimension of features. In this study, we used a number of techniques for feature selection. First, we used Area under Receiver operating characteristic (AUC) based feature selection technique in which we developed single feature-based models for discriminating early and late stage samples. The single feature-based models (threshold based models) were developed as wherein the features with a score above than a threshold are assigned to the early stage if it is found to be upregulated in the early stage [45]. We compute the performance of each model based on a given feature and identify features having the highest performance in term of AUC. Second, we perform feature selection using two different algorithms like attribute evaluator named, ‘SymmetricalUncertAttributeSetEval’ with search method of ‘FCBFSearch’ of WEKA software package and sklearn.feature_selection F-ANOVA method from Scikit package. The FCBF (Fast Correlation-Based Feature) algorithm employed correlation to identify relevant features in high-dimensional datasets in small feature space [46].

### Implementation of machine learning techniques

Primarily, we have developed the Support Vector Machine (SVM) based prediction models using the package SVM^*light*^ [47] and WEKA [46]. In the present study, the RBF (radial basis function) kernel was employed to optimize various parameters to get the best performance on the training dataset. Furthermore, some of the commonly used classifiers were employed for developing the prediction models. These classifiers include Random forests, SMO, Naïve Bayes, and J48 were implemented exploiting WEKA software.

### Performance evaluation of models

In this study, we used both cross-validation and independent validation technique to evaluate the performance of models. As our dataset contains a reasonable number of samples *i*.*e*. a total of 350 (for stage analysis) and 425 (for cancer v/s normal analysis); therefore, it is crucial to develop and train the model with the appropriate number of samples to avoid parameter estimation variance. In the past, various studies employed the 80:20 ratio for the partitioning of a dataset into training and validation dataset [48–52]. Hence, we applied this standard protocol and divided our dataset into two datasets in the ratio of 80:20; where 80% data is used for training called training dataset and the remaining 20% data is used for validation called validation dataset or independent dataset. The training dataset used for building and evaluating our models using 10-fold cross-validation technique, where nine folds are used for training and remaining one fold for testing and process is repeated ten times so that each fold is used once for testing. The performance of models on the testing dataset is called internal validation. In internal validation, we optimize parameters to achieve the best performance on the test dataset. Though models are trained and tested on separate sets still over optimization of models cannot be ruled out. To avoid over optimization, we evaluate the performance of our final model from internal validation (10-fold cross-validation) on an independent dataset which is not used for training or testing our final model.

To measure the performance of models, we used standard parameters, commonly used to measure the performance of classification models. Both threshold-dependent and threshold-independent parameters were employed to measure performance. In the case of threshold-dependent parameters, we computed sensitivity, specificity, accuracy and Matthew’s correlation coefficient (MCC) using the following equations.

$$Sensitivity(Sens)=\frac{TP}{TP+FN}*100$$ (3)

$$Specificty(Spec)=\frac{TN}{TN+FP}*100$$ (4)

$$Accuracy(Acc)=\frac{TP+TN}{TP+FP+TN+FN}*100$$ (5)

$$MCC=\frac{\left(TP*TN)-(FP*FN\right)}{\sqrt{\left(TP+FP\right)}(TP+FN)(TN+FP)(TN+FN)}$$ (6)

Where, FP, FN, TP, and TN are false positive, false negative true positive and true negative predictions, respectively.

While, for threshold-independent measures, we used standard parameter Area Under the Receiver Operating Characteristic curve (AUROC) or commonly known as Area Under (AUC) or Receiver Operating Characteristic curve (ROC). The AUC curve is generated by plotting sensitivity or true positive rate against the false positive rate (1-specificity) at various thresholds. Finally, the area under the ROC curve calculated to compute a single parameter called AUC. AUC with CI (Confidence Interval) computed using the *pROC* package in R [53].

### Functional annotation or enrichment analysis of genes

In order to discern the biological relevance of the signature genes or the genes associated with signature CpG sites, enrichment analysis performed using Enrichr [54]. Enrichr applies Fisher exact test to identify enrichment score. It also provides Z-score which is derived by applying correction on a Fisher Exact test.

## Results

### Models for classification of early stage and late stage of LIHC samples

**Fig 1:**
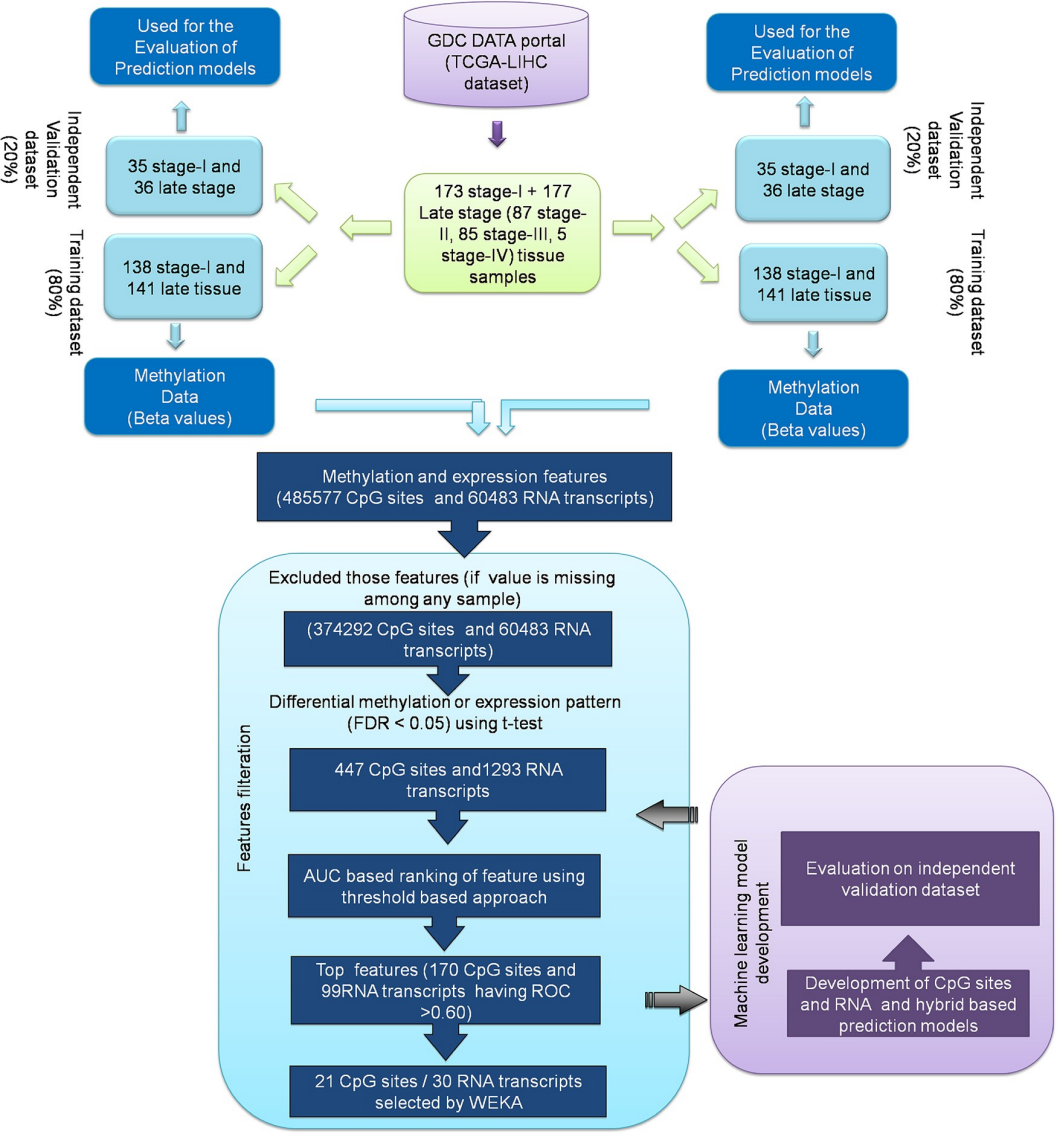
The workflow representing the analysis of methylation and expression profile of LIHC early and late stage samples.

Our primary objective is to identify potential markers, *i*.*e*. CpG sites and RNA transcripts that can classify early stage and late stage tissue samples. Subsequently, in-silico predictive models were developed based on these signature markers using various machine learning algorithms. Potential markers and prediction models based on them are explained in the following sections. The overall workflow represented in Fig 1.

#### Single feature-based stage classification model using CpG sites

Here, we used a single feature-based classification technique to classify early and late stage samples. In this study, we first identified 447 CpG sites (from 3,74,292 sites) that are significantly differentially methylated in an early and late stage of the sample using t-test (FDR (False Discovery Rate) < 0.05). These differentially methylated sites are further segregated in two classes; i) 199 hypermethylated CpG sites (high level of methylation in early stage) and ii) 248 hypomethylated CpG site (low-level methylation in early stage). Subsequently, each differentially methylated CpG sites is used to develop a simple threshold-based model for classifying early and late stage samples. In the threshold-based model, a sample is categorized in the early stage if the methylation level of CpG site (in case site is hypermethylated) is higher than a threshold value otherwise in late stage. In these models, the threshold is varied incrementally from minimum to maximum beta-value. In the final step, that threshold is selected which leads to maximum AUC between early and late samples. Subsequently, all the 447 CpG sites are ranked according to AUC to assess the ability of a CpG site to categorize early and late stage tissue samples (Table A in S1 File). As shown in Table A in S1 File, there are 170 differentially methylated CpG sites (named as LS-CPG-AUC) that can differentiate two types of samples with high precision (AUC ≥ 0.6). LS-CPG-AUC includes 105 hypermethylated and 65 hypomethylated CpG sites. Hypermethylated CpG sites in the early stage such as cg20457523, cg18563987, cg18578954 can distinguish early and late stage samples with AUC 0.66, 0.65 and 0.65 at threshold 0.83, 0.70 and 0.44 respectively; while the hypomethylated sites in the early stage include cg16876964 and cg00590251 have AUC 0.64 and 0.63 at thresholds 0.90 and 0.75. Methylation pattern of the top 20 CpG from 447 CpG sites and their chromosome location and associated genes are represented in Figure B in S2 File.

LS-CPG-AUC signature is significantly enriched (adjusted *p*-value <0.05) in various immune system associated pathways and cancer-associated cell signaling pathways present in BioCarta. For instance, 4 out of 55 genes of T Cell Receptor Signaling Pathway and 5 out of 33 genes of Integrin Signaling Pathway_Homosapiens, are enriched in LS-CPG-AUC. Furthermore, *ITGB1*, *SHC1*, and *PTK2* are associated with PTEN dependent cell cycle arrest and apoptosis, VEGF, Hypoxia, and Angiogenesis Pathway. This signature is also enriched in actin filament binding (GO:0051015).

#### Multiple features-based stage classification model using CpG sites

One of the challenges in classification models is to improve the performance of models. Though, we got a single CpG site like cg20457523, which can classify samples with AUC 0.66. To utilize information from multiple features, we developed models using machine learning techniques. Firstly, models developed using all 170 features of LS-CPG-AUC. Most of the models attain reasonable high performance on training dataset with maximum AUC 0.79 for SVM model, but reduced AUC, *i*.*e*. 0.66 on validation dataset as shown in Table B in S1 File.

**Fig 2:**
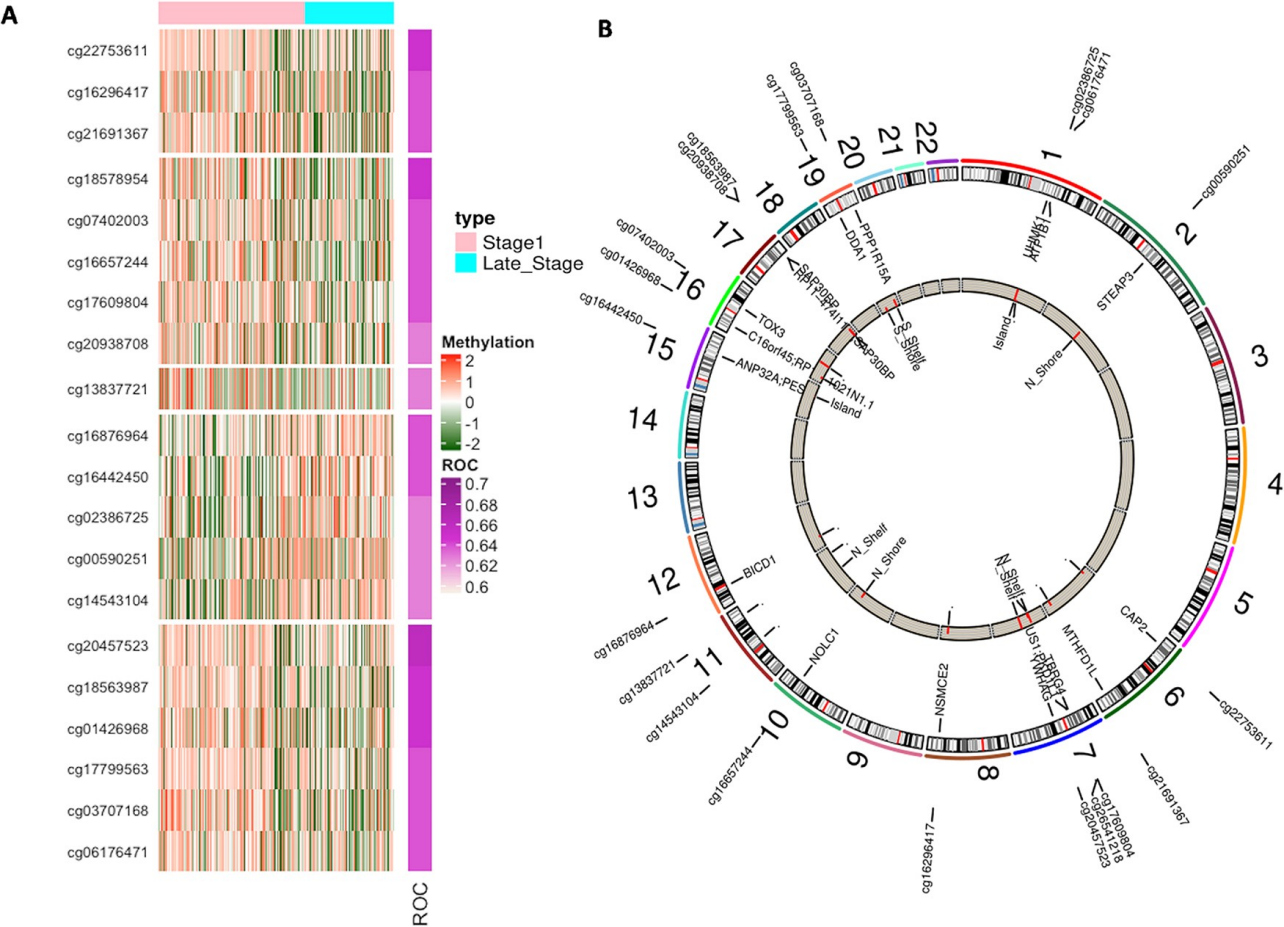
A) Heatmap displaying the differential methylation pattern (with FDR < 0.05) and B) Circos plot representing the chromosome location of top 21 CpG sites (LS-CPG-WEKA) in early versus late stage of LIHC.

Therefore, we selected 21 features using WEKA from 447 CpG differentially methylated sites (with FDR < 0.05) for early-stage v/s late-stage. These 21 selected features (CpG sites) named as LS-CPG-WEKA were used to develop models for classification implementing different machine learning techniques (Table 1). The Random Forest model achieved the highest performance with an accuracy of 75.27% and AUC 0.8 on the training dataset and with higher accuracy of 77.46 and AUC 0.79 on the validation dataset as shown in Table 1. The SVM, SMO and Naïve Bayes models performed almost in a similar range for training dataset, but there is a slight decrease in performance on validation datasets. Among 21 CpG sites (selected features), 9 and 12 were hypomethylated and hypermethylated CpG sites respectively. Heatmap displays the methylation pattern of these CpG sites and Circos plot represents their chromosome location, associated genes and feature type, are shown in Fig 2.

**Table 1:** The performance of different machine learning techniques-based stage classification models developed using 21 methylation CpG sites selected by WEKA (LS-CPG-WEKA).

Machine Learning Techniques	Dataset	Performance Measures				
		Sensitivity (%)	Specificity (%)	Accuracy (%)	MCC	AUC with 95%CI
SVM	Training	80.43	71.63	75.99	0.52	0.81 (0.69–0.89)
	Validation	71.43	75	73.24	0.46	0.78 (0.67–0.89)
Random Forest	Training	77.54	73.05	75.27	0.51	0.8 (0.69–0.89)
	Validation	71.43	83.33	77.46	0.55	0.79 (0.68–0.87)
Naïve Bayes	Training	87.68	62.41	74.91	0.52	0.82 (0.73–0.89)
	Validation	80	63.89	71.83	0.44	0.82 (0.76–0.84)
SMO	Training	82.61	69.5	75.99	0.53	0.76 (0.68–0.83)
	Validation	74.29	75	74.65	0.49	0.75 (0.69–0.80)
J48	Training	62.32	75.18	68.82	0.38	0.67 (0.61–0.72)
	Validation	45.71	61.11	53.52	0.07	0.57 (0.52–0.63)

Interestingly 9 hypermethylated CpG sites of LS-CPG-WEKA signature in early stage are associated with 12 genes (*EEF2*, *NOLC1*, *PPP1R15A*, *DNAJC14*, *DNAJB6*, *XYLT1*, etc.) that are significantly enriched (adjusted p-value <0.05) in various GO biological processes such as two genes are involved in positive regulation of translation (GO:0045727) and rescue of stalled ribosome (GO:0072344). While, hypomethylated CpG sites are associated with 14 genes (*SMAD7*, *PCNX*, *RGL2*, *POLR1D*, *ZNF554*, *ZNF200*, *etc*.) and these genes are significantly enriched in different BioCarta pathways. For instance, *SMAD7*, a component of catenin complex (GO:0016342), is involved in TGF beta signaling pathway and has been shown to inhibit TGF-beta (Transforming growth factor) and active in signaling by associating with their receptors. *ZNF554*, *PLOR1D*, and *PSPC1* are involved in the regulation of transcriptional activity.

#### Stage classification models using the expression of RNA transcripts

In the above section, we studied the methylation level of CpG sites in early and late stage samples as well as the classification models developed using them as features. Similarly, in this section, we analyzed the expression of RNA transcripts in both types of samples and afterwards developed stage classification models. Firstly, the number of the RNA transcripts was reduced to 103 (LS-RNA-AUC) after applying stringent criteria (FDR adjusted p-value < 0.01) from 60,483. In LS-RNA-AUC signature, 39 transcripts were over-expressed, and 64 transcripts were underexpressed in early stage LIHC samples. Secondly, we developed single feature-based models using each transcript, similar to single features-based models for CpG sites. Finally, RNA transcripts ranked based on AUC to distinguish early stage from late stage tissue samples. The 99 RNA transcripts having AUC greater or equal to 0.6, are shown in Table (Table C in S1 File) with their mean expression values. Furthermore, *NCAPH*, *CYP4A22*, *HSD17B6*, *GLYATL1*, and *FTCD* are top 5 RNA transcripts with AUC > = 0.65. Among them, *NCAPH* is the top performer with AUC 0.66 at threshold 1.45. The expression pattern of the top 20 genes (LS-RNA-AUC) in early stage samples in comparison to late stage tissue samples displayed in Figure (Figure C in S2 File). Their biological importance and implication in liver cancer or any other malignancy displayed in Table D in S1 File.

Gene enrichment analysis of LS-RNA-AUC which includes 61 downregulated and 39 upregulated transcripts in the early stage of LIHC using Enrichr displayed in Table 2. The enrichment analysis of these transcripts indicates that transcripts which are upregulated in late stage are mostly enriched in metabolic processes related pathways like glycolysis, pentose phosphate pathways, *etc*.; whereas, transcripts which are downregulated in late stage) are associated with pathways that involved in the normal functioning of liver like drug metabolism, xenobiotics metabolism. Further, transcripts which are upregulated in late stage are enriched in cell cycle GO terms that positively regulate cell growth, while transcripts which are downregulated in late stage are enriched in GO terms associated with the general functioning of liver like lipid metabolism. It suggests there is an acceleration in growth-related and metabolic processes with the tumor progression (from early to late stage) since the enhanced nutrients requirement by tumor cells as the tumor grow [55]. This indicates, as the tumor progresses from early to late stage, there might be a disturbance in the normal functioning of liver-associated pathways and processes. In addition, downregulated transcripts enriched in MSigDB oncogenic signatures *i*.*e*. RPS14_DN. V1_DN and CSR_LATE_UP.V1_UP, while upregulated transcripts enriched in PKCA_DN.V1_UP.

**Table 2:** Gene enrichment analysis of LS-RNA-AUC signature using Enrichr. This signature contains 61 downregulated and 39 upregulated RNA transcripts in early stage in comparison to late stage of LIHC).

Biological importance	61 RNA transcripts (genes) downregulated in early stage		39 RNA transcripts (genes) upregulated in early stage	
	Name	Adjusted p-value	Name	Adjusted p-value
**Panther Pathway**	Glycolysis_Homo sapiens_P00024	0.0001441	Blood coagulation_Homo sapiens_P00011	0.00000434
	Fructose galactose metabolism_Homo sapiens_P02744	0.001562	Pyrimidine Metabolism_Homo sapiens_P02771	0.0004127
	Ubiquitin proteasome pathway_Homo sapiens_P00060	0.01952		
	Pentose phosphate pathway_Homo sapiens_P02762	0.04431		
**KEGG Pathway**	Glycolysis / Gluconeogenesis_Homo sapiens_hsa00010	0.05063	Metabolic pathways_Homo sapiens_hsa01100	1.74E-09
			Retinol metabolism_Homo sapiens_hsa00830	6.70E-08
			Drug metabolism—other enzymes_Homo sapiens_hsa00983	0.001571
			Arachidonic acid metabolism_Homo sapiens_hsa00590	0.002873
			Drug metabolism—cytochrome P450_Homo sapiens_hsa00982	0.003016
			Metabolism of xenobiotics by cytochrome P450_Homo sapiens_hsa00980	0.003016
			Complement and coagulation cascades_Homo sapiens_hsa04610	0.003016
			Peroxisome_Homo sapiens_hsa04146	0.003016
			Pantothenate and CoA biosynthesis_Homo sapiens_hsa00770	0.003016
			Histidine metabolism_Homo sapiens_hsa00340	0.004777
**GO Cellular Component**	anterior cell cortex (GO:0061802)	0.0002658	smooth endoplasmic reticulum lumen (GO:0048238)	0.004391
	equatorial cell cortex (GO:1990753)	0.0002658	endoplasmic reticulum lumen (GO:0005788)	0.004391
	posterior cell cortex (GO:0061803)	0.0002658	cortical endoplasmic reticulum lumen (GO:0099021)	0.004391
	cell cortex region (GO:0099738)	0.0002658	perinuclear endoplasmic reticulum lumen (GO:0099020)	0.004391
	mitotic spindle midzone (GO:1990023)	0.00211	sarcoplasmic reticulum lumen (GO:0033018)	0.004391
	meiotic spindle midzone (GO:1990385)	0.00211	rough endoplasmic reticulum lumen (GO:0048237)	0.004391
	condensed nuclear chromosome outer kinetochore (GO:0000942)	0.008363	Golgi lumen (GO:0005796)	0.01546
	mitotic spindle pole (GO:0097431)	0.012	trans-Golgi network transport vesicle lumen (GO:0098564)	0.01792
	ficolin-1-rich granule lumen (GO:1904813)	0.01519	Golgi stack lumen (GO:0034469)	0.02105
	spindle microtubule (GO:0005876)	0.0246	peroxisomal matrix (GO:0005782)	0.02848
**GO Biological Process**	positive regulation of mitotic metaphase/anaphase transition (GO:0045842)	0.0000599	exogenous drug catabolic process (GO:0042738)	0.0003105
	plant-type primary cell wall biogenesis (GO:0009833)	0.0005205	exogenous antibiotic catabolic process (GO:0042740)	0.0003105
	cellular bud neck septin ring organization (GO:0032186)	0.0005205	peptidyl-glutamic acid carboxylation (GO:0017187)	0.0003105
	cellular bud site selection (GO:0000282)	0.0005205	epoxygenase P450 pathway (GO:0019373)	0.0003105
	protein localization to mitotic actomyosin contractile ring (GO:1904498)	0.0005205	signal peptide processing (GO:0006465)	0.002393
	mitotic cytokinesis (GO:0000281)	0.0005205	negative regulation of platelet activation (GO:0010544)	0.001191
	mitotic cytokinetic process (GO:1902410)	0.0005205	fibrinolysis (GO:0042730)	0.001191
	regulation of mitotic spindle checkpoint (GO:1903504)	0.0005315	phytosteroid metabolic process (GO:0016128)	0.001191
	negative regulation of mitotic metaphase/ anaphase transition (GO:0045841)	0.0005315	C21-steroid hormone metabolic process (GO:0008207)	0.001214
	regulation of mitotic metaphase/anaphase transition (GO:0030071)	0.001438	(25S)-Delta(4)-dafachronate metabolic process (GO:1902057)	0.001214
**GO Molecular Function**	Rho GDP-dissociation inhibitor activity (GO:0005094)	0.04347	arachidonic acid epoxygenase activity (GO:0008392)	0.0000015
	Rab GDP-dissociation inhibitor activity (GO:0005093)	0.04347	arachidonic acid 11,12-epoxygenase activity (GO:0008405)	0.0000015
			arachidonic acid 14,15-epoxygenase activity (GO:0008404)	0.0000015
			sodium-independent organic anion transmembrane transporter activity (GO:0015347)	0.001676
			heme binding (GO:0020037)	0.005818
			bile acid transmembrane transporter activity (GO:0015125)	0.009954
			metal ion binding (GO:0046872)	0.01658
			alkali metal ion binding (GO:0031420)	0.01658
			lead ion binding (GO:0032791)	0.01658
			transition metal ion binding (GO:0046914)	0.01658

#### Multiple features-based stage classification model using RNA transcripts

**Fig 3:**
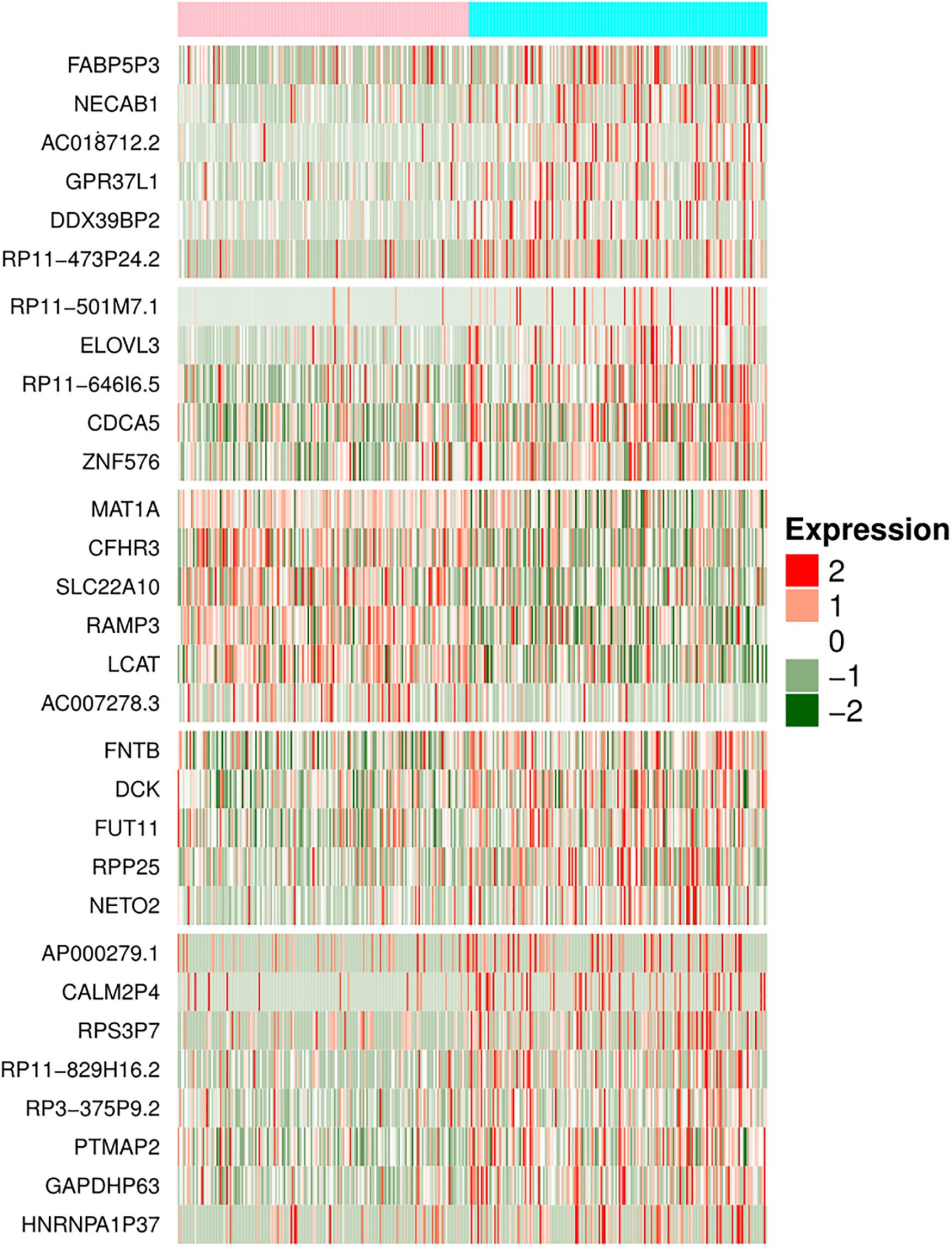
The differential expression pattern of 30 RNA transcripts (LS-RNA-WEKA) in early stage versus late stage tissue samples (With FDR <0.01).

In addition to single feature-based models, prediction models generated using the expression of multiple RNA transcripts implementing different machine learning techniques. First, 30 RNA transcripts (named as LS-RNA-WEKA) selected from 1,293 RNA transcript using WEKA, subsequently prediction models developed for stage classification. As shown in Table 3, the Naive Bayes based model achieved maximum accuracy of 74.55% with AUC 0.79 on training dataset and accuracy of 76.06% with AUC 0.79 on validation dataset respectively (Table 3). Among 30-signature RNA transcripts (LS-RNA-WEKA), 24 RNA transcripts are underexpressed, while six are observed as overexpressed in early stage (Table E in S1 File). Further among 30 transcripts,15 are protein-coding genes which include *CSNK1D*, *DCK*, *ZNF576*, *RPP25*, *SLC22A10*, *FNTB*, *LCAT*, *MAT1A* and *CDCA5*, *etc*., 9 processed pseudogenes include GAPDHP63, RP11-829H16.2, AC018712.2 *etc*., 2 unprocessed pseudogenes, 1 each of lincRNA, miRNA, sense_intronic transcript. Fig 3 represents the expression pattern of these RNA transcripts among early and late stage tissue samples. In addition, we also developed a prediction model using 100 features selected by F-ANOVA method. The performance (accuracy 70.42, AUC 0.74 on independent validation dataset) of the model has decreased with increasing the number of features as shown in Table F in S1 File.

**Table 3:** The performance of stage classification models developed using 30 RNA transcripts selected using WEKA from 103 RNA transcripts (LS-RNA-WEKA).

Machine Learning Techniques	Dataset	Performance Measures				
		Sensitivity (%)	Specificity (%)	Accuracy (%)	MCC	AUC with 95% CI
SVM	Training	80.43	73.05	76.7	0.54	0.8 (0.69–0.89)
	Validation	68.57	75	71.83	0.44	0.77 (0.65–0.89)
Random Forest	Training	81.88	74.47	78.14	0.56	0.84 (0.79–0.88)
	Validation	65.71	58.33	61.97	0.24	0.67 (0.56–0.73)
Naïve Bayes	Training	84.78	64.54	74.55	0.5	0.79 (0.68–0.89)
	Validation	80	72.22	76.06	0.52	0.79 (0.69–0.89)
SMO	Training	75.36	76.6	75.99	0.52	0.76 (0.65–0.88)
	Validation	62.86	77.78	70.42	0.41	0.7 (0.60–0.81)
J48	Training	68.12	72.34	70.25	0.4	0.7 (0.64–0.75)
	Validation	57.14	66.67	61.97	0.24	0.63 (0.53–0.73)

It has been observed that most of the overexpressed genes such as *CDCA5*, *CSNK1D*, *DCK*, *ZNF576* and *RPP25*, *etc*. in the late stage are involved in cell growth promotion processes associated GO terms. While underexpressed genes such as *LCAT*, *CFHR3*, *RAMP3*, *MAT1A*, *etc*. associated with the normal functioning of the liver and immune functions related GO terms as shown in Table D in S1 File. It also represents their implication previously in the liver cancer or any other malignancies Table D in S1 File.

#### Stage classification models using hybrid features

With the aim to build the models with improved performance, prediction models were developed using 51 features that combine 21 CpG sites and 30 RNA transcripts selected by WEKA (named as LS-CpG-RNA-hybrid) to segregate early and late stage tissue samples. The Naïve Bayes based model achieved the highest accuracy of 78.14% with AUC 0.81 on the training dataset and the accuracy of 78.87% and 0.82 on the independent dataset (Table 4). Additionally, we also developed models using 10-fold cross-validation on the full dataset (combining training and validation datasets). The performance of these models is almost in a similar range of models developed using training and independent validation datasets as shown in Table G in S1 File. Furthermore, models built using selected 38 features (15CpG sites and 23RNA transcripts) obtained from 1740 features (significantly differentially expressed 1293 RNA transcripts and significantly differentially methylated 447CpG sites) as shown in Table H in S1 File. In summary, Naïve Bayes based model developed using 51 hybrid features (21 CpG sites and 30 RNA transcripts) is the best performer for classification of early and late stage tissue samples.

**Table 4:** The performance of stage classification hybrid models developed using 51 features (LS-CpG-RNA-hybrid) that comprise 21 CpG sites and 30 RNA transcripts.

Machine Learning Techniques	Dataset	Performance Measures				
		Sensitivity (%)	Specificity (%)	Accuracy (%)	MCC	AUC with 95% CI
SVM	Training	81.16	73.76	77.42	0.55	0.82 (0.73–0.92)
	Validation	80	75	77.46	0.55	0.8 (0.68–0.91)
Random Forest	Training	79.71	75.89	77.78	0.56	0.85(0.77–0.92)
	Validation	71.43	72.22	71.83	0.44	0.79 (0.74–0.83)
Naïve Bayes	Training	89.13	67.38	78.14	0.58	0.81(0.73–0.88)
	Validation	85.71	72.22	78.87	0.58	0.82 (0.74–0.89)
SMO	Training	83.33	73.05	78.14	0.57	0.78 (0.73–0.83)
	Validation	80	72.22	76.06	0.52	0.76 (0.7–0.81)
J48	Training	70.29	68.09	69.18	0.38	0.69 (0.59–0.81)
	Validation	65.71	75	70.42	0.41	0.75(0.65–0.86)

### Models for classification of LIHC and normal samples

#### Models for classification of LIHC and normal samples using CpG sites

In the current study, we also generated prediction models for discriminating LIHC and normal tissue samples using genomic and epigenetic profiles. These models were developed on 424 tissue samples (50 normal and 374 LIHC), where training dataset consists of 339 samples (40 normal and 299 LIHC), and an independent dataset consists of 85 samples (10 normal and 75 LIHC). 1,386 differentially methylated CpG sites identified in LIHC and normal tissue samples (minimum mean difference of methylation beta values is 0.4) with Bonferroni *p*-value < 0.0001. These 1,386 sites contain 125 hypermethylated and 1,261 hypomethylated in cancer samples (data not shown). Further, single feature-based models developed using these CpG sites and ranked them based on the performance of models. Top 496 CpG sites named as LCN-CpG- AUC (68 hypermethylated and 428 hypomethylated CpG sites in LIHC) with AUC equal or greater than 0.9 enlisted in Table I in S1 File. We have obtained 14 CpG sites that can discriminate LIHC and normal samples with high precision (ROC > = 0.95). Single feature-based models developed using CpG site cg07274716, cg24035245 and cg20172627 were able to classify normal and LIHC tissue with AUC 0.97, 0.96 and 0.95 at threshold 0.35, 0.44, 0.32 and 0.12 respectively. The methylation pattern of the top 10 CpG sites is depicted by Heatmap (Figure D in S2 File) and chromosome locations and associated genes of the CpG sites represented in Circos plot (Figure E in S2 File).

**Fig 4:**
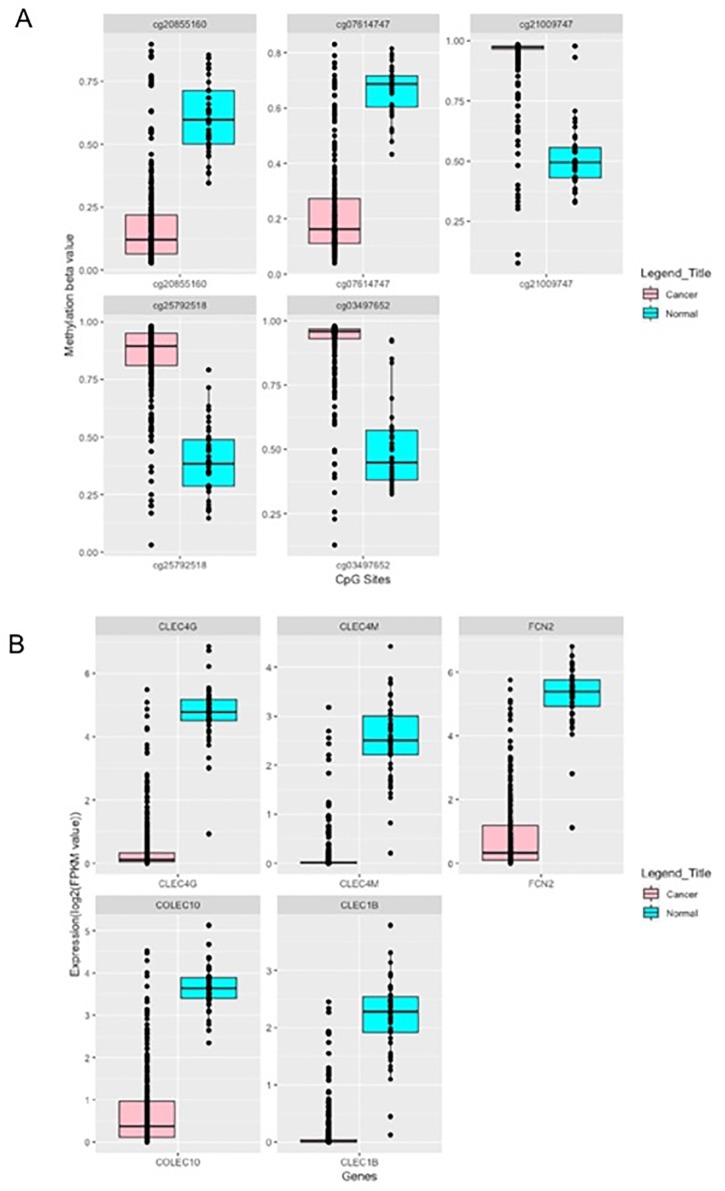
(A) The differential methylation pattern of LCN-5CpG, and (B) the differential expression pattern of (LCN-5RNA) in cancer vs. normal (adjacent non-tumor) tissue samples (Bonferroni adjusted p-value <0.001).

In addition to the single feature-based models, an attempt has been made to develop models using multiple features to categorize LIHC and normal samples. The SVM based model developed using 104 CpG sites (selected using WEKA from 496 CpG sites) achieves maximum accuracy of 98–99% and AUC 0.99 on the independent and training dataset. Feature dimension further reduced and developed models using 100, 50, 25, 20, 10 and 5 features or CpG sites (selected using F-ANOVA), (data not shown). The models based on even small number of features (*i*.*e*. 5 CpG sites named as LCN-5-CpG) got reasonably high performance (ROC ~0.99). Models based on Random Forest, Naïve Bayes, SMO algorithms performed reasonably good with accuracy 95%–97%, and AUC of 0.94–0.97 on the training dataset and accuracy 96–97% and AUC of 0.94–99 on an independent validation dataset (Table 5). This observation shows that LIHC samples can be discriminated with high precision, even using the small number of CpG methylation-based features. Among LCN-5-CpG signature, cg20855160, cg7614747 get hypomethylated; while cg21009747, cg25792518 and cg03497652 get hypermethylated in cancer in comparison to normal samples (Fig 4A).

**Table 5:** The performance models developed for discriminating LIHC and normal samples, using 5 methylation CpG sites.

Machine Learning Techniques	Dataset	Performance Measures				
		Sensitivity (%)	Specificity (%)	Accuracy (%)	MCC	AUC with 95%CI
SVM	Training	99.33	90	98.23	0.91	0.99 (0.97–1)
	Validation	98.67	90	97.65	0.89	0.99 (0.98–0.99)
Random Forest	Training	99	87.5	97.64	0.88	0.97 (0.94–1)
	Validation	98.67	90	97.65	0.89	0.99 (0.97–1)
Naïve Bayes	Training	96.66	90	95.87	0.82	0.94(0.84–1)
	Validation	97.33	90	96.47	0.84	0.95 (0.86–1)
SMO	Training	99	90	97.94	0.9	0.94 (0.88–0.98)
	Validation	98.67	90	97.65	0.89	0.94 (0.84–1)
J48	Training	97.66	80	95.58	0.79	0.86(0.79–0.93)
	Validation	98.67	80	96.47	0.82	0.89 (0.76–1)

To detect the biological relevance of the genes associated with these signature CpG sites (LCN-5-CpG), enrichment analysis performed using Enrichr. This analysis indicates *MYH9* is significantly involved in molecular functions such as actin-dependent ATPase activity (GO:0030898), microfilament motor activity (GO:0060002, GO:0060001). It is also involved in GO biological processes such as positive regulation of protein processing in a phagocytic vesicle (GO:1903923), PMA-inducible membrane protein ectodomain proteolysis (GO:0051088), malonyl-CoA metabolic process (GO:2001293). *ACSF2* is involved in the acyl-CoA metabolic process (GO:0006637), malonyl-CoA metabolic process (GO:2001293), *etc*. Furthermore, the enrichment analysis signifies the involvement of *MYH9* in Nicotinic acetylcholine receptor signaling pathway, Cytoskeletal regulation by Rho GTPase, Inflammation mediated by chemokine and cytokine signaling pathway_Homo sapiens_P00031 PANTHER pathways. In addition, Enrichr suggest the enrichment of *ACSF2* in LEF1_UP.V1_DN, while *CHAD* involved in AKT_UP_MTOR_DN.V1_DN, and AKT_UP.V1_DN MSigDB Oncogenic Signatures set (*p*-value <0.05).

#### Models for classification of LIHC and normal samples using RNA transcripts

Here, 147 significantly differentially expressed RNA transcripts (with Bonferroni p-value < 0.001) identified in LIHC and normal samples (Table J in S1 File). The performance of models developed using top 53 RNA transcripts (ROC equal or greater than 0.9) is shown in Table K in S1 File. Among 53 transcripts, 50 are protein coding, one each of Linc-RNA, snoRNA and processed transcripts. Further, among them, 18 and 35 observed to be overexpressed and underexpressed respectively in LIHC in comparison to normal tissue samples. Moreover, *CLEC4G*, *CLEC4M*, *FCN2*, *COLEC10*, *CLEC1B*, and *PLVAP* can classify LIHC and normal tissue samples with AUC > = 0.96. *CLEC4G* which is an underexpressed gene in cancer is the chief performer with AUC ~0.99 at threshold 2.9; whereas *PLVAP* is overexpressed gene in cancer samples, it can distinguish samples with AUC 0.97 at threshold 3.8. Expression pattern of top 10 RNA transcripts is displayed in the heatmap (Figure D in S2 File).

Gene enrichment analysis of LCN-RNA-AUC is represented in Figure F (Figure F in S2 File). The functional enrichment of these genes suggested their involvement in various growth progression processes. As these genes upregulated in LIHC; this might lay insight toward their contribution in the diseased state. Table L-A in S1 File representing the biological importance of these signatures and their previous implication in liver cancer or any other malignancies. Moreover, they are also enriched in various MSigDB oncogenic signatures (Table L-B in S1 File). Besides, all the identified CpG sites signatures (both for stage classification and cancer v/s normal classification) that are implicated in liver cancer and other cancers are shown in Table M in S1 File.

Further, we proceed to develop models based on multiple RNA transcripts/features selected by different techniques, *i*.*e*. WEKA and F-ANOVA method. In this, an effort has been made to build a prediction model using 35 WEKA selected features (from 147 RNA transcripts) to differentiate liver cancer samples from the normal samples. The SVM based model attains maximum accuracy nearly 99% with AUC 0.99 on both training and independent validation dataset respectively (result not shown). To develop the prediction model based on least number of genes/features, that can categorize samples with reasonably good accuracy; we firstly selected 25, 20, 15, 10 and 5 feature sets (results not shown) using F-ANOVA method. Afterwards, prediction models developed on the training dataset and independent validation dataset. The model’s performance on the independent validation dataset is similar to the performance obtained using CpG sites. Interestingly models based on this feature set almost performed comparably to that of model based on 35 features selected by WEKA. 5-RNA feature set (LCN-5-RNA) based SVM prediction model categorize samples with accuracy 98.53 and AUC 0.97 of training dataset and with accuracy 97.65 and AUC 0.93 of the independent validation dataset. Additionally, models based on the Random forest, SMO, Naïve Bayes, and J48 algorithms also performed almost equal to that of SVM, as indicated in Table 6. The LCN-5-RNA signature includes *CLEC4G*, *CLEC4M*, *FCN2*, *CLEC1B* and *COLEC10* get downregulated in cancer vs. normal samples, as shown in Fig 4(B).

**Table 6:** The performance of models developed for discriminating LIHC and normal samples using 5 RNA transcripts.

Machine Learning Techniques	Dataset	Performance Measures				
		Sensitivity (%)	Specificity (%)	Accuracy (%)	MCC	AUC with 95% CI
SVM	Training	97.32	97.5	97.35	0.89	0.99 (0.98–0.99)
	Validation	97.33	90	96.47	0.84	0.97 (0.94–1)
Random Forest	Training	97.66	90	96.76	0.85	0.98 (0.94–1)
	Validation	98.67	80	96.47	0.82	0.89 (0.84–0.95)
Naïve Bayes	Training	96.66	97.5	96.76	0.86	0.97 (0.94–0.99)
	Validation	97.33	90	96.47	0.84	0.94 (0.84–1)
SMO	Training	97.32	97.5	97.35	0.89	0.97 (0.94–1)
	Validation	97.33	90	96.47	0.84	0.94 (0.84–1)
J48	Training	96.99	97.5	97.05	0.87	0.96 (0.94–0.99)
	Validation	98.67	90	97.65	0.89	0.94 (0.84–1)

Enrichment analysis of LCN-5-RNA using Enrichr implies the involvement of *CLEC4M* in Phagosome KEGG pathways and *GDF2* in the TGF-beta signaling pathway_Homo sapiens_P00052 (p-value <0.05). *CLEC4M* involved in receptor-mediated virion attachment to host cell (GO:0046813), evasion or tolerance of host defences by the virus (GO:0019049) *etc*. This analysis also denotes their enrichment in various MSigDB Oncogenic Signatures sets such as KRAS, p53, PTEN, mTOR and SNF5 [KRAS.AMP.LUNG_UP.V1_UP, P53_DN.V2_DN, and PTEN_DN.V1_UP], etc. (p-value<0.05).

### Multi-class classification

With an intent to ascertain the features that can segregate normal, early and late stage tissue samples, we have independently selected 33 CpG sites (multiclass-CpG) and 5 RNA transcripts (multiclass-RNA) and subsequently prediction models developed using WEKA.classifiers.meta.MultiClassClassifier of WEKA. The features are selected from 440 CpG sites, and 236 RNA transcripts that are significantly differentially methylated and differentially expressed respectively (between 3 groups: cancer v/s normal and early stage v/s late stage). Here, prediction models developed based on 33 CpG sites (multiclass-CpG) using various techniques of WEKA. Naïve Bayes model is the top performer with an accuracy of 77.43 and 76.54 and weighted average AUC 0.88 and 0.86 on the training and independent validation dataset, respectively (Table 7). Besides, prediction models also devised based on 5 RNA transcripts (multiclass-RNA); the Naïve Bayes model using 5 RNA transcripts achieves maximum performance with an accuracy of 72.73% and 72.84 and weighted average AUC 0.81 and 0.80 on the training and an independent validation dataset respectively (Table 7). The boxplots illustrate the methylation and expression pattern of 33 CpG sites and 5 RNA transcripts (Figure G in S2 File). Additionally, we also developed prediction models based on 284 features selected by WEKA from all 60,483 RNA transcripts using different techniques. Here, the model based on Random forest classifies samples with the accuracy of 78.99% and 70.37%, weighted average AUC 0.88 and 0.80 of training and independent datasets (Table N in S1 File). Complete results of prediction models based on 33 CpG sites 5 and 284 RNA transcripts using various methods implementing WEKA have been shown in the table (Table O in S1 File).

**Table 7:** The performance Naïve Bayes model in the form of confusion matrix developed for classifying normal, early and late stage samples. The model was developed using 33 CpG sites (multiclass-CpG) and 5 RNA transcripts (multiclass-RNA).

**33CpG sites**					
**Training dataset**					
			Actual	Accuracy	Weighted average ROC
Late Stage	Early Stage	Normal			
102	36	3	Late Stage	77.43	0.88
29	107	2	Early Stage		
0	2	38	Normal		
**Validation dataset**					
			**Actual**	Accuracy	Weighted average ROC
Late Stage	Early Stage	Normal			
25	10	1	Late Stage	76.54	0.86
7	27	1	Early Stage		
0	0	10	Normal		
**5 RNA transcripts**					
**Training dataset**					
			**Actual**	Accuracy	Weighted average ROC
Late Stage	Early Stage	Normal			
87	54	0	Late Stage	72.73	0.81
33	105	0	Early Stage		
0	0	40	Normal		
**Validation dataset**					
Predicted as			**Actual**	Accuracy	Weighted average ROC
Late Stage	Early Stage	Normal			
18	18	0	Late Stage	72.84	0.80
4	31	0	Early Stage		
0	0	10	Normal		

Enrichment analysis of multiclass-RNA is represented in Figures (Figure H in S2 File). *MT1E*, *CNDP1*, *C7*, *GMNN*, and *EEF1A2* are among the 5 RNA transcripts identified in multiclass classification. Enrichment analysis indicated upregulated genes such as *GMNN* and *EEF1A2*, are enriched in various growth promoting processes and pathways, while downregulated genes like *CNDP1* and *MT1E* enriched in negative regulators of growth and *C7* enriched in innate immunity pathways respectively. Multiclass-RNA signature with their biological importance is tabulated in Table P in S1 File.

### Multiclass-stage classification

To identify the features that can classify stage-I, stage-II and stage-III-IV tissue samples; first, we have selected 25 features (8CpG sites + 17RNA transcripts) using WEKA and also employed 51 hybrid features (which were chosen to distinguish early and late stage). Further, prediction models developed using these features implementing WEKA.classifiers.meta.MultiClassClassifier of WEKA. The Naïve Bayes prediction model based on 51 features attained moderate performance to classify all three stages with an accuracy of 59.13% and 66.19% with weighted average AUC 0.79 and 0.84 on the training and validation dataset, respectively; while, other models and the models based on 25 features have lower performance (Table Q in S1 File). Furthermore, as number of stage-IV samples is small; thus, we have also tried to classify only stage-I, stage-II and stage-III samples after removing stage-IV samples. The performance doesn’t improve to any remarkable extent (Table R in S1 File).

### Implementation of the web server

To serve the scientific community, we developed CancerLSP (Liver cancer stage prediction), a web server that executes in silico prediction models developed in the present study for prediction of cancer status, *i*.*e*. stage and analysis using the methylation profiling and RNA expression data. Further, CancerLSP mainly has two modules; Prediction Module and Data Analysis Module.

#### Prediction module

This module permits the users to predict the disease status, *i*.*e*. cancerous or normal and stage of cancer of sample using methylation beta values of CpG sites and FPKM quantification values of RNA transcripts. Here, the user required to submit FPKM values of RNA markers (RNA transcripts) and methylation beta values of marker CpG sites of every subject. The output result displays a list for patient samples and corresponding predicted status of cancer or normal and early or late stage. Moreover, the user can select among the models developed from LS-CpG-WEKA, LS-RNA-WEKA LS-CpG-RNA-hybrid, LCN-5-CpG, LCN-5-RNA, multiclass-RNA, and multiclass-RNA which have been identified as putative markers sets for stage progression of LIHC.

#### Data Analysis Module

This module is beneficial in assessing the role of each RNA transcript and CpG site to distinguish early stage from the late stage. In addition, it provides a *p*-value for every feature that indicates whether there is a significant difference in the methylation pattern of CpG site or RNA expression in early and late stage. Furthermore, it also provides threshold-based ROC of each feature along with mean methylation of CpG site and mean expression of that RNA transcript in the early and late stage of LIHC. This web server is available from URL (http://webs.iiitd.edu.in/raghava/cancerlsp/) for the scientific community.

## Discussion

LIHC has become a major threat worldwide owing to its high morbidity and mortality rate. Furthermore, the appropriate treatment options are affected not only by the degree of liver dysfunction but also by tumor stage [56]. Therefore, accurate stage classification and prediction of disease at an early stage are crucial for patient management. Hence, the primary goal of the present study is to identify the genomic and epigenomic features that distinguish the early stage from the late stage of LIHC using TCGA cohort. To decipher the contribution of a single feature (CpG site or gene) in the prediction of the early stage, we ranked differentially methylated CpG sites and differentially expressed transcripts based on the AUC using a simple threshold-based classification method.

Two highest scoring hypomethylated CpG sites in early stage *i*.*e*. cg07132710 and cg11232136 (LS-CPG-AUC signatures) are associated with *GATA2* and *ZNF566* genes respectively. While, four hypermethylated CpG sites in early stage (cg18578954, cg07402003 cg16657244, cg06176471) are associated with *TRIM27*, *TOX3*, *NOLC1*, and *ATP1B1*. In the literature, it has been shown that these genes are epigenetically regulated and associated with cancer progression in different malignancies [57–63].

On ranking the gene expression of all the transcripts, we found *NCAPH*, *CYP4A*, *GLYATL1*, *HILPDA*, *CANT1*, *SLC2A2*, *ALDOA*, *CNFN*, *A1BG*, *CYP2B6*, *HN1*, and *ITIH1* were among top-ranking genes based on AUC. Previously, different reports uncover their involvement in a variety of malignancies [64–71]. Evidently, recent studies have revealed the association of *HN1* and *SLC2A2* with the survival of LIHC patients and *ITIH1* with the progression of LIHC [72–74]. Aberrant expression of *ALDOA*, *EFNA3*, and *FTCD* was also observed in LIHC [75–78].

As cancer is a complex disease for which a single feature is not enough to define its progression. Therefore, we used state of the art machine learning techniques to elucidate key features associated with LIHC stage and further improve the performance of the models. We found 21 CpG (LS-CPG-WEKA) methylation sites that achieved the AUC of 0.78 on validation data to discriminate early stage from the late stage. Among LS-CPG-WEKA signature CpG sites such as cg16657244, cg11232136, cg11023721, and cg27111890 are associated with *NOLC1*, *ZNF566*, *SMAD7* and UBASH3A respectively. Previously the aberrant methylation pattern of these genes has been observed in different types of cancers [62].

We also report 30 RNA transcripts (LS-RNA-WEKA) that achieved the AUC of 0.77 on the validation dataset. Among the 15 protein-coding transcripts in LS-RNA-WEKA signature, *DCK*, *CDCA5*, *RPP25*, *FUT11*, *NECAB1*, *FNTB*, *ZNF576*, *NETO2*, and *ELOVL3* are observed as upregulated genes in late stage and are involved in cell division processes, transcriptional regulation, while the genes downregulated in late stage such as *MAT1A*, *LCAT*, *CFHR3* are involved in normal functioning of liver including lipid and lipoprotein metabolism [79] (S6 Table). Previously, different studies have shown the role of *MAT1A* in LIHC [80–82]. Two of the genes (*CDCA5* and *RAMP3*) in this biomarker panel have already been identified as prognostic markers in LIHC in the literature [83,84]. In the recent past, the role of pseudogenes has been revealed in the pathogenesis of cancer including LIHC [85–88]. LS-RNA-WEKA also contains 12 pseudogenes (9 processed, two unprocessed and one transcribed processed pseudogenes) including RP3-375P9.2, GAPDHP63, PTMAP2, RPS3P7, FABP5P3, HNRNPA1P37, AC018712.2 *etc*. They are observed as significantly upregulated in the late stage of LIHC samples in our study.

Models based on 21 methylation CpG sites and 30 RNA transcripts achieved reasonable performance independently for classifying early and late stage tissue samples. On combining these features or markers, hybrid prediction models developed based on 51 features can classify samples of independent validation dataset with higher performance with an accuracy of nearly 78% and AUC 0.82.

In addition to stage classification, we have also elucidated vital features that are differentially expressed in LIHC as compared to normal samples. Using a simple threshold-based approach, we have identified the top 10 CpG sites and top 10 RNA transcripts that discriminate cancer samples from adjacent normal with AUC greater than 0.9. *CLEC4G* and CpG site cg07274716 (associated with *PITX1*) distinguish cancer samples from normal with AUC 0.98 and 0.97 respectively. Earlier studies have shown the association of downregulation of *CLEC4G* expression with the progression of LIHC [89,90]. Our study corroborates this signature marker in LIHC and further extends the importance of this gene as a diagnostic marker. Also, one of CpG sites cg06353345 that can distinguish cancer normal samples with AUC 0.96 was also earlier observed hypomethylated in LIHC [91]. Previous reports indicate that the hypermethylation of the *PITX1* correlated with the tumor progression HNSCC [92] and ESCC [93]. Similarly, the present study reveals the association hypermethylation of *PITX1* with the progression of LIHC.

Additionally, prediction models developed using multiple features filtered by different techniques to distinguish cancer from control samples. Interestingly, our study has shown that the model based on 5 features i.e. 5 RNA transcripts (CLEC4G, CLEC4M, FCN2, CLEC1B and COLEC10), or 5 CpG sites (cg20855160, cg7614747, cg21009747, cg25792518 and cg03497652) performed quite similar to that of models based on much large dimension of features as indicated by the performance to discriminate cancer and normal samples with 96.47% accuracy and AUC 0.97 on the independent validation dataset. Interestingly in the current study, we observed that *LCAT* gene could classify cancer and normal samples with AUC 0.92, is also identified as one of the important markers (LS-RNA-WEKA) to distinguish early stage samples from the late stage. Thus, our report suggests the role of *LCAT* as an important signature marker in the progression of LIHC. Previously its association was observed with the poor survival of LIHC patients [94].

Our next aim was to enhance our model for the stage classification, so we included the normal samples along with early and late stage samples to develop multiclass prediction models that can separate normal, early stage and late stage tissue samples. The advantage of this model as compared to the binary model is that it also captures the gene expression differences between adjacent normal and early stage samples. Based on the 5 RNA transcripts include *MT1E*, *CNDP1*, *C7*, *GMNN*, and *EEF1A2*, we achieved an accuracy of 72.84% with weighted AUC of 0.81. Further using 33 CpG sites (cg13093389, cg05488681, cg01426968, cg00590251, cg14106046, cg08081390, cg06958636, cg18110553, cg17329745, cg22816909, cg07906520, cg23931819 *etc*) accuracy of 77.54% and weighted AUC 0.86 is obtained. Their enrichment analysis shows that upregulated genes are enriched in various growth enhancing processes and pathways such as positives regulation of different kinase signaling, GTPase; while downregulated genes are enriched in negative regulators of growth processes and innate immunity pathways. This might lay insight towards their involvement in the progression of the cancerous condition.

## Conclusion

In summary, we have achieved only reasonable performance (AUC 0.82) using the hybrid model based on 51 potential markers (include 21 CpG sites and 30 RNA transcripts) to classify early stage from the late stage via our systematic analysis of methylation and gene expression profiles of LIHC tissue despite implementing different approaches. In addition, 5 RNA and 5 CpG sites based prediction models distinguish cancer vs. normal samples with quite high precision. Furthermore, multiclass prediction models based on 5 RNA transcripts and 33 CpG sites attained reasonable performance in categorizing normal, early and late stage samples. Our findings suggest that the stage classification is a quite challenging task in comparison to cancer vs. normal samples classification from their expression and methylation profiling. Moreover, LIHC is a complex disease with various pathological bases; thus, our results raise the hypothesis that the exploration of these potential biomarker combinations might offer more accurate and precise diagnosis LIHC at the early stage.

### Potential implications

We anticipate this study would be beneficial to recognize these new important epigenetic and genomic-based candidate markers for early diagnosis of liver cancer. Their potential as a prognostic marker of LIHC can be further investigated by clinicians and researchers involved in the respective research area. We have integrated all our models based on these potential markers in the form of web server named CancerLSP for the use of scientific community working in the respective research area.

## Limitation of the study

In the current study, we have identified potential biomarkers based on genomic and epigenetic profiles to differentiate the early and late of HCC with reasonable performance. One of the limitations associated with these biomarkers is that they are derived from the tissue samples; which required invasive approaches for their isolation. But, one can employ a similar strategy if sufficient data is available for blood, serum, urine, cell-free DNA etc. to develop non-invasive biomarkers. Furthermore, our study mainly based on TCGA high throughput datasets for both internal and independent validation. These potential biomarkers further need to be validated on other external datasets derived from various other studies to elucidate their clinical utility.

## Supporting information

S1 File Supplementary Tables. (DOC)

S2 File Supplementary Figures. (DOCX)

## Abbreviations

AUC: Area under Receiver Operating Characteristics
CI: Confidence Interval
FDR: False Discovery Rate
J48: decision tree (J48)
MCC: Matthews correlation coefficient
SMO: Sequential minimal optimization
