# Supplementary material for: Classification of early and late stage liver hepatocellular carcinoma patients from their genomics and epigenomics profiles
Source: PLoS One. 2019 Sep 6;14(9):e0221476. doi: 10.1371/journal.pone.0221476 (PMC6730898; doi:10.1371/journal.pone.0221476)
Supplement: S1 File — (DOC) [file pone.0221476.s001.doc]

**Supplementary Information**

**Classification of early and late stage Liver Hepatocellular Carcinoma patients from their genomics and epigenomics profiles.**

Harpreet Kaur^1^, Sherry Bhalla^2,3^, Gajendra P.S. Raghava^2*^

1. Bioinformatics Centre, CSIR-Institute of Microbial Technology, Sector 39A, Chandigarh-160036, India
2. Department of Computational Biology, Indraprastha Institute of Information Technology, New Delhi, India.
3. Centre for Systems Biology and Bioinformatics, Panjab University, Sector 14, Chandigarh-160014, India

**Table A. Top 170 CpG sites (Probe IDs associated with CpG sites) based on single gene threshold model approach to classify early stage and late stage tissue samples.**

| **CpG_site** | **AUC** | **Threshold** | **Mean in stage-I (Beta value)** | **Mean in late stage (Beta value)** | **Gene Symbol** | **FDR** | **Featue type** |
| --- | --- | --- | --- | --- | --- | --- | --- |
| **cg20457523** | 0.66 | 0.83 | 0.84 | 0.77 | *YWHAG* | 0.039 | N_Shelf |
| **cg18563987** | 0.65 | 0.70 | 0.78 | 0.68 | *SAP30BP* | 0.026 | . |
| **cg18578954** | 0.65 | 0.46 | 0.61 | 0.50 | *TRIM27* | 0.045 | N_Shelf |
| **cg22753611** | 0.65 | 0.89 | 0.88 | 0.79 | *CAP2* | 0.026 | . |
| **cg01426968** | 0.65 | 0.56 | 0.62 | 0.50 | *C16orf45;RP11-1021N1.1* | 0.026 | . |
| **cg17799563** | 0.64 | 0.76 | 0.80 | 0.72 | *DDA1* | 0.032 | S_Shore |
| **cg16296417** | 0.64 | 0.48 | 0.69 | 0.56 | *NSMCE2* | 0.026 | . |
| **cg07402003** | 0.64 | 0.78 | 0.82 | 0.74 | *TOX3* | 0.047 | . |
| **cg16657244** | 0.64 | 0.55 | 0.59 | 0.47 | *NOLC1* | 0.026 | N_Shore |
| **cg17609804** | 0.64 | 0.83 | 0.86 | 0.80 | *TBRG4* | 0.050 | N_Shelf |
| **cg21691367** | 0.64 | 0.46 | 0.70 | 0.57 | *MTHFD1L* | 0.034 | . |
| **cg03707168** | 0.64 | 0.43 | 0.53 | 0.39 | *PPP1R15A* | 0.010 | S_Shelf |
| **cg16876964** | 0.64 | 0.90 | 0.83 | 0.87 | *BICD1* | 0.048 | . |
| **cg16442450** | 0.64 | 0.23 | 0.22 | 0.24 | *ANP32A;SPESP1* | 0.035 | Island |
| **cg06176471** | 0.64 | 0.69 | 0.76 | 0.68 | *ATP1B1* | 0.048 | . |
| **cg02386725** | 0.63 | 0.14 | 0.13 | 0.15 | *UHMK1* | 0.035 | Island |
| **cg13837721** | 0.63 | 0.32 | 0.32 | 0.22 | *.* | 0.049 | . |
| **cg00590251** | 0.63 | 0.75 | 0.65 | 0.77 | *STEAP3* | 0.045 | N_Shore |
| **cg14543104** | 0.63 | 0.75 | 0.72 | 0.79 | *.* | 0.049 | N_Shelf |
| **cg20938708** | 0.63 | 0.69 | 0.74 | 0.65 | *RP11-474I11.7;SAP30BP* | 0.049 | . |
| **cg26541218** | 0.63 | 0.56 | 0.67 | 0.55 | *HUS1;PKD1L1* | 0.039 | . |
| **cg13883366** | 0.63 | 0.83 | 0.81 | 0.87 | *SVIL;SVIL-AS1* | 0.049 | . |
| **cg10214581** | 0.63 | 0.60 | 0.66 | 0.57 | *.* | 0.041 | . |
| **cg26726542** | 0.63 | 0.16 | 0.14 | 0.17 | *.* | 0.026 | Island |
| **cg17169982** | 0.63 | 0.84 | 0.81 | 0.84 | *TBCD;ZNF750* | 0.032 | N_Shelf |
| **cg23604151** | 0.63 | 0.81 | 0.77 | 0.83 | *ZFP64* | 0.035 | N_Shelf |
| **cg17259265** | 0.63 | 0.79 | 0.90 | 0.82 | *HYLS1* | 0.026 | N_Shelf |
| **cg27445386** | 0.63 | 0.20 | 0.20 | 0.22 | *CNPY3* | 0.047 | Island |
| **cg25087352** | 0.63 | 0.81 | 0.73 | 0.81 | *THRB* | 0.048 | . |
| **cg13714378** | 0.63 | 0.74 | 0.85 | 0.76 | *.* | 0.032 | . |
| **cg21723559** | 0.63 | 0.41 | 0.61 | 0.50 | *PIGT* | 0.026 | S_Shelf |
| **cg24806326** | 0.63 | 0.60 | 0.71 | 0.62 | *PLCD3* | 0.047 | N_Shore |
| **cg01889574** | 0.63 | 0.33 | 0.54 | 0.40 | *CD82* | 0.032 | S_Shelf |
| **cg01110839** | 0.63 | 0.71 | 0.74 | 0.61 | *SLC39A13* | 0.026 | . |
| **cg24515136** | 0.63 | 0.68 | 0.61 | 0.68 | *RP11-700H6.2* | 0.010 | S_Shelf |
| **cg01826354** | 0.63 | 0.32 | 0.40 | 0.28 | *B3GNTL1* | 0.049 | N_Shelf |
| **cg08140891** | 0.63 | 0.21 | 0.21 | 0.23 | *FLAD1* | 0.049 | . |
| **cg16896313** | 0.63 | 0.71 | 0.78 | 0.68 | *ALDH1A2* | 0.026 | . |
| **cg03519180** | 0.63 | 0.82 | 0.88 | 0.81 | *PDZD8* | 0.026 | . |
| **cg20592700** | 0.63 | 0.18 | 0.21 | 0.25 | *WIPI2* | 0.049 | Island |
| **cg24124954** | 0.63 | 0.55 | 0.64 | 0.55 | *ATP6V1G2-DDX39B* | 0.049 | N_Shore |
| **cg26177760** | 0.63 | 0.55 | 0.63 | 0.55 | *ATP6V1G2-DDX39B* | 0.045 | N_Shore |
| **cg09317128** | 0.63 | 0.29 | 0.58 | 0.46 | *TMEM165* | 0.046 | S_Shelf |
| **cg19426128** | 0.63 | 0.75 | 0.74 | 0.80 | *RP11-161H23.5;TUBA1C* | 0.048 | . |
| **cg12592365** | 0.63 | 0.55 | 0.63 | 0.50 | *RPTOR* | 0.044 | . |
| **cg08162476** | 0.63 | 0.55 | 0.64 | 0.56 | *IQSEC1* | 0.049 | . |
| **cg16997314** | 0.62 | 0.84 | 0.87 | 0.80 | *CCNY* | 0.043 | . |
| **cg21251203** | 0.62 | 0.64 | 0.74 | 0.66 | *NFIB* | 0.048 | . |
| **cg03151540** | 0.62 | 0.24 | 0.24 | 0.27 | *TAPT1;TAPT1-AS1* | 0.044 | Island |
| **cg22321808** | 0.62 | 0.81 | 0.80 | 0.72 | *TTC7A* | 0.049 | . |
| **cg01737507** | 0.62 | 0.26 | 0.25 | 0.27 | *PPIC;RP11-359P5.1* | 0.049 | Island |
| **cg23955575** | 0.62 | 0.86 | 0.85 | 0.84 | *AGAP3* | 0.049 | . |
| **cg17941109** | 0.62 | 0.64 | 0.68 | 0.59 | *ABHD8;CTD-2278I10.4;MRPL34* | 0.038 | S_Shore |
| **cg18954047** | 0.62 | 0.69 | 0.76 | 0.68 | *TRIM27* | 0.049 | N_Shore |
| **cg15542639** | 0.62 | 0.76 | 0.84 | 0.78 | *PANX1* | 0.048 | . |
| **cg26147736** | 0.62 | 0.64 | 0.66 | 0.73 | *KIAA1217* | 0.050 | . |
| **cg01116477** | 0.62 | 0.86 | 0.88 | 0.82 | *C1QTNF3-AMACR* | 0.032 | . |
| **cg16283385** | 0.62 | 0.73 | 0.75 | 0.81 | *TBCEL* | 0.049 | . |
| **cg08463024** | 0.62 | 0.36 | 0.57 | 0.46 | *ATP6V1G2-DDX39B* | 0.045 | N_Shore |
| **cg18110553** | 0.62 | 0.90 | 0.66 | 0.78 | *SAMD4A* | 0.048 | . |
| **cg15553612** | 0.62 | 0.67 | 0.69 | 0.77 | *TBX15* | 0.049 | N_Shore |
| **cg08145798** | 0.62 | 0.63 | 0.73 | 0.65 | *CDC42BPG* | 0.048 | N_Shore |
| **cg22605785** | 0.62 | 0.86 | 0.87 | 0.85 | *.* | 0.044 | . |
| **cg22933449** | 0.62 | 0.57 | 0.68 | 0.59 | *.* | 0.032 | . |
| **cg23497752** | 0.62 | 0.33 | 0.44 | 0.33 | *FLNA* | 0.049 | N_Shore |
| **cg13369981** | 0.62 | 0.18 | 0.20 | 0.23 | *MEPCE;ZCWPW1* | 0.050 | Island |
| **cg01319323** | 0.62 | 0.78 | 0.83 | 0.74 | *SH3GLB2* | 0.038 | N_Shore |
| **cg00967294** | 0.62 | 0.91 | 0.88 | 0.91 | *ARHGAP26* | 0.047 | . |
| **cg12377026** | 0.62 | 0.86 | 0.80 | 0.84 | *ZFP62* | 0.045 | . |
| **cg08168844** | 0.62 | 0.84 | 0.87 | 0.80 | *AC009133.12;CTD-2574D22.6;MVP;PAGR1* | 0.050 | S_Shelf |
| **cg04389328** | 0.62 | 0.13 | 0.15 | 0.19 | *RANBP1;TRMT2A* | 0.045 | Island |
| **cg27652459** | 0.62 | 0.57 | 0.68 | 0.62 | *ARHGAP1* | 0.043 | . |
| **cg02838589** | 0.62 | 0.80 | 0.88 | 0.82 | *UBE2C* | 0.030 | S_Shelf |
| **cg11998497** | 0.62 | 0.77 | 0.70 | 0.78 | *ARHGEF4;SCARNA4* | 0.032 | . |
| **cg08081390** | 0.62 | 0.81 | 0.86 | 0.78 | *FUS;RP11-388M20.6* | 0.047 | S_Shelf |
| **cg14869505** | 0.62 | 0.82 | 0.83 | 0.77 | *ATP5B* | 0.045 | N_Shore |
| **cg12444761** | 0.62 | 0.84 | 0.77 | 0.84 | *SPRED1* | 0.050 | . |
| **cg22159341** | 0.62 | 0.78 | 0.77 | 0.83 | *CACNB4* | 0.049 | . |
| **cg25609878** | 0.62 | 0.50 | 0.65 | 0.56 | *MTHFD2L* | 0.050 | . |
| **cg26111575** | 0.62 | 0.36 | 0.54 | 0.45 | *TACC2* | 0.049 | . |
| **cg21437722** | 0.62 | 0.61 | 0.65 | 0.70 | *FAM222B* | 0.049 | . |
| **cg20487932** | 0.62 | 0.87 | 0.87 | 0.84 | *SSH1* | 0.048 | . |
| **cg24844545** | 0.62 | 0.67 | 0.70 | 0.65 | *NPPA* | 0.048 | . |
| **cg20122586** | 0.62 | 0.78 | 0.84 | 0.79 | *USP46* | 0.026 | N_Shelf |
| **cg17884016** | 0.62 | 0.62 | 0.70 | 0.61 | *CCZ1B* | 0.045 | N_Shore |
| **cg06153102** | 0.62 | 0.69 | 0.64 | 0.71 | *.* | 0.048 | N_Shelf |
| **cg15172739** | 0.62 | 0.48 | 0.61 | 0.51 | *SNHG1;SNORD30* | 0.049 | N_Shelf |
| **cg23919534** | 0.62 | 0.16 | 0.16 | 0.23 | *ESPN;RP1-202O8.2* | 0.039 | Island |
| **cg20539307** | 0.62 | 0.80 | 0.84 | 0.80 | *CTSB* | 0.049 | . |
| **cg13724820** | 0.62 | 0.64 | 0.70 | 0.62 | *ABCG8* | 0.026 | . |
| **cg12473916** | 0.62 | 0.25 | 0.32 | 0.24 | *SHC1* | 0.039 | N_Shelf |
| **cg14106046** | 0.62 | 0.33 | 0.47 | 0.34 | *B3GNTL1* | 0.045 | N_Shelf |
| **cg13093389** | 0.61 | 0.57 | 0.71 | 0.63 | *ARHGEF40* | 0.047 | N_Shore |
| **cg01023769** | 0.61 | 0.16 | 0.19 | 0.14 | *ARL13B* | 0.048 | N_Shore |
| **cg05132999** | 0.61 | 0.73 | 0.76 | 0.66 | *TTC39A;TTC39A-AS1* | 0.045 | S_Shore |
| **cg19700380** | 0.61 | 0.74 | 0.70 | 0.77 | *PCGF2* | 0.043 | . |
| **cg06931405** | 0.61 | 0.70 | 0.74 | 0.81 | *PTK2* | 0.048 | . |
| **cg04835383** | 0.61 | 0.83 | 0.87 | 0.82 | *TRIM27* | 0.042 | N_Shelf |
| **cg22205573** | 0.61 | 0.80 | 0.79 | 0.73 | *.* | 0.040 | . |
| **cg06626750** | 0.61 | 0.37 | 0.66 | 0.54 | *RP11-159D12.5;SRSF1* | 0.047 | N_Shore |
| **cg22627427** | 0.61 | 0.71 | 0.76 | 0.68 | *DKFZP434K028;MYRF* | 0.047 | S_Shelf |
| **cg10673265** | 0.61 | 0.58 | 0.66 | 0.58 | *SLC39A7* | 0.049 | S_Shore |
| **cg24830314** | 0.61 | 0.15 | 0.14 | 0.15 | *UBE3D* | 0.047 | Island |
| **cg15867698** | 0.61 | 0.82 | 0.83 | 0.80 | *ACTN1* | 0.045 | . |
| **cg01941018** | 0.61 | 0.14 | 0.13 | 0.15 | *C1S;LPCAT3* | 0.049 | Island |
| **cg00506299** | 0.61 | 0.61 | 0.77 | 0.68 | *RFTN1* | 0.046 | . |
| **cg26929645** | 0.61 | 0.83 | 0.83 | 0.87 | *ZFP62* | 0.045 | . |
| **cg05488681** | 0.61 | 0.72 | 0.76 | 0.68 | *.* | 0.045 | . |
| **cg08710987** | 0.61 | 0.62 | 0.62 | 0.53 | *CCZ1* | 0.048 | S_Shore |
| **cg21498475** | 0.61 | 0.66 | 0.74 | 0.67 | *SLC8B1* | 0.048 | . |
| **cg00267196** | 0.61 | 0.81 | 0.79 | 0.86 | *ZFAT* | 0.048 | . |
| **cg10727416** | 0.61 | 0.80 | 0.82 | 0.87 | *ZNF529* | 0.044 | S_Shelf |
| **cg12104689** | 0.61 | 0.91 | 0.92 | 0.90 | *APPBP2* | 0.026 | N_Shelf |
| **cg26009832** | 0.61 | 0.78 | 0.85 | 0.76 | *ATP1B1* | 0.042 | . |
| **cg23677197** | 0.61 | 0.88 | 0.75 | 0.82 | *BBS5;RP11-724O16.1* | 0.039 | S_Shelf |
| **cg17329745** | 0.61 | 0.84 | 0.86 | 0.89 | *AC096587.1;CWC22* | 0.047 | . |
| **cg23460551** | 0.61 | 0.72 | 0.74 | 0.81 | *.* | 0.045 | . |
| **cg15117681** | 0.61 | 0.64 | 0.67 | 0.60 | *CSNK1D;RP13-516M14.10;SLC16A3* | 0.044 | N_Shore |
| **cg17230535** | 0.61 | 0.76 | 0.85 | 0.78 | *AVL9;DPY19L1P1* | 0.049 | . |
| **cg19615651** | 0.61 | 0.77 | 0.74 | 0.66 | *AL021546.6;TRIAP1* | 0.028 | N_Shore |
| **cg20270775** | 0.61 | 0.20 | 0.18 | 0.20 | *CMC1* | 0.048 | Island |
| **cg24368588** | 0.61 | 0.21 | 0.20 | 0.22 | *ARFGAP1;NKAIN4* | 0.049 | Island |
| **cg27111890** | 0.61 | 0.67 | 0.62 | 0.73 | *UBASH3A* | 0.038 | . |
| **cg07111049** | 0.61 | 0.82 | 0.80 | 0.85 | *RP11-33B1.1* | 0.049 | S_Shelf |
| **cg04105250** | 0.61 | 0.32 | 0.18 | 0.25 | *GAD1* | 0.049 | Island |
| **cg14852276** | 0.61 | 0.79 | 0.81 | 0.73 | *IQCG* | 0.045 | N_Shelf |
| **cg11807153** | 0.61 | 0.55 | 0.55 | 0.44 | *VTRNA1-2* | 0.048 | . |
| **cg08782002** | 0.61 | 0.87 | 0.88 | 0.83 | *CCDC47* | 0.039 | N_Shelf |
| **cg00470154** | 0.61 | 0.82 | 0.74 | 0.81 | *.* | 0.047 | . |
| **cg01939872** | 0.61 | 0.87 | 0.88 | 0.90 | *TXNDC9* | 0.049 | N_Shelf |
| **cg06858263** | 0.61 | 0.69 | 0.73 | 0.69 | *DNAJB6* | 0.049 | . |
| **cg02985240** | 0.61 | 0.66 | 0.70 | 0.79 | *ARID4B* | 0.035 | . |
| **cg07447769** | 0.61 | 0.70 | 0.72 | 0.64 | *RP11-307N16.6;SPATA13* | 0.049 | . |
| **cg10738119** | 0.61 | 0.83 | 0.82 | 0.77 | *YWHAG* | 0.049 | N_Shore |
| **cg23102386** | 0.61 | 0.74 | 0.77 | 0.83 | *STAMBP* | 0.049 | N_Shore |
| **cg16659908** | 0.61 | 0.85 | 0.85 | 0.82 | *AP000487.6;PPFIA1* | 0.048 | . |
| **cg23931819** | 0.61 | 0.78 | 0.85 | 0.75 | *ACAP3;PUSL1* | 0.050 | Island |
| **cg03542686** | 0.61 | 0.70 | 0.72 | 0.66 | *CAPZB* | 0.044 | . |
| **cg05491930** | 0.61 | 0.19 | 0.20 | 0.22 | *PNISR;RP11-98I9.4* | 0.048 | Island |
| **cg17069533** | 0.61 | 0.71 | 0.84 | 0.76 | *PRKCA* | 0.047 | . |
| **cg00214688** | 0.61 | 0.88 | 0.90 | 0.88 | *ITGB1* | 0.045 | . |
| **cg07906520** | 0.61 | 0.11 | 0.13 | 0.24 | *PPP1R16B* | 0.049 | S_Shore |
| **cg24031764** | 0.61 | 0.71 | 0.77 | 0.69 | *TPRG1* | 0.049 | . |
| **cg18446110** | 0.61 | 0.71 | 0.72 | 0.64 | *SLC23A1* | 0.048 | S_Shore |
| **cg12595697** | 0.61 | 0.94 | 0.96 | 0.94 | *EEFSEC* | 0.049 | . |
| **cg22491320** | 0.61 | 0.63 | 0.51 | 0.61 | *NCKAP5* | 0.048 | . |
| **cg11232136** | 0.60 | 0.90 | 0.92 | 0.89 | *ZNF566* | 0.028 | . |
| **cg01055099** | 0.60 | 0.84 | 0.88 | 0.84 | *LA16c-306E5.2;NAA60* | 0.049 | . |
| **cg03108697** | 0.60 | 0.94 | 0.89 | 0.92 | *SWAP70* | 0.042 | . |
| **cg05934196** | 0.60 | 0.20 | 0.19 | 0.21 | *RP11-875O11.2;TNFRSF10B* | 0.049 | Island |
| **cg04203298** | 0.60 | 0.17 | 0.18 | 0.22 | *AP000640.10;STX3* | 0.029 | N_Shore |
| **cg18107290** | 0.60 | 0.77 | 0.83 | 0.78 | *DLST* | 0.049 | S_Shelf |
| **cg05819268** | 0.60 | 0.87 | 0.84 | 0.87 | *CACNG1* | 0.044 | Island |
| **cg12754671** | 0.60 | 0.31 | 0.29 | 0.31 | *NDUFS2* | 0.049 | Island |
| **cg25997426** | 0.60 | 0.80 | 0.75 | 0.81 | *HSF5;MTMR4* | 0.049 | S_Shore |
| **cg22103441** | 0.60 | 0.13 | 0.11 | 0.09 | *.* | 0.044 | . |
| **cg06958636** | 0.60 | 0.55 | 0.53 | 0.44 | *EFCAB2* | 0.049 | . |
| **cg08039701** | 0.60 | 0.14 | 0.14 | 0.17 | *GFM2;NSA2* | 0.050 | Island |
| **cg06239143** | 0.60 | 0.77 | 0.81 | 0.86 | *AC090154.1;C8orf44;C8orf44-SGK3* | 0.049 | . |
| **cg12390237** | 0.60 | 0.75 | 0.71 | 0.64 | *NOP10;NUTM1* | 0.049 | S_Shelf |
| **cg20326682** | 0.60 | 0.80 | 0.85 | 0.78 | *PRKCA* | 0.049 | . |
| **cg25076980** | 0.60 | 0.78 | 0.82 | 0.79 | *CSNK2B;CSNK2B-LY6G5B-562;LY6G5B* | 0.047 | S_Shelf |
| **cg24775180** | 0.60 | 0.91 | 0.92 | 0.88 | *PUS1* | 0.048 | . |
| **cg15323253** | 0.60 | 0.77 | 0.84 | 0.78 | *.* | 0.049 | . |
| **cg09293122** | 0.60 | 0.84 | 0.82 | 0.78 | *GATS;STAG3* | 0.047 | . |
| **cg06185532** | 0.60 | 0.79 | 0.83 | 0.79 | *ZFHX3* | 0.049 | . |
| **cg18056097** | 0.60 | 0.77 | 0.78 | 0.82 | *CHMP4B* | 0.049 | . |
| **cg05934333** | 0.60 | 0.77 | 0.77 | 0.83 | *.* | 0.049 | . |
| **cg07132710** | 0.60 | 0.43 | 0.38 | 0.45 | *GATA2* | 0.048 | N_Shelf |
| **cg03542721** | 0.60 | 0.16 | 0.15 | 0.17 | *RP11-875O11.2;TNFRSF10B* | 0.045 | Island |

**Table B. The Performance of stage classification models using 170 CpG sites or LS-CPG-AUC.**

| **Technique** | **Dataset** | **Performance Measures** | | | | |
| --- | --- | --- | --- | --- | --- | --- |
|  |  | **Sensitivity** | **Specificity** | **Accuracy (%)** | **MCC** | **AUC with 95% CI** |
| **SVM** | Training | 77.54 | 73.05 | 75.27 | 0.51 | 0.79 (0.74-0.85) |
|  | Validation | 57.14 | 69.44 | 63.38 | 0.27 | 0.66 (0.53-0.79) |
| **Random Forest** | Training | 68.84 | 71.63 | 70.25 | 0.4 | 0.77 (0.67-0.87) |
|  | Validation | 60 | 52.78 | 56.34 | 0.13 | 0.6 (0.59 -0.61) |
| **Naïve bayes** | Training | 77.54 | 65.25 | 71.33 | 0.43 | 0.73 (0.67-0.76) |
|  | Validation | 60 | 75 | 67.61 | 0.35 | 0.67(0.57-0.78) |
| **SMO** | Training | 78.99 | 69.5 | 74.19 | 0.49 | 0.74 (0.69-0.78) |
|  | Validation | 51.43 | 61.11 | 56.34 | 0.13 | 0.56 (0.45-0.68) |
| **J48** | Training | 68.84 | 63.12 | 65.95 | 0.32 | 0.64 (0.58-0.69) |
|  | Validation | 51.43 | 55.56 | 53.52 | 0.07 | 0.53 (0.42-0.65) |

**Table C. Top 99 RNA transcripts or LS-RNA-AUC ( AUC = or > 0.6) single gene threshold based models for stage classification.**

| **Ensemble ID** | **Gene symbol** | **AUC** | **Threshold (Log2(FPKM))** | **Mean in early stage (Log2(FPKM))** | **Mean in Late stage (Log2(FPKM))** | **FDR** | **Class** |
| --- | --- | --- | --- | --- | --- | --- | --- |
| **ENSG00000121152.8** | *NCAPH* | 0.66 | 1.45 | 1.07 | 1.49 | 0.0097 | protein coding |
| **ENSG00000162365.10** | *CYP4A22* | 0.66 | 3.20 | 4.12 | 3.01 | 0.0006 | protein coding |
| **ENSG00000025423.10** | *HSD17B6* | 0.65 | 6.69 | 7.21 | 5.99 | 0.0019 | protein coding |
| **ENSG00000166840.12** | *GLYATL1* | 0.65 | 2.50 | 3.68 | 2.75 | 0.0019 | protein coding |
| **ENSG00000160282.12** | *FTCD* | 0.65 | 5.55 | 6.32 | 5.28 | 0.0035 | protein coding |
| **ENSG00000135245.9** | *HILPDA* | 0.65 | 1.81 | 1.41 | 1.96 | 0.0015 | protein coding |
| **ENSG00000197408.7** | *CYP2B6* | 0.65 | 2.70 | 4.17 | 3.11 | 0.0067 | protein coding |
| **ENSG00000258867.4** | *LINC01146* | 0.65 | 1.91 | 2.34 | 1.70 | 0.0074 | lincRNA |
| **ENSG00000171302.15** | *CANT1* | 0.65 | 3.44 | 3.20 | 3.52 | 0.0075 | protein coding |
| **ENSG00000189159.14** | *HN1* | 0.64 | 3.94 | 3.36 | 3.86 | 0.0067 | protein coding |
| **ENSG00000149925.15** | *ALDOA* | 0.64 | 5.94 | 5.38 | 6.00 | 0.0066 | protein coding |
| **ENSG00000072080.9** | *SPP2* | 0.64 | 3.90 | 5.40 | 3.89 | 0.0006 | protein coding |
| **ENSG00000105427.8** | *CNFN* | 0.64 | 1.10 | 0.72 | 1.24 | 0.0067 | protein coding |
| **ENSG00000161267.10** | *BDH1* | 0.64 | 3.68 | 4.14 | 3.44 | 0.0022 | protein coding |
| **ENSG00000163581.12** | *SLC2A2* | 0.64 | 6.20 | 6.54 | 5.65 | 0.0094 | protein coding |
| **ENSG00000055957.9** | *ITIH1* | 0.64 | 8.14 | 8.27 | 7.37 | 0.0069 | protein coding |
| **ENSG00000121410.10** | *A1BG* | 0.64 | 4.04 | 4.43 | 3.50 | 0.0019 | protein coding |
| **ENSG00000143590.12** | *EFNA3* | 0.64 | 1.29 | 0.95 | 1.45 | 0.0006 | protein coding |
| **ENSG00000175711.7** | *B3GNTL1* | 0.64 | 0.87 | 0.72 | 0.94 | 0.0100 | protein coding |
| **ENSG00000159650.7** | *UROC1* | 0.64 | 3.00 | 2.98 | 1.94 | 0.0069 | protein coding |
| **ENSG00000115163.13** | *CENPA* | 0.64 | 1.52 | 0.96 | 1.41 | 0.0079 | protein coding |
| **ENSG00000147100.8** | *SLC16A2* | 0.64 | 3.29 | 3.94 | 3.31 | 0.0094 | protein coding |
| **ENSG00000113905.4** | *HRG* | 0.64 | 6.81 | 8.03 | 6.53 | 0.0067 | protein coding |
| **ENSG00000130988.11** | *RGN* | 0.64 | 4.88 | 5.55 | 4.80 | 0.0033 | protein coding |
| **ENSG00000079462.6** | *PAFAH1B3* | 0.64 | 3.38 | 2.85 | 3.52 | 0.0069 | protein coding |
| **ENSG00000255974.5** | *CYP2A6* | 0.64 | 4.01 | 6.26 | 4.53 | 0.0100 | protein coding |
| **ENSG00000175003.11** | *SLC22A1* | 0.63 | 4.61 | 5.12 | 3.63 | 0.0067 | protein coding |
| **ENSG00000188807.11** | *TMEM201* | 0.63 | 1.50 | 1.13 | 1.43 | 0.0066 | protein coding |
| **ENSG00000174371.15** | *EXO1* | 0.63 | 1.05 | 0.79 | 1.15 | 0.0076 | protein coding |
| **ENSG00000166851.13** | *PLK1* | 0.63 | 1.43 | 1.25 | 1.75 | 0.0096 | protein coding |
| **ENSG00000187048.11** | *CYP4A11* | 0.63 | 4.21 | 5.93 | 4.65 | 0.0007 | protein coding |
| **ENSG00000138109.9** | *CYP2C9* | 0.63 | 4.52 | 6.33 | 5.07 | 0.0067 | protein coding |
| **ENSG00000163170.10** | *BOLA3* | 0.63 | 2.70 | 2.27 | 2.56 | 0.0081 | protein coding |
| **ENSG00000249948.5** | *GBA3* | 0.63 | 1.40 | 2.91 | 2.04 | 0.0069 | Polymorphic pseudogene |
| **ENSG00000175336.9** | *APOF* | 0.63 | 3.42 | 4.57 | 3.49 | 0.0069 | protein coding |
| **ENSG00000139547.7** | *RDH16* | 0.63 | 3.85 | 4.90 | 3.82 | 0.0079 | protein coding |
| **ENSG00000068489.11** | *PRR11* | 0.63 | 1.12 | 0.93 | 1.37 | 0.0067 | protein coding |
| **ENSG00000103222.17** | *ABCC1* | 0.63 | 1.31 | 1.13 | 1.61 | 0.0100 | protein coding |
| **ENSG00000167536.12** | *DHRS13* | 0.63 | 0.94 | 0.88 | 1.19 | 0.0053 | protein coding |
| **ENSG00000141485.14** | *SLC13A5* | 0.63 | 3.40 | 4.55 | 3.64 | 0.0100 | protein coding |
| **ENSG00000165140.8** | *FBP1* | 0.63 | 5.65 | 6.85 | 5.94 | 0.0100 | protein coding |
| **ENSG00000074800.12** | *ENO1* | 0.63 | 7.95 | 7.19 | 7.78 | 0.0023 | protein coding |
| **ENSG00000092445.10** | *TYRO3* | 0.63 | 1.02 | 0.58 | 0.99 | 0.0069 | protein coding |
| **ENSG00000160087.19** | *UBE2J2* | 0.63 | 3.05 | 2.77 | 3.05 | 0.0074 | protein coding |
| **ENSG00000204653.8** | *ASPDH* | 0.62 | 3.72 | 4.25 | 3.33 | 0.0072 | protein coding |
| **ENSG00000108883.11** | *EFTUD2* | 0.62 | 2.93 | 2.83 | 3.09 | 0.0095 | protein coding |
| **ENSG00000271936.1** | *RP11-443B20.1* | 0.62 | 0.60 | 0.39 | 0.63 | 0.0035 | Antisense |
| **ENSG00000140993.9** | *TIGD7* | 0.62 | 0.55 | 0.39 | 0.52 | 0.0079 | protein coding |
| **ENSG00000123453.15** | *SARDH* | 0.62 | 3.60 | 4.24 | 3.67 | 0.0074 | protein coding |
| **ENSG00000154839.8** | *SKA1* | 0.62 | 1.52 | 1.00 | 1.46 | 0.0079 | protein coding |
| **ENSG00000100024.13** | *UPB1* | 0.62 | 5.03 | 4.60 | 3.63 | 0.0022 | protein coding |
| **ENSG00000126231.12** | *PROZ* | 0.62 | 3.82 | 3.45 | 2.64 | 0.0046 | protein coding |
| **ENSG00000184661.12** | *CDCA2* | 0.62 | 0.63 | 0.48 | 0.77 | 0.0074 | protein coding |
| **ENSG00000147647.11** | *DPYS* | 0.62 | 5.22 | 5.71 | 4.74 | 0.0067 | protein coding |
| **ENSG00000140284.9** | *SLC27A2* | 0.62 | 4.94 | 5.48 | 4.62 | 0.0053 | protein coding |
| **ENSG00000103512.13** | *NOMO1* | 0.62 | 2.82 | 2.60 | 2.87 | 0.0074 | protein coding |
| **ENSG00000118271.8** | *TTR* | 0.62 | 9.07 | 9.33 | 8.07 | 0.0069 | protein coding |
| **ENSG00000161800.11** | *RACGAP1* | 0.62 | 1.90 | 1.70 | 2.14 | 0.0100 | protein coding |
| **ENSG00000167881.13** | *SRP68* | 0.62 | 3.36 | 3.28 | 3.53 | 0.0079 | protein coding |
| **ENSG00000106348.15** | *IMPDH1* | 0.62 | 2.71 | 1.64 | 2.20 | 0.0084 | protein coding |
| **ENSG00000068097.13** | *HEATR6* | 0.62 | 1.68 | 1.29 | 1.52 | 0.0100 | protein coding |
| **ENSG00000163535.16** | *SGOL2* | 0.62 | 0.95 | 0.59 | 0.86 | 0.0069 | protein coding |
| **ENSG00000184999.10** | *SLC22A10* | 0.62 | 2.50 | 2.48 | 1.78 | 0.0100 | protein coding |
| **ENSG00000159399.8** | *HK2* | 0.62 | 1.20 | 0.69 | 1.28 | 0.0074 | protein coding |
| **ENSG00000247095.2** | *MIR210HG* | 0.62 | 1.21 | 0.74 | 1.16 | 0.0069 | lincRNA |
| **ENSG00000134538.2** | *SLCO1B1* | 0.62 | 4.40 | 5.53 | 4.61 | 0.0067 | protein coding |
| **ENSG00000129195.14** | *FAM64A* | 0.62 | 0.70 | 0.38 | 0.72 | 0.0074 | protein coding |
| **ENSG00000169740.12** | *ZNF32* | 0.62 | 3.50 | 3.59 | 3.90 | 0.0095 | protein coding |
| **ENSG00000011426.9** | *ANLN* | 0.62 | 1.65 | 1.09 | 1.59 | 0.0074 | protein coding |
| **ENSG00000117601.12** | *SERPINC1* | 0.62 | 9.43 | 10.20 | 9.00 | 0.0095 | protein coding |
| **ENSG00000162063.11** | *CCNF* | 0.62 | 1.38 | 0.98 | 1.30 | 0.0099 | protein coding |
| **ENSG00000230626.3** | *RP11-286H14.4* | 0.62 | 0.20 | 0.14 | 0.21 | 0.0095 | Transcribed processed pseudogene |
| **ENSG00000126787.11** | *DLGAP5* | 0.62 | 1.47 | 0.98 | 1.41 | 0.0088 | protein coding |
| **ENSG00000165775.16** | *FUNDC2* | 0.62 | 2.88 | 2.19 | 2.51 | 0.0074 | protein coding |
| **ENSG00000138778.10** | *CENPE* | 0.62 | 0.94 | 0.47 | 0.73 | 0.0094 | protein coding |
| **ENSG00000134297.6** | *PLEKHA8P1* | 0.62 | 1.31 | 0.86 | 1.09 | 0.0069 | Transcribed processed pseudogene |
| **ENSG00000108106.12** | *UBE2S* | 0.62 | 2.87 | 2.19 | 2.69 | 0.0069 | protein coding |
| **ENSG00000171241.7** | *SHCBP1* | 0.61 | 0.92 | 0.62 | 0.97 | 0.0073 | protein coding |
| **ENSG00000138092.9** | *CENPO* | 0.61 | 1.15 | 0.88 | 1.18 | 0.0074 | protein coding |
| **ENSG00000168306.11** | *ACOX2* | 0.61 | 4.49 | 4.61 | 3.99 | 0.0100 | protein coding |
| **ENSG00000235587.2** | *GAPDHP65* | 0.61 | 0.70 | 0.39 | 0.60 | 0.0075 | processed pseudogene |
| **ENSG00000130821.14** | *SLC6A8* | 0.61 | 2.82 | 1.46 | 2.28 | 0.0067 | protein coding |
| **ENSG00000173692.11** | *PSMD1* | 0.61 | 4.34 | 4.03 | 4.26 | 0.0079 | protein coding |
| **ENSG00000169016.15** | *E2F6* | 0.61 | 1.56 | 1.33 | 1.56 | 0.0074 | protein coding |
| **ENSG00000094804.8** | *CDC6* | 0.61 | 1.76 | 1.35 | 1.84 | 0.0074 | protein coding |
| **ENSG00000180210.13** | *F2* | 0.61 | 8.83 | 8.94 | 8.11 | 0.0088 | protein coding |
| **ENSG00000149658.16** | *YTHDF1* | 0.61 | 3.89 | 3.84 | 4.07 | 0.0084 | protein coding |
| **ENSG00000057593.12** | *F7* | 0.61 | 4.41 | 5.31 | 4.66 | 0.0100 | protein coding |
| **ENSG00000110169.9** | *HPX* | 0.61 | 7.71 | 8.89 | 7.89 | 0.0069 | protein coding |
| **ENSG00000101323.4** | *HAO1* | 0.61 | 5.70 | 6.42 | 5.53 | 0.0074 | protein coding |
| **ENSG00000213326.4** | *RPS7P11* | 0.61 | 1.19 | 0.95 | 1.21 | 0.0100 | processed pseudogene |
| **ENSG00000076716.8** | *GPC4* | 0.61 | 1.70 | 0.66 | 1.20 | 0.0100 | protein coding |
| **ENSG00000156261.11** | *CCT8* | 0.60 | 4.85 | 4.85 | 5.15 | 0.0069 | protein coding |
| **ENSG00000125970.10** | *RALY* | 0.60 | 3.56 | 3.68 | 4.01 | 0.0088 | protein coding |
| **ENSG00000121957.11** | *GPSM2* | 0.60 | 0.82 | 0.55 | 0.78 | 0.0100 | protein coding |
| **ENSG00000213867.4** | *RP11-829H16.2* | 0.60 | 0.10 | 0.03 | 0.08 | 0.0100 | processed pseudogene |
| **ENSG00000174672.14** | *BRSK2* | 0.60 | 0.10 | 0.08 | 0.24 | 0.0084 | protein coding |
| **ENSG00000214391.3** | *TUBAP2* | 0.60 | 0.20 | 0.12 | 0.21 | 0.0074 | processed pseudogene |
| **ENSG00000119185.11** | *ITGB1BP1* | 0.60 | 2.29 | 2.08 | 2.38 | 0.0069 | protein coding |

**Table D. Functional importance of 50 signatures (LS-RNA-AUC and LS-RNA-WEKA) and their implication in liver cancer or other malignancies.**

| **RNA transcript** | **Type of transcript** | **Regulation in late stage** | **GO terms associated with Gene** | **Function** | **Reported in Previous Literature** | **Reported in case of**  **Liver cancer** |
| --- | --- | --- | --- | --- | --- | --- |
| **LS-RNA-AUC** | | | | | | |
| *NCAPH* | Protein Coding | Overexpressed in Late stage | ATPase activator activity (GO:0001671); mitotic chromosome condensation (GO:0007076); chromosome condensation (GO:0030261) | It encodes Non-SMC condensin I complex subunit H, condensin complex required for conversion of interphase chromatin into mitotic-like condense chromosomes ^1,2^. | YES^2,3^ | NO |
| *CYP4A22* | Protein Coding | Underexpressed in Late stage | oxidoreductase activity, acting on the CH-OH group of donors, NAD or NADP as acceptor (GO:0016616); lipid hydroxylation (GO:0002933); lipid modification (GO:0030258) | It belongs to a large family of cytochrome P450 genes that encode heme-binding monooxygenases. Mainly involved metabolism of drugs and various types of lipids like steroids, cholesterol, vitamin D3, and eicosanoids etc ^1,4–6^ . | NO | NO |
| *HSD17B6* | Protein Coding | Underexpressed in Late stage | androgen biosynthetic process (GO:0006702); steroid catabolic process (GO:0006706); androgen metabolic process (GO:0008209); steroid biosynthetic process (GO:0006694) | It encodes Hydroxysteroid 17-beta dehydrogenase 6, a NAD-dependent oxidoreductase having broad substrate specificity. It has 17-beta-hydroxysteroid dehydrogenase activity towards various steroids ^1,7,8^. | YES ^9^ | NO |
| *GLYATL1* | Protein Coding | Underexpressed in Late stage | N-acyltransferase activity (GO:0016410); glutamine metabolic process (GO:0006541); glutamine family amino acid metabolic process (GO:0009064) | It encodes Glycine N-acyltransferase-like protein 1, which transfers an acyl group to the N-terminus of glutamine ^1^. | YES | Yes^10^ |
| *FTCD* | Protein Coding | Underexpressed in Late stage | hydroxymethyl-, formyl- and related transferase activity (GO:0016742); microtubule binding (GO:0008017); endoplasmic reticulum-Golgi intermediate compartment (GO:0005793); Golgi membrane (GO:0000139); folic acid-containing compound metabolic process (GO:0006760); coenzyme metabolic process (GO:0006732); carboxylic acid metabolic process (GO:0019752); histidine metabolic process (GO:0006547); imidazole-containing compound catabolic process (GO:0052805); alpha-amino acid catabolic process (GO:1901606) | It encodes Formiminotransferase cyclodeaminase; Folate-dependent enzyme, that displays both transferase and deaminase activity. Serves to channel one-carbon units from formiminoglutamate to the folate pool ^1^. | YES | YES ^11^ |
| *HILPDA* | Protein Coding | Overexpressed in Late stage | lipid droplet (GO:0005811); lipid particle organization (GO:0034389); positive regulation of lipid storage (GO:0010884); positive regulation of lipid localization (GO:1905954); regulation of cytokine production (GO:0001817); cellular response to hypoxia (GO:0071456); positive regulation of cytokine production (GO:0001819); positive regulation of cell proliferation (GO:0008284) | It encodes Hypoxia-inducible lipid droplet-associated protein. It increases intracellular lipid accumulation and stimulates expression of various cytokines like IL6, MIF and VEGFA. Further it also promotes cell growth and proliferation ^12,13^. | YES^13^ | NO |
| *CYP2B6* | Protein Coding | Underexpressed in Late stage | arachidonic acid monooxygenase activity (GO:0008391); steroid hydroxylase activity (GO:0008395); heme binding (GO:0020037); steroid hydroxylase activity (GO:0008395); epoxygenase P450 pathway (GO:0019373); drug catabolic process (GO:0042737); steroid metabolic process (GO:0008202) | It encodes Cytochrome P450 2B6, a group of heme-thiolate monooxygenases. It is involved in an NADPH-dependent electron transport pathway. It metabolize various substances like steroids, fatty acids, and xenobiotics ^1,14–16^. | NO | NO |
| *LINC01146* | lincRNA | Underexpressed in Late stage | NA | NA | YES | YES ^17,18^ |
| *CANT1* | Protein Coding | Overexpressed in Late stage | nucleoside-diphosphatase activity (GO:0017110); protein homodimerization activity (GO:0042803); metal ion binding (GO:0046872); specific granule lumen (GO:0035580); glycoprotein biosynthetic process (GO:0009101); secretory granule lumen (GO:0034774)ficolin-1-rich granule lumen (GO:1904813); proteoglycan metabolic process; (GO:0006029); neutrophil degranulation (GO:0043312); neutrophil activation involved in immune response (GO:0002283) | It encodes calcium-dependent nucleotidase1 having a preference for UDP. The order of activity with different substrates is UDP > GDP > UTP > GTP. It is involved in proteoglycan synthesis ^1,19–21^. | YES^22^ | NO |
| *HN1* | Protein Coding | Overexpressed in Late stage | NA | It encodes Hematological and neurological expressed 1 protein or Jupiter microtubule associated homolog 1. It modulates negatively AKT-mediated GSK3B signaling (21323578, 22155408) It plays a role in the regulation of cell cycle and cell adhesion ^1,23,24^. | YES^19^ | YES ^19^ |
| *ALDOA* | protein_coding | Overexpressed in Late stage | aldehyde-lyase activity (GO:0016832); actin binding (GO:0003779); cadherin binding (GO:0045296); tubulin binding (GO:0015631); RNA binding (GO:0003723); cytoskeleton (GO:0005856); ficolin-1-rich granule lumen (GO:1904813); secretory granule lumen (GO:0034774); fructose metabolic process (GO:0006000); purine ribonucleoside triphosphate biosynthetic process (GO:0009206); glycolytic process through glucose-6-phosphate (GO:0061620); carbohydrate catabolic process (GO:0016052); glycolytic process (GO:0006096); gluconeogenesis (GO:0006094); purine ribonucleotide biosynthetic process (GO:0009152) | It encodes a member of the class I fructose-bisphosphate aldolase protein family. The encoded protein is a glycolytic enzyme that catalyzes the reversible conversion of fructose-1,6-bisphosphate to glyceraldehyde 3-phosphate and dihydroxyacetone phosphate.  It plays a key role in glycolysis and gluconeogenesis ^1^. | YES | YES^25,26^ |
| *SPP2* | Protein Coding | Underexpressed in Late stage | NA | It encodes a secreted phosphoprotein 24, a member of the cystatin superfamily. It may coordinate an aspect of bone turnover ^1^. | NO | NO |
| *CNFN* | Protein Coding | Overexpressed in Late stage | NA | It encodes Cornifelin, the insoluble cornified cell envelope (CE) of stratified squamous epithelia ^1,27^ . | NO | NO |
| *BDH1* | Protein Coding | Underexpressed in Late stage | oxidoreductase activity, acting on the CH-OH group of donors, NAD or NADP as acceptor (GO:0016616); mitochondrial matrix (GO:0005759); mitochondrion (GO:0005739); ketone body metabolic process (GO:1902224); fatty acid derivative biosynthetic process (GO:1901570) | It encodes D-beta-hydroxybutyrate dehydrogenase, a homotetrameric lipid-requiring enzyme of the mitochondrial membrane. It catalyzes the interconversion of acetoacetate and (R)-3-hydroxybutyrate, the two major ketone bodies produced during fatty acid catabolism ^1,27^. | NO | NO |
| *SLC2A2* | Protein Coding | Underexpressed in Late stage | hexose transmembrane transporter activity (GO:0015149); glucose transmembrane transporter activity (GO:0005355); integral component of plasma membrane (GO:0005887); intestinal absorption (GO:0050892); monosaccharide transport (GO:0015749) ; regulation of peptide hormone secretion (GO:0090276) | It encodes GLUT2, Facilitative glucose transporter. It stimulates the bidirectional transfer of glucose across the plasma membrane of hepatocytes and is responsible for uptake of glucose by the beta cells ^1^. | YES | YES^26,28^ |
| *ITIH1* | Protein Coding | Underexpressed in Late stage | metal ion binding (GO:0046872); calcium ion binding (GO:0005509) | It encodes inter-alpha-trypsin inhibitor-1 family of proteins, a preproprotein which is proteolytically processed to generate the heavy chain of the inter-alpha-trypsin inhibitor complex, secreted by hepatocytes into the blood. The heavy chain further interacts with hyaluronan, which may play a role in ovulation and fertilization ^1^. | YES | YES^29^ |
| *A1BG* | Protein Coding | Underexpressed in Late stage | platelet alpha granule lumen (GO:0031093); cytoplasmic vesicle lumen (GO:0060205); regulated exocytosis (GO:0045055); platelet degranulation (GO:0002576) | It encodes Alpha-1B-glycoprotein. This protein shows sequence similarity to the variable regions of some immunoglobulin supergene family member proteins ^1^. | YES^30^ | NO |
| *EFNA3* | Protein Coding | Overexpressed in Late stage | ephrin receptor activity (GO:0005003); ephrin receptor binding (GO:0046875); integral component of plasma membrane (GO:0005887); positive regulation of aspartic-type endopeptidase activity involved in amyloid precursor protein catabolic process (GO:1902961); positive regulation of amyloid precursor protein catabolic process (GO:1902993); positive regulation of endopeptidase activity (GO:0010950); negative regulation of angiogenesis (GO:0016525); ephrin receptor signaling pathway (GO:0048013); axonogenesis (GO:0007409) | It encodes Ephrin-A3. It belongs to receptor protein-tyrosine kinases and have been implicated in mediating developmental events, especially in the nervous system and in erythropoiesis ^1^. | YES | YES^31^ |
| *B3GNTL1* | Protein Coding | Overexpressed in Late stage | NA | It encodes UDP-GlcNAc:BetaGal Beta-1,3-N-Acetylglucosaminyltransferase Like 1. Gene Ontology (GO) annotations related to this gene include transferase activity, transferring glycosyl groups ^1^. | YES^32^ | NO |
| *UROC1* | Protein Coding | Underexpressed in Late stage | hydro-lyase activity (GO:0016836); histidine metabolic process (GO:0006547); imidazole-containing compound catabolic process (GO:0052805); alpha-amino acid catabolic process (GO:1901606) | This gene encodes Urocanate hydratase which involved in histidine catabolism, metabolizing urocanic acid to formiminoglutamic acid ^1^. | NO | NO |
| **LS-RNA-WEKA** | | | | | | |
| *MAT1A* | Protein Coding | Underexpressed in Late stage | sulfur amino acid catabolic process (GO:0000098); methionine metabolic process (GO:0006555); aspartate family amino acid catabolic process (GO:0009068); sulfur amino acid metabolic process (GO:0000096); coenzyme biosynthetic process (GO:0009108); protein homooligomerization (GO:0051260) | It encodes methionine adenosyltransferase I, alpha; Catalyzes the formation of S-adenosylmethionine from methionine and ATP ^33,34^. | YES | YES^35^ |
| *DCK* | Protein Coding | Overexpressed in Late stage | protein homodimerization activity (GO:0042803); pyrimidine-containing compound metabolic process (GO:0072527); pyrimidine nucleoside salvage (GO:0043097); purine-containing compound salvage (GO:0043101); nucleoside salvage (GO:0043174); pyrimidine nucleoside biosynthetic process (GO:0046134); purine-containing compound biosynthetic process (GO:0072522) | It encodes Deoxycytidine kinase, required for the phosphorylation of the deoxyribonucleosides deoxycytidine (dC), deoxyguanosine (dG) and deoxyadenosine (dA) ^1,36,37^. | YES | YES^38^ |
| *CDCA5* | Protein Coding | Overexpressed in Late stage | chromosome, centromeric region (GO:0000775); chromosomal region (GO:0098687); nuclear chromatin (GO:0000790); nuclear chromosome part (GO:0044454); regulation of sister chromatid cohesion (GO:0007063); positive regulation of mitotic cell cycle phase transition (GO:1901992); mitotic metaphase plate congression (GO:0007080); positive regulation of mitotic nuclear division (GO:0045840); regulation of DNA binding (GO:0051101); DNA repair (GO:0006281); mitotic sister chromatid segregation (GO:0000070) | It encodes Sororin, a regulator of sister chromatid cohesion in mitosis stabilizing cohesin complex association with chromatin. Cohesion ensures that chromosome partitioning in correct manner in both meiotic and mitotic cells and plays an important role in DNA repair such as efficient DNA double-stranded break repair. It is enriched in mitotic sister chromatid cohesion ^1,39,40^. | YES | YES^41^ |
| *RPP25* | Protein Coding | Overexpressed in Late stage | ribonuclease P activity (GO:0004526); tRNA-specific ribonuclease activity (GO:0004549); ribonuclease P complex (GO:0030677); multimeric ribonuclease P complex (GO:0030681); microtubule cytoskeleton (GO:0015630); microtubule organizing center (GO:0005815); tRNA 5'-leader removal (GO:0001682); ribosome biogenesis (GO:0042254); rRNA metabolic process (GO:0016072); ncRNA processing (GO:0034470) | It might be involved in transcriptional regulation ^41^. | NO | NO |
| *GPR37L1* | Protein Coding | Overexpressed in Late stage | G-protein coupled peptide receptor activity (GO:0008528); negative regulation of response to reactive oxygen species (GO:1901032); regulation of hydrogen peroxide-induced cell death (GO:1903205); negative regulation of hydrogen peroxide-induced cell death (GO:1903206); adenylate cyclase-inhibiting G-protein coupled receptor signaling pathway (GO:0007193); regulation of MAPK cascade (GO:0043408) | Receptor for the neuroprotective and glioprotective factor prosaposin ^42,43^. Ligand binding induces endocytosis, followed by an ERK phosphorylation cascade. Negative regulate the hydrogen peroxide-induced cell death (GO:1903206), opioid receptor activity (GO:0004985), oxytocin receptor activity (GO:0004990) ^42,43^. | NO | NO |
| *FUT11* | Protein Coding | Overexpressed in Late stage | fucosyltransferase activity (GO:0008417); Golgi membrane (GO:0000139); Golgi subcompartment (GO:0098791); | It encodes Fucosyltransferase 11 (alpha (1,3) fucosyltransferase); a protein involved in the pathway protein glycosylation ^1^. | NO | NO |
| *NECAB1* | Protein Coding | Overexpressed in Late stage | regulation of glycoprotein biosynthetic process (GO:0010559); regulation of amyloid precursor protein biosynthetic process (GO:0042984) | It encodes N-terminal EF-hand calcium binding protein 1 ^1^. | NO | NO |
| *LCAT* | Protein Coding | Underexpressed in Late stage | O-acyltransferase activity (GO:0008374); organonitrogen compound biosynthetic process (GO:1901566) | It encodes Lecithin-cholesterol acyltransferase; central enzyme in the extracellular metabolism of plasma lipoproteins. It converts cholesterol and phosphatidylcholines (lecithins) to cholesteryl esters and lysophosphatidylcholines on the surface of high and low density lipoproteins (HDLs and LDLs) ^44–46^. | YES | YES ^47^ |
| *RAMP3* | Protein Coding | Underexpressed in Late stage | protein transporter activity (GO:0008565); integral component of plasma membrane (GO:0005887); positive regulation of protein kinase A signaling (GO:0010739); positive regulation of calcium ion import (GO:0090280); dimeric G-protein coupled receptor signaling pathway (GO:0038042); calcitonin family receptor signaling pathway (GO:0097646); amylin receptor signaling pathway (GO:0097647); positive regulation of receptor recycling (GO:0001921); regulation of cAMP metabolic process (GO:0030814); regulation of protein kinase A signaling (GO:0010738); positive regulation of cell death (GO:0010942) | Receptor (G protein-coupled) activity modifying protein 3; Plays a role in cardioprotection by reducing cardiac hypertrophy and perivascular fibrosis in a GPER1-dependent manner ^48,49^ . | YES | YES^48,49^ |
| *CFHR3* | Protein Coding | Underexpressed in Late stage | NA | It encodes Complement factor H-related 3; Might be involved in complement regulation ^48,49^. | NO | NO |
| *FNTB* | Protein Coding | Overexpressed in Late stage | prenyltransferase activity (GO:0004659); transition metal ion binding (GO:0046914); protein prenylation (GO:0018342); regulation of rhodopsin mediated signaling pathway (GO:0022400); regulation of G-protein coupled receptor protein signaling pathway (GO:0008277); regulation of response to external stimulus (GO:0032101); cellular protein modification process (GO:0006464) | It encodes Beta subunit of the farnesyltransferase complex. It catalyzes the transfer of a farnesyl moiety from farnesyl diphosphate to a cysteine at the fourth position from the C-terminus of several proteins having the C-terminal sequence Cys-aliphatic-aliphatic-X ^48,49^. | NO | NO |
| *ZNF576* | Protein Coding | Overexpressed in Late stage | NA | It might be involved in transcriptional regulation ^50^. | YES^51^ | NO |
| *NETO2* | Protein Coding | Overexpressed in Late stage | NA | It encodes Neuropilin And Tolloid Like 2. This protein has two extracellular CUB domains followed by a low-density lipoprotein class A (LDLa) domain. It slows the degradation of kainate receptor-mediated excitatory postsynaptic currents (EPSCs), therefore affect the synaptic transmission ^1^ (29425356). | YES^52^ | NO |
| *SLC22A10* | Protein Coding | Underexpressed in Late stage | salt transmembrane transporter activity (GO:1901702); urate transmembrane transporter activity (GO:0015143); organic acid transmembrane transporter activity (GO:0005342); inorganic anion exchanger activity (GO:0005452); secondary active transmembrane transporter activity (GO:0015291); sodium-independent organic anion transmembrane transporter activity (GO:0015347); organic anion transmembrane transporter activity (GO:0008514); integral component of plasma membrane (GO:0005887); urate transport (GO:0015747); sodium-independent organic anion transport (GO:0043252); organic substance transport (GO:0071702); transmembrane transport (GO:0055085) | It encodes Solute carrier family 22, member 10 encodes organic anion transporter -5 ^52^(11327718), it acts as kidney injury biomarker ^52^(26230185). | NO | NO |
| *ELOVL3* | Protein Coding | Overexpressed in Late stage | fatty acid elongase activity (GO:0009922); fatty acid synthase activity (GO:0004312); integral component of endoplasmic reticulum membrane (GO:0030176); fatty acid elongation (GO:0030497); linoleic acid metabolic process (GO:0043651); sphingolipid metabolic process (GO:0006665); organonitrogen compound biosynthetic process (GO:1901566) | It encodes Elongation of very long chain fatty acids protein 3, fatty acid elongase 3. It catalyzes the first and rate-limiting reaction of the four that constitute the long-chain fatty acids elongation cycle ^1^ . | NO | NO |
| GAPDHP63 | processed_pseudogene | Overexpressed in Late stage | NA | NA | NO | NO |
| PTMAP2 | processed_pseudogene | Overexpressed in Late stage | NA | NA | NO | NO |
| RP11-829H16.2 | processed_pseudogene | Overexpressed in Late stage | NA | NA | NO | NO |
| AC018712.2 | processed_pseudogene | Overexpressed in Late stage | NA | NA | NO | NO |
| RP3-375P9.2 | processed_pseudogene | Overexpressed in Late stage | NA | NA | NO | NO |
| HNRNPA1P37 | processed_pseudogene | Overexpressed in Late stage | NA | NA | NO | NO |
| RP11-473P24.2 | processed_pseudogene | Overexpressed in Late stage | NA | NA | NO | NO |
| CALM2P4 | processed_pseudogene | Overexpressed in Late stage | NA | NA | NO | NO |
| RPS3P7 | processed_pseudogene | Overexpressed in Late stage | NA | NA | NO | NO |
| RP11-501M7.1 | unprocessed_pseudogene | Overexpressed in Late stage | NA | NA | NO | NO |
| DDX39BP2 | unprocessed_pseudogene | Overexpressed in Late stage | NA | NA | NO | NO |
| AP000279.1 | miRNA | Overexpressed in Late stage | NA | NA | NO | NO |
| FABP5P3 | transcribed_processed_pseudogene | Overexpressed in Late stage | NA | NA | NO | NO |
| RP11-646I6.5 | lincRNA | Overexpressed in Late stage | NA | NA | NO | NO |
| AC007278.3 | sense_intronic | Underexpressed in Late stage | NA | NA | NO | NO |

**Table E. 30 RNA transcripts (LS-RNA-WEKA) selected by WEKA for stage classification.**

| **Ensemble_id** | **Mean in Early stage (Log2(FPKM))** | **Mean in late stage-l (Log2(FPKM))** | **Mean Difference** | **FDR (False discovery rate)** | **Gene symbol** | **Class** |
| --- | --- | --- | --- | --- | --- | --- |
| **ENSG00000151224.11** | 7.55 | 6.86 | 0.69 | 0.02 | *MAT1A* | protein coding |
| **ENSG00000156136.8** | 1.88 | 2.16 | -0.28 | 0.04 | *DCK* | protein coding |
| **ENSG00000146670.8** | 1.60 | 2.00 | -0.40 | 0.03 | *CDCA5* | protein coding |
| **ENSG00000178718.6** | 1.30 | 1.76 | -0.46 | 0.02 | *RPP25* | protein coding |
| **ENSG00000170075.8** | 0.09 | 0.16 | -0.07 | 0.03 | *GPR37L1* | protein coding |
| **ENSG00000196968.9** | 1.21 | 1.40 | -0.19 | 0.04 | *FUT11* | protein coding |
| **ENSG00000123119.10** | 0.15 | 0.29 | -0.14 | 0.02 | *NECAB1* | protein coding |
| **ENSG00000213398.6** | 3.89 | 3.21 | 0.68 | 0.01 | *LCAT* | protein coding |
| **ENSG00000122679.7** | 3.23 | 2.70 | 0.53 | 0.01 | *RAMP3* | protein coding |
| **ENSG00000116785.12** | 3.93 | 3.03 | 0.91 | 0.03 | *CFHR3* | protein coding |
| **ENSG00000257365.6** | 1.44 | 1.58 | -0.14 | 0.03 | *FNTB* | protein coding |
| **ENSG00000124444.14** | 2.01 | 2.17 | -0.16 | 0.03 | *ZNF576* | protein coding |
| **ENSG00000171208.8** | 0.34 | 0.57 | -0.23 | 0.01 | *NETO2* | protein coding |
| **ENSG00000184999.10** | 2.48 | 1.78 | 0.70 | 0.01 | *SLC22A10* | protein coding |
| **ENSG00000119915.4** | 0.14 | 0.32 | -0.18 | 0.02 | *ELOVL3* | protein coding |
| **ENSG00000218582.2** | 0.19 | 0.30 | -0.11 | 0.01 | GAPDHP63 | processed pseudogene |
| **ENSG00000197744.5** | 1.01 | 1.29 | -0.28 | 0.02 | PTMAP2 | processed pseudogene |
| **ENSG00000213867.4** | 0.03 | 0.08 | -0.04 | 0.01 | RP11-829H16.2 | processed pseudogene |
| **ENSG00000230104.1** | 0.05 | 0.15 | -0.10 | 0.04 | AC018712.2 | processed pseudogene |
| **ENSG00000217130.1** | 0.48 | 0.70 | -0.22 | 0.03 | RP3-375P9.2 | processed pseudogene |
| **ENSG00000218574.1** | 0.01 | 0.02 | -0.01 | 0.02 | HNRNPA1P37 | processed pseudogene |
| **ENSG00000243977.1** | 0.01 | 0.03 | -0.02 | 0.03 | RP11-473P24.2 | processed pseudogene |
| **ENSG00000233662.1** | 0.01 | 0.02 | -0.01 | 0.03 | CALM2P4 | processed pseudogene |
| **ENSG00000243101.1** | 0.02 | 0.03 | -0.01 | 0.05 | RPS3P7 | processed pseudogene |
| **ENSG00000248374.1** | 0.00 | 0.01 | -0.01 | 0.04 | RP11-501M7.1 | unprocessed pseudogene |
| **ENSG00000238024.1** | 0.07 | 0.22 | -0.15 | 0.01 | DDX39BP2 | unprocessed pseudogene |
| **ENSG00000273662.1** | 0.09 | 0.21 | -0.12 | 0.03 | AP000279.1 | miRNA |
| **ENSG00000241735.1** | 0.02 | 0.03 | -0.01 | 0.03 | FABP5P3 | transcribed_processed pseudogene |
| **ENSG00000269921.1** | 0.15 | 0.22 | -0.06 | 0.03 | RP11-646I6.5 | lincRNA |
| **ENSG00000234389.1** | 0.07 | 0.03 | 0.03 | 0.05 | AC007278.3 | sense_intronic |

**Table F. The Performance of models using 100 RNA transcripts or features. These features selected by F-ANOVA method.**

| **Technique** | **Dataset** | **Performance Measures** | | | | |
| --- | --- | --- | --- | --- | --- | --- |
|  |  | **Sensitivity (%)** | **Specificity (%)** | **Accuracy (%)** | **MCC** | **AUC** |
| **SVM** | Training | 73.91 | 68.79 | 71.33 | 0.43 | 0.75 (0.69-0.80) |
|  | Validation | 71.43 | 69.44 | 70.42 | 0.41 | 0.74 (0.68-0.79) |
| **Random Forest** | Training | 71.01 | 62.41 | 66.67 | 0.34 | 0.72 (0.62-0.82) |
|  | Validation | 68.57 | 61.11 | 64.79 | 0.3 | 0.69 (0.64-0.75) |
| **Naïve bayes** | Training | 82.61 | 57.45 | 69.89 | 0.41 | 0.72 (0.60-0.84) |
|  | Validation | 80 | 55.56 | 67.61 | 0.37 | 0.69 (0.67-0.73) |
| **SMO** | Training | 76.81 | 66.67 | 71.68 | 0.44 | 0.72 (0.66-0.78) |
|  | Validation | 74.29 | 63.89 | 69.01 | 0.38 | 0.69 (0.64-0.75) |
| **J48** | Training | 67.39 | 64.54 | 65.95 | 0.32 | 0.66(0.55-0.78) |
|  | Validation | 57.14 | 69.44 | 63.38 | 0.27 | 0.62 (0.56-0.68) |

**Table G. The performance of stage classification hybrid models developed using 51 features (LS-CpG-RNA-hybrid) that comprise 21 CpG sites and 30 RNA transcripts on full dataset.**

| **Machine Learning Techniques** | **Dataset** | **Performance Measures** | | | | |
| --- | --- | --- | --- | --- | --- | --- |
|  |  | **Sensitivity (%)** | **Specificity (%)** | **Accuracy (%)** | **MCC** | **AUC with CI** |
| SVM | Full dataset | 82.08 | 75.71 | 78.86 | 0.58 | 0.83 (0.78-0.88) |
| Random Forest | Full dataset | 78.03 | 75.14 | 76.57 | 0.53 | 0.84 (0.79-0.90) |
| Naïve bayes | Full dataset | 85.55 | 70.62 | 78.00 | 0.57 | 0.81 (0.76-0.86) |
| SMO | Full dataset | 82.08 | 72.88 | 77.43 | 0.55 | 0.78 (0.73-0.83) |
| J48 | Full dataset | 63.58 | 74.58 | 69.14 | 0.38 | 0.75 (0.69-0.89) |

**Table H. The Performance of models using 38 features (15CpG + 23mRNA). These features selected by WEKA from 1740 features (1293 RNA transcripts+ 447CpG).**

| **Technique** | **Dataset** | **Performance Measures** | | | | |
| --- | --- | --- | --- | --- | --- | --- |
|  |  | **Sensitivity** | **Specificity** | **Accuracy (%)** | **MCC** | **AUC** |
| SVM | Training | 76.09 | 78.01 | 77.06 | 0.54 | 0.82 |
|  | Validation | 71.43 | 77.78 | 74.65 | 0.49 | 0.8 |
| Random Forest | Training | 78.99 | 71.63 | 75.27 | 0.51 | 0.85 |
|  | Validation | 65.71 | 63.89 | 64.79 | 0.3 | 0.74 |
| Naïve bayes | Training | 84.06 | 65.96 | 74.91 | 0.51 | 0.81 |
|  | Validation | 82.86 | 63.89 | 73.24 | 0.48 | 0.75 |
| SMO | Training | 77.54 | 77.3 | 77.42 | 0.55 | 0.77 |
|  | Validation | 71.43 | 77.78 | 74.65 | 0.49 | 0.75 |
| J48 | Training | 71.74 | 69.5 | 70.61 | 0.41 | 0.72 |
|  | Validation | 48.57 | 63.89 | 56.34 | 0.13 | 0.62 |

**Table I. Top 496 CpG sites (LCN-CPG-AUC) having AUC (= or > 0.9) based on single gene threshold based approach for cancer versus normal classification.**

| **Probe ID (CpG site)** | **AUC** | **Threshold (Beta value)** | **Mean in Cancer (Beta value)** | **Mean in Normal (Beta value)** | **Gene Symbol** | **Bonferroni adjusted p-value** | **Feature type** | **Regulation** |
| --- | --- | --- | --- | --- | --- | --- | --- | --- |
| **cg07274716** | 0.97 | 0.35 | 0.65 | 0.23 | *PITX1* | 4.509633e-66 | Island | Hypermethylated |
| **cg08970694** | 0.96 | 0.66 | 0.37 | 0.77 | *HBE1; HBG2* | 1.425378e-68 | . | Hypomethylated |
| **cg03214622** | 0.96 | 0.67 | 0.38 | 0.79 | *RBFOX3* | 4.228485e-70 | . | Hypomethylated |
| **cg06353345** | 0.96 | 0.64 | 0.31 | 0.75 | *HBE1; HBG2;OR51B4* | 1.118035e-73 | . | Hypomethylated |
| **cg09170112** | 0.96 | 0.63 | 0.26 | 0.83 | *.* | 4.024592e-57 | . | Hypomethylated |
| **cg24035245** | 0.96 | 0.44 | 0.70 | 0.27 | *.* | 3.94278e-56 | Island | Hypermethylated |
| **cg18161025** | 0.95 | 0.74 | 0.41 | 0.84 | *BRINP2* | 4.624959e-86 | S_Shelf | Hypomethylated |
| **cg12176793** | 0.95 | 0.53 | 0.27 | 0.72 | *RP11-37B2.1* | 2.95557e-77 | . | Hypomethylated |
| **cg18766755** | 0.95 | 0.55 | 0.27 | 0.72 | *SPRR2A* | 3.794984e-63 | . | Hypomethylated |
| **cg00378950** | 0.95 | 0.63 | 0.31 | 0.78 | *TGIF2LX* | 2.799877e-59 | . | Hypomethylated |
| **cg26151206** | 0.95 | 0.64 | 0.32 | 0.82 | *OR10K2* | 6.996451e-82 | . | Hypomethylated |
| **cg18099070** | 0.95 | 0.73 | 0.31 | 0.84 | *PTPRN2* | 1.881568e-62 | . | Hypomethylated |
| **cg16657538** | 0.95 | 0.32 | 0.63 | 0.11 | *RP11-158H5.8;ZSCAN30* | 1.592715e-70 | Island | Hypermethylated |
| **cg20172627** | 0.95 | 0.12 | 0.59 | 0.04 | *.* | 5.8249e-109 | S_Shore | Hypermethylated |
| **cg16888547** | 0.94 | 0.75 | 0.43 | 0.84 | *PDE1C* | 5.913109e-74 | S_Shore | Hypomethylated |
| **cg24659758** | 0.94 | 0.70 | 0.46 | 0.86 | *AIFM2;AIFM2* | 7.233026e-77 | N_Shore | Hypomethylated |
| **cg01262413** | 0.94 | 0.55 | 0.30 | 0.70 | *PTPRN2* | 1.729383e-38 | S_Shore | Hypomethylated |
| **cg26765743** | 0.94 | 0.64 | 0.36 | 0.77 | *MIR646HG* | 2.859652e-62 | . | Hypomethylated |
| **cg16151261** | 0.94 | 0.53 | 0.29 | 0.70 | *LINC01029* | 4.077964e-57 | S_Shore | Hypomethylated |
| **cg02567289** | 0.94 | 0.53 | 0.28 | 0.69 | *RP11-634B7.4;RP11-978I15.10* | 6.072686e-42 | S_Shelf | Hypomethylated |
| **cg14649589** | 0.94 | 0.55 | 0.29 | 0.71 | *.* | 1.324619e-49 | . | Hypomethylated |
| **cg01379207** | 0.94 | 0.90 | 0.52 | 0.94 | *RP11-676M6.1* | 6.312196e-88 | . | Hypomethylated |
| **cg07804289** | 0.94 | 0.44 | 0.24 | 0.66 | *.* | 1.325347e-44 | . | Hypomethylated |
| **cg04604142** | 0.94 | 0.44 | 0.26 | 0.68 | *LUC7L3* | 1.64703e-32 | N_Shore | Hypomethylated |
| **cg23962746** | 0.94 | 0.53 | 0.25 | 0.67 | *.* | 9.908313e-37 | . | Hypomethylated |
| **cg10948284** | 0.94 | 0.75 | 0.41 | 0.84 | *RP11-706C16.8* | 7.379768e-63 | S_Shelf | Hypomethylated |
| **cg05353872** | 0.94 | 0.52 | 0.24 | 0.67 | *OR2M3* | 3.174456e-54 | . | Hypomethylated |
| **cg08948808** | 0.94 | 0.52 | 0.24 | 0.68 | *SPRR4* | 1.799958e-46 | . | Hypomethylated |
| **cg15727252** | 0.94 | 0.85 | 0.48 | 0.91 | *SYT11* | 1.507537e-85 | S_Shore | Hypomethylated |
| **cg17514199** | 0.94 | 0.42 | 0.22 | 0.66 | *RP11-193H5.1;ZP4;ZP4* | 3.407145e-41 | . | Hypomethylated |
| **cg07309576** | 0.94 | 0.86 | 0.47 | 0.91 | *AC104389.28; HBE1; HBG2;OR51I1* | 3.345614e-83 | . | Hypomethylated |
| **cg13320538** | 0.94 | 0.76 | 0.45 | 0.89 | *.* | 4.499317e-91 | S_Shore | Hypomethylated |
| **cg02442572** | 0.94 | 0.76 | 0.41 | 0.87 | *.* | 2.836118e-99 | . | Hypomethylated |
| **cg25416582** | 0.94 | 0.74 | 0.36 | 0.83 | *.* | 3.221805e-78 | . | Hypomethylated |
| **cg10222925** | 0.94 | 0.63 | 0.28 | 0.75 | *DCAF4L2* | 1.319074e-76 | N_Shore | Hypomethylated |
| **cg14128411** | 0.94 | 0.64 | 0.33 | 0.82 | *MYT1L;MYT1L;MYT1L* | 3.948934e-68 | . | Hypomethylated |
| **cg07623037** | 0.94 | 0.75 | 0.37 | 0.86 | *.* | 2.418568e-92 | . | Hypomethylated |
| **cg00217080** | 0.94 | 0.53 | 0.28 | 0.83 | *.* | 3.230285e-50 | Island | Hypomethylated |
| **cg24833737** | 0.94 | 0.73 | 0.31 | 0.90 | *.* | 1.386718e-92 | S_Shore | Hypomethylated |
| **cg14486338** | 0.94 | 0.42 | 0.68 | 0.20 | *KCNS2;STK3* | 9.538791e-60 | Island | Hypermethylated |
| **cg22531183** | 0.94 | 0.41 | 0.63 | 0.23 | *CTD-2126E3.3;FLJ26850;ZNF473* | 1.717599e-54 | Island | Hypermethylated |
| **cg13879483** | 0.94 | 0.34 | 0.72 | 0.15 | *USP44* | 2.625032e-91 | Island | Hypermethylated |
| **cg07124687** | 0.94 | 0.43 | 0.70 | 0.21 | *.* | 2.056971e-50 | Island | Hypermethylated |
| **cg11174855** | 0.94 | 0.43 | 0.65 | 0.22 | *NKX6-2;NKX6-2* | 1.800709e-39 | Island | Hypermethylated |
| **cg13481969** | 0.94 | 0.32 | 0.62 | 0.14 | *RP11-535M15.1* | 2.402562e-53 | Island | Hypermethylated |
| **cg18803104** | 0.94 | 0.42 | 0.70 | 0.23 | *.* | 1.913611e-32 | Island | Hypermethylated |
| **cg27364741** | 0.94 | 0.33 | 0.61 | 0.16 | *OTX1* | 4.201068e-76 | Island | Hypermethylated |
| **cg25622366** | 0.94 | 0.21 | 0.57 | 0.06 | *OTX1* | 4.034981e-98 | Island | Hypermethylated |
| **cg08363001** | 0.93 | 0.64 | 0.35 | 0.76 | *.* | 9.436807e-58 | . | Hypomethylated |
| **cg01649219** | 0.93 | 0.79 | 0.47 | 0.87 | *.* | 5.729089e-89 | . | Hypomethylated |
| **cg26327092** | 0.93 | 0.70 | 0.44 | 0.84 | *.* | 2.886143e-60 | . | Hypomethylated |
| **cg01372939** | 0.93 | 0.65 | 0.37 | 0.78 | *.* | 2.702219e-68 | . | Hypomethylated |
| **cg26606257** | 0.93 | 0.65 | 0.37 | 0.77 | *MYT1L* | 7.156035e-71 | . | Hypomethylated |
| **cg17932055** | 0.93 | 0.66 | 0.39 | 0.80 | *.* | 4.203468e-72 | N_Shelf | Hypomethylated |
| **cg25201255** | 0.93 | 0.55 | 0.31 | 0.71 | *NTF3* | 7.321107e-50 | . | Hypomethylated |
| **cg14826683** | 0.93 | 0.53 | 0.28 | 0.69 | *SPRR2D* | 9.340363e-54 | . | Hypomethylated |
| **cg07159802** | 0.93 | 0.64 | 0.35 | 0.76 | *OR1C1* | 5.705332e-69 | . | Hypomethylated |
| **cg10259094** | 0.93 | 0.78 | 0.45 | 0.86 | *GJD2* | 1.716811e-63 | N_Shelf | Hypomethylated |
| **cg21180080** | 0.93 | 0.66 | 0.39 | 0.80 | *.* | 9.112052e-83 | S_Shelf | Hypomethylated |
| **cg04505023** | 0.93 | 0.64 | 0.34 | 0.75 | *.* | 3.138595e-71 | . | Hypomethylated |
| **cg22947965** | 0.93 | 0.68 | 0.39 | 0.80 | *.* | 5.434321e-81 | . | Hypomethylated |
| **cg01011918** | 0.93 | 0.54 | 0.30 | 0.71 | *SPRR2D* | 3.214536e-40 | . | Hypomethylated |
| **cg10818986** | 0.93 | 0.68 | 0.38 | 0.79 | *OR2T8* | 3.714096e-67 | . | Hypomethylated |
| **cg04165859** | 0.93 | 0.75 | 0.42 | 0.84 | *.* | 7.410036e-72 | . | Hypomethylated |
| **cg15193614** | 0.93 | 0.68 | 0.36 | 0.78 | *.* | 4.278891e-63 | . | Hypomethylated |
| **cg20312687** | 0.93 | 0.53 | 0.29 | 0.71 | *DEFB118* | 5.69842e-47 | . | Hypomethylated |
| **cg18805469** | 0.93 | 0.65 | 0.37 | 0.79 | *SPRR4* | 6.074003e-87 | . | Hypomethylated |
| **cg07164722** | 0.93 | 0.57 | 0.35 | 0.77 | *NLRP3* | 7.260667e-54 | . | Hypomethylated |
| **cg11725972** | 0.93 | 0.76 | 0.45 | 0.87 | *RP5-912I13.1* | 1.582458e-80 | . | Hypomethylated |
| **cg21419383** | 0.93 | 0.67 | 0.38 | 0.80 | *IVL* | 4.530671e-68 | . | Hypomethylated |
| **cg04498110** | 0.93 | 0.67 | 0.40 | 0.83 | *CX3CR1* | 2.623394e-80 | . | Hypomethylated |
| **cg20137179** | 0.93 | 0.64 | 0.34 | 0.76 | *.* | 6.656962e-65 | . | Hypomethylated |
| **cg06550539** | 0.93 | 0.65 | 0.42 | 0.85 | *CFAP46* | 8.089229e-56 | Island | Hypomethylated |
| **cg04574507** | 0.93 | 0.56 | 0.29 | 0.72 | *CD1B* | 8.217567e-36 | . | Hypomethylated |
| **cg20855160** | 0.93 | 0.33 | 0.18 | 0.61 | *.* | 1.063332e-17 | . | Hypomethylated |
| **cg13316295** | 0.93 | 0.56 | 0.30 | 0.72 | *AARD* | 7.00322e-40 | S_Shelf | Hypomethylated |
| **cg15897635** | 0.93 | 0.44 | 0.24 | 0.67 | *.* | 6.086124e-30 | N_Shelf | Hypomethylated |
| **cg09938273** | 0.93 | 0.75 | 0.41 | 0.84 | *.* | 7.430597e-81 | . | Hypomethylated |
| **cg08448893** | 0.93 | 0.64 | 0.37 | 0.80 | *.* | 7.004227e-76 | . | Hypomethylated |
| **cg03872376** | 0.93 | 0.64 | 0.28 | 0.72 | *RP11-193H5.1;ZP4;ZP4* | 1.404295e-65 | . | Hypomethylated |
| **cg05623614** | 0.93 | 0.65 | 0.36 | 0.79 | *C18orf42* | 1.422989e-62 | . | Hypomethylated |
| **cg07614747** | 0.93 | 0.44 | 0.22 | 0.66 | *.* | 2.469946e-36 | N_Shore | Hypomethylated |
| **cg27318774** | 0.93 | 0.77 | 0.46 | 0.91 | *.* | 1.574159e-94 | . | Hypomethylated |
| **cg08950105** | 0.93 | 0.64 | 0.36 | 0.81 | *.* | 1.299431e-70 | N_Shore | Hypomethylated |
| **cg24107750** | 0.93 | 0.77 | 0.44 | 0.89 | *PRKCQ* | 1.70039e-93 | . | Hypomethylated |
| **cg11085294** | 0.93 | 0.64 | 0.35 | 0.80 | *ASTN1* | 8.264782e-79 | . | Hypomethylated |
| **cg00830435** | 0.93 | 0.73 | 0.43 | 0.88 | *.* | 6.433167e-71 | Island | Hypomethylated |
| **cg13380622** | 0.93 | 0.63 | 0.34 | 0.80 | *FCRL4* | 3.870208e-73 | . | Hypomethylated |
| **cg23737927** | 0.93 | 0.53 | 0.24 | 0.71 | *.* | 1.02227e-56 | N_Shelf | Hypomethylated |
| **cg08786003** | 0.93 | 0.53 | 0.24 | 0.71 | *FCRL3* | 9.289584e-53 | . | Hypomethylated |
| **cg13077865** | 0.93 | 0.54 | 0.27 | 0.74 | *ZFPM2* | 5.360834e-41 | . | Hypomethylated |
| **cg20785459** | 0.93 | 0.65 | 0.36 | 0.82 | *HBBP1* | 1.998976e-76 | . | Hypomethylated |
| **cg08426157** | 0.93 | 0.55 | 0.26 | 0.73 | *HDAC9* | 4.866277e-38 | . | Hypomethylated |
| **cg13480937** | 0.93 | 0.53 | 0.24 | 0.71 | *ASTN1* | 6.257688e-41 | . | Hypomethylated |
| **cg07548313** | 0.93 | 0.74 | 0.38 | 0.86 | *CD1C* | 2.965958e-90 | . | Hypomethylated |
| **cg17144184** | 0.93 | 0.64 | 0.31 | 0.79 | *.* | 3.867868e-69 | . | Hypomethylated |
| **cg12798564** | 0.93 | 0.75 | 0.38 | 0.87 | *HNRNPA3P6;MIR4444-2* | 3.67323e-80 | . | Hypomethylated |
| **cg26102295** | 0.93 | 0.64 | 0.37 | 0.86 | *.* | 1.746711e-67 | N_Shore | Hypomethylated |
| **cg12849224** | 0.93 | 0.74 | 0.33 | 0.82 | *MYT1L* | 2.17765e-70 | N_Shore | Hypomethylated |
| **cg24537237** | 0.93 | 0.62 | 0.30 | 0.82 | *MUC5B* | 7.398129e-74 | Island | Hypomethylated |
| **cg07834841** | 0.93 | 0.72 | 0.33 | 0.85 | *.* | 3.043955e-59 | . | Hypomethylated |
| **cg14602220** | 0.93 | 0.82 | 0.34 | 0.92 | *RBFOX3* | 5.103407e-104 | . | Hypomethylated |
| **cg22367191** | 0.93 | 0.33 | 0.60 | 0.20 | *RP1-290I10.3* | 9.824422e-69 | N_Shore | Hypermethylated |
| **cg12134633** | 0.93 | 0.61 | 0.82 | 0.37 | *SCG5* | 6.673591e-29 | NA | Hypermethylated |
| **cg04223420** | 0.93 | 0.33 | 0.60 | 0.18 | *PITX1* | 2.218743e-58 | Island | Hypermethylated |
| **cg19099050** | 0.93 | 0.32 | 0.57 | 0.15 | *LHFPL4* | 1.287073e-75 | S_Shore | Hypermethylated |
| **cg17569842** | 0.93 | 0.38 | 0.73 | 0.24 | *RGS12* | 2.340489e-25 | N_Shelf | Hypermethylated |
| **cg22195627** | 0.93 | 0.41 | 0.64 | 0.23 | *CTD-2666L21.1* | 9.7614e-48 | Island | Hypermethylated |
| **cg05860723** | 0.93 | 0.33 | 0.59 | 0.16 | *MYF6* | 1.160602e-52 | Island | Hypermethylated |
| **cg25537993** | 0.93 | 0.32 | 0.64 | 0.16 | *ZSCAN1* | 9.632893e-46 | Island | Hypermethylated |
| **cg20980783** | 0.93 | 0.11 | 0.50 | 0.06 | *TCF24* | 3.577661e-87 | Island | Hypermethylated |
| **cg22399133** | 0.93 | 0.23 | 0.67 | 0.09 | *CRYGD* | 2.07787e-103 | Island | Hypermethylated |
| **cg00396667** | 0.93 | 0.43 | 0.66 | 0.22 | *PITX1* | 8.254872e-36 | Island | Hypermethylated |
| **cg11706983** | 0.93 | 0.36 | 0.67 | 0.22 | *HOXA13;RP1-170O19.14* | 2.148457e-69 | S_Shore | Hypermethylated |
| **cg10703826** | 0.93 | 0.33 | 0.62 | 0.13 | *TBX15* | 2.990526e-80 | Island | Hypermethylated |
| **cg06769546** | 0.93 | 0.23 | 0.52 | 0.11 | *NKX6-2* | 3.307022e-65 | Island | Hypermethylated |
| **cg18983132** | 0.92 | 0.56 | 0.29 | 0.70 | *MAGI2* | 4.159266e-47 | . | Hypomethylated |
| **cg22631387** | 0.92 | 0.67 | 0.38 | 0.78 | *SLIT3* | 4.907927e-75 | . | Hypomethylated |
| **cg22572258** | 0.92 | 0.76 | 0.45 | 0.85 | *KCND2* | 6.715914e-83 | S_Shore | Hypomethylated |
| **cg15203028** | 0.92 | 0.85 | 0.52 | 0.92 | *MUC6* | 2.744135e-75 | Island | Hypomethylated |
| **cg09966455** | 0.92 | 0.75 | 0.42 | 0.82 | *COL20A1* | 1.319806e-62 | S_Shelf | Hypomethylated |
| **cg18889973** | 0.92 | 0.64 | 0.38 | 0.79 | *PTPRN2* | 1.357446e-61 | S_Shore | Hypomethylated |
| **cg15873474** | 0.92 | 0.63 | 0.34 | 0.74 | *CDH4* | 6.249347e-43 | N_Shelf | Hypomethylated |
| **cg06541760** | 0.92 | 0.66 | 0.37 | 0.78 | *.* | 1.826365e-68 | . | Hypomethylated |
| **cg25264630** | 0.92 | 0.53 | 0.25 | 0.66 | *PTPRN2* | 3.184176e-29 | . | Hypomethylated |
| **cg19090585** | 0.92 | 0.55 | 0.33 | 0.74 | *RP11-432M8.18* | 3.275433e-44 | N_Shore | Hypomethylated |
| **cg22135561** | 0.92 | 0.65 | 0.36 | 0.77 | *.* | 2.976477e-63 | . | Hypomethylated |
| **cg06482220** | 0.92 | 0.67 | 0.40 | 0.81 | *.* | 1.482603e-74 | . | Hypomethylated |
| **cg02217063** | 0.92 | 0.66 | 0.38 | 0.79 | *RBFOX1;RP11-420N3.3* | 1.490238e-63 | . | Hypomethylated |
| **cg16009120** | 0.92 | 0.64 | 0.37 | 0.78 | *CLEC4E* | 2.065813e-67 | . | Hypomethylated |
| **cg23378396** | 0.92 | 0.67 | 0.42 | 0.83 | *LINC01019* | 2.78552e-75 | Island | Hypomethylated |
| **cg06692927** | 0.92 | 0.77 | 0.42 | 0.84 | *KRTAP19-1;KRTAP19-3* | 6.496587e-87 | . | Hypomethylated |
| **cg14050129** | 0.92 | 0.64 | 0.40 | 0.81 | *.* | 3.497598e-64 | . | Hypomethylated |
| **cg09363068** | 0.92 | 0.65 | 0.39 | 0.81 | *COX19* | 1.244805e-62 | N_Shelf | Hypomethylated |
| **cg08579420** | 0.92 | 0.74 | 0.43 | 0.84 | *ASIC2* | 4.881499e-76 | . | Hypomethylated |
| **cg21049397** | 0.92 | 0.55 | 0.28 | 0.70 | *LMNTD1* | 3.442345e-35 | . | Hypomethylated |
| **cg09050058** | 0.92 | 0.53 | 0.23 | 0.65 | *.* | 1.114745e-32 | . | Hypomethylated |
| **cg17163729** | 0.92 | 0.43 | 0.21 | 0.63 | *.* | 1.311735e-27 | N_Shelf | Hypomethylated |
| **cg19583265** | 0.92 | 0.67 | 0.37 | 0.79 | *NMS* | 9.934801e-74 | . | Hypomethylated |
| **cg05784088** | 0.92 | 0.55 | 0.32 | 0.74 | *FCRL2* | 4.601294e-59 | . | Hypomethylated |
| **cg21320123** | 0.92 | 0.65 | 0.39 | 0.81 | *DPP6* | 1.717505e-65 | . | Hypomethylated |
| **cg17025555** | 0.92 | 0.63 | 0.33 | 0.75 | *OR6K6* | 5.439199e-49 | . | Hypomethylated |
| **cg20542822** | 0.92 | 0.63 | 0.35 | 0.77 | *COL6A5* | 7.648386e-68 | . | Hypomethylated |
| **cg12255501** | 0.92 | 0.67 | 0.42 | 0.85 | *.* | 4.71635e-53 | S_Shelf | Hypomethylated |
| **cg05618401** | 0.92 | 0.64 | 0.38 | 0.80 | *FAM180A* | 1.858313e-65 | . | Hypomethylated |
| **cg04737845** | 0.92 | 0.68 | 0.42 | 0.84 | *CNTN4;CNTN4-AS1* | 7.458912e-85 | . | Hypomethylated |
| **cg05784226** | 0.92 | 0.66 | 0.37 | 0.80 | *AC027612.4* | 9.405678e-53 | . | Hypomethylated |
| **cg24864399** | 0.92 | 0.65 | 0.37 | 0.80 | *CTD-2533K21.3* | 3.952805e-62 | Island | Hypomethylated |
| **cg25082710** | 0.92 | 0.44 | 0.25 | 0.68 | *IVL* | 4.036998e-47 | . | Hypomethylated |
| **cg06494497** | 0.92 | 0.64 | 0.39 | 0.82 | *KIR2DS4;KIR3DL1* | 3.545846e-75 | . | Hypomethylated |
| **cg26651148** | 0.92 | 0.73 | 0.39 | 0.82 | *PTPRN2* | 4.763743e-76 | . | Hypomethylated |
| **cg11300568** | 0.92 | 0.65 | 0.37 | 0.81 | *DPP6* | 3.898363e-66 | S_Shelf | Hypomethylated |
| **cg14557064** | 0.92 | 0.52 | 0.26 | 0.70 | *.* | 2.621072e-40 | . | Hypomethylated |
| **cg25053164** | 0.92 | 0.65 | 0.38 | 0.82 | *RP11-452N4.1* | 1.161092e-75 | . | Hypomethylated |
| **cg10828127** | 0.92 | 0.76 | 0.41 | 0.84 | *DSCAM* | 2.828706e-74 | N_Shelf | Hypomethylated |
| **cg08918985** | 0.92 | 0.56 | 0.36 | 0.79 | *TCHHL1* | 4.432331e-54 | . | Hypomethylated |
| **cg08009265** | 0.92 | 0.64 | 0.36 | 0.80 | *SPRR2B* | 4.702006e-72 | . | Hypomethylated |
| **cg07623058** | 0.92 | 0.68 | 0.41 | 0.85 | *.* | 6.309414e-74 | . | Hypomethylated |
| **cg02245566** | 0.92 | 0.74 | 0.39 | 0.83 | *OR6K2* | 4.254871e-75 | . | Hypomethylated |
| **cg01819142** | 0.92 | 0.66 | 0.39 | 0.83 | *RP11-238K6.1* | 2.191801e-79 | . | Hypomethylated |
| **cg09916212** | 0.92 | 0.87 | 0.47 | 0.91 | *KCNQ1* | 6.104116e-88 | . | Hypomethylated |
| **cg10767312** | 0.92 | 0.85 | 0.51 | 0.95 | *.* | 1.018444e-82 | . | Hypomethylated |
| **cg15059851** | 0.92 | 0.54 | 0.28 | 0.72 | *IVL* | 7.361172e-50 | . | Hypomethylated |
| **cg13910460** | 0.92 | 0.85 | 0.46 | 0.91 | *CD200* | 7.78323e-86 | . | Hypomethylated |
| **cg16604126** | 0.92 | 0.66 | 0.38 | 0.83 | *OR51E1* | 1.886573e-78 | . | Hypomethylated |
| **cg02130329** | 0.92 | 0.65 | 0.40 | 0.85 | *ANGPT1* | 4.222181e-81 | . | Hypomethylated |
| **cg00067742** | 0.92 | 0.64 | 0.39 | 0.85 | *MYT1L* | 1.42768e-75 | . | Hypomethylated |
| **cg02079463** | 0.92 | 0.83 | 0.43 | 0.89 | *.* | 6.022531e-78 | . | Hypomethylated |
| **cg27043880** | 0.92 | 0.65 | 0.36 | 0.82 | *.* | 1.783975e-77 | . | Hypomethylated |
| **cg23634554** | 0.92 | 0.55 | 0.32 | 0.78 | *.* | 1.429389e-36 | . | Hypomethylated |
| **cg01063579** | 0.92 | 0.73 | 0.39 | 0.86 | *.* | 7.912098e-74 | Island | Hypomethylated |
| **cg07805542** | 0.92 | 0.52 | 0.25 | 0.72 | *PIK3CD* | 2.760954e-45 | S_Shelf | Hypomethylated |
| **cg26840068** | 0.92 | 0.53 | 0.28 | 0.75 | *OR2M7* | 2.625935e-48 | . | Hypomethylated |
| **cg24216893** | 0.92 | 0.63 | 0.34 | 0.81 | *TPO;TPO;TPO;TPO;TPO;TPO;TPO;TPO;TPO* | 2.161454e-48 | . | Hypomethylated |
| **cg26990023** | 0.92 | 0.74 | 0.42 | 0.90 | *SMOC2;SMOC2* | 3.540036e-85 | N_Shore | Hypomethylated |
| **cg07909128** | 0.92 | 0.64 | 0.35 | 0.84 | *SPRR1A* | 9.129885e-81 | . | Hypomethylated |
| **cg07001508** | 0.92 | 0.74 | 0.38 | 0.88 | *PRPF38AP1* | 3.179415e-84 | . | Hypomethylated |
| **cg13661012** | 0.92 | 0.83 | 0.44 | 0.94 | *TPO* | 6.051923e-87 | Island | Hypomethylated |
| **cg19021412** | 0.92 | 0.63 | 0.33 | 0.85 | *.* | 7.496305e-77 | . | Hypomethylated |
| **cg23498518** | 0.92 | 0.74 | 0.38 | 0.91 | *POM121L12* | 3.188301e-94 | S_Shore | Hypomethylated |
| **cg20196215** | 0.92 | 0.64 | 0.34 | 0.88 | *RFESDP1* | 6.809359e-78 | Island | Hypomethylated |
| **cg08892613** | 0.92 | 0.63 | 0.30 | 0.84 | *.* | 3.341633e-55 | . | Hypomethylated |
| **cg22524061** | 0.92 | 0.44 | 0.76 | 0.21 | *OSR2* | 8.096015e-54 | N_Shore | Hypermethylated |
| **cg21009747** | 0.92 | 0.78 | 0.93 | 0.53 | *MYH9* | 6.048235e-15 | NA | Hypermethylated |
| **cg21472506** | 0.92 | 0.33 | 0.65 | 0.14 | *OTX1* | 4.195843e-63 | Island | Hypermethylated |
| **cg21790626** | 0.92 | 0.11 | 0.49 | 0.03 | *AC003006.7; ZNF551* | 2.036102e-93 | Island | Hypermethylated |
| **cg14231297** | 0.92 | 0.44 | 0.61 | 0.21 | *ZSCAN18* | 1.368888e-37 | Island | Hypermethylated |
| **cg19464917** | 0.92 | 0.23 | 0.55 | 0.10 | *ISL2* | 5.647844e-80 | Island | Hypermethylated |
| **cg00970396** | 0.92 | 0.33 | 0.59 | 0.13 | *.* | 4.652919e-70 | NA | Hypermethylated |
| **cg13791254** | 0.92 | 0.42 | 0.64 | 0.23 | *FOXE1* | 2.812494e-45 | Island | Hypermethylated |
| **cg27362525** | 0.92 | 0.34 | 0.58 | 0.18 | *USP6;ZNF232* | 3.198053e-48 | Island | Hypermethylated |
| **cg01959730** | 0.92 | 0.22 | 0.54 | 0.11 | *.* | 3.744051e-79 | N_Shore | Hypermethylated |
| **cg19429281** | 0.92 | 0.31 | 0.59 | 0.14 | *ZNF702P* | 9.583524e-56 | Island | Hypermethylated |
| **cg10171448** | 0.92 | 0.23 | 0.53 | 0.09 | *NKX6-2* | 1.929642e-85 | Island | Hypermethylated |
| **cg00817367** | 0.92 | 0.32 | 0.65 | 0.17 | *GRASP* | 7.865349e-26 | Island | Hypermethylated |
| **cg14353137** | 0.92 | 0.21 | 0.48 | 0.05 | *TCF24* | 4.720392e-90 | Island | Hypermethylated |
| **cg15822765** | 0.92 | 0.43 | 0.65 | 0.23 | *.* | 7.255032e-34 | Island | Hypermethylated |
| **cg25792518** | 0.92 | 0.63 | 0.85 | 0.39 | *ACSF2;CHAD* | 1.082271e-17 | Island | Hypermethylated |
| **cg10659805** | 0.92 | 0.21 | 0.55 | 0.13 | *DLX6-AS1* | 1.740671e-80 | Island | Hypermethylated |
| **cg19172665** | 0.91 | 0.78 | 0.48 | 0.88 | *FREM3* | 1.366002e-75 | N_Shelf | Hypomethylated |
| **cg12493906** | 0.91 | 0.64 | 0.38 | 0.78 | *MMP26* | 5.465855e-59 | . | Hypomethylated |
| **cg17546215** | 0.91 | 0.67 | 0.42 | 0.82 | *.* | 2.451931e-60 | . | Hypomethylated |
| **cg00613945** | 0.91 | 0.63 | 0.37 | 0.78 | *AL354933.1;KRT19P1* | 2.620225e-59 | N_Shore | Hypomethylated |
| **cg22037408** | 0.91 | 0.68 | 0.43 | 0.83 | *ZNF677* | 6.02364e-63 | . | Hypomethylated |
| **cg03458170** | 0.91 | 0.65 | 0.41 | 0.81 | *ZNF311* | 9.359164e-77 | . | Hypomethylated |
| **cg11006791** | 0.91 | 0.67 | 0.39 | 0.79 | *.* | 3.854087e-61 | . | Hypomethylated |
| **cg19918604** | 0.91 | 0.76 | 0.49 | 0.89 | *RP13-150K15.1* | 2.043408e-77 | S_Shore | Hypomethylated |
| **cg02425416** | 0.91 | 0.44 | 0.24 | 0.65 | *IGF2* | 2.239799e-40 | S_Shore | Hypomethylated |
| **cg02983090** | 0.91 | 0.33 | 0.14 | 0.55 | *IL21R* | 7.284808e-24 | . | Hypomethylated |
| **cg07091500** | 0.91 | 0.67 | 0.41 | 0.82 | *RP11-443P15.2* | 1.737779e-68 | . | Hypomethylated |
| **cg25188238** | 0.91 | 0.54 | 0.29 | 0.69 | *XKR4* | 1.956999e-43 | . | Hypomethylated |
| **cg27158573** | 0.91 | 0.67 | 0.39 | 0.79 | *RP11-81M19.1* | 3.88151e-68 | S_Shelf | Hypomethylated |
| **cg15128312** | 0.91 | 0.65 | 0.41 | 0.81 | *ZSCAN18* | 5.088314e-74 | N_Shore | Hypomethylated |
| **cg21506790** | 0.91 | 0.65 | 0.42 | 0.82 | *HBG2* | 2.258238e-58 | . | Hypomethylated |
| **cg23263911** | 0.91 | 0.86 | 0.53 | 0.94 | *.* | 6.880358e-79 | . | Hypomethylated |
| **cg06032977** | 0.91 | 0.87 | 0.51 | 0.92 | *.* | 1.026839e-71 | Island | Hypomethylated |
| **cg22918741** | 0.91 | 0.76 | 0.43 | 0.84 | *.* | 4.56154e-76 | . | Hypomethylated |
| **cg21008530** | 0.91 | 0.74 | 0.44 | 0.85 | *.* | 3.782838e-73 | . | Hypomethylated |
| **cg10508193** | 0.91 | 0.67 | 0.39 | 0.80 | *.* | 5.506893e-59 | . | Hypomethylated |
| **cg00535683** | 0.91 | 0.66 | 0.37 | 0.78 | *SPRR2C* | 9.162929e-65 | . | Hypomethylated |
| **cg13443938** | 0.91 | 0.63 | 0.36 | 0.76 | *.* | 7.767191e-30 | . | Hypomethylated |
| **cg25034991** | 0.91 | 0.83 | 0.50 | 0.91 | *.* | 4.838427e-69 | . | Hypomethylated |
| **cg14216734** | 0.91 | 0.55 | 0.35 | 0.76 | *MNDA;MNDA* | 5.619787e-61 | . | Hypomethylated |
| **cg26986989** | 0.91 | 0.42 | 0.20 | 0.61 | *.* | 1.621756e-29 | . | Hypomethylated |
| **cg04237529** | 0.91 | 0.77 | 0.48 | 0.89 | *OR6K2* | 1.608234e-74 | . | Hypomethylated |
| **cg09967670** | 0.91 | 0.76 | 0.42 | 0.83 | *RP11-654A16.3* | 6.257255e-61 | N_Shelf | Hypomethylated |
| **cg06802346** | 0.91 | 0.76 | 0.44 | 0.85 | *.* | 3.9689e-77 | S_Shore | Hypomethylated |
| **cg00080252** | 0.91 | 0.45 | 0.28 | 0.70 | *.* | 2.494804e-37 | N_Shore | Hypomethylated |
| **cg09066883** | 0.91 | 0.73 | 0.42 | 0.83 | *PTPRN2* | 1.666848e-67 | . | Hypomethylated |
| **cg01129246** | 0.91 | 0.77 | 0.48 | 0.89 | *GRXCR1* | 6.136446e-79 | . | Hypomethylated |
| **cg20546259** | 0.91 | 0.68 | 0.39 | 0.81 | *FSHR* | 5.80344e-72 | . | Hypomethylated |
| **cg27243490** | 0.91 | 0.68 | 0.42 | 0.83 | *SNTG2* | 5.730062e-61 | . | Hypomethylated |
| **cg02185007** | 0.91 | 0.43 | 0.25 | 0.66 | *.* | 1.258904e-36 | . | Hypomethylated |
| **cg22677048** | 0.91 | 0.77 | 0.48 | 0.89 | *AC104389.28;HBE1;HBG2; OR51B2* | 3.809423e-72 | . | Hypomethylated |
| **cg03900492** | 0.91 | 0.54 | 0.30 | 0.72 | *PTPRN2* | 2.738615e-40 | N_Shore | Hypomethylated |
| **cg24316982** | 0.91 | 0.77 | 0.47 | 0.88 | *SPRR4* | 1.423528e-78 | . | Hypomethylated |
| **cg07201003** | 0.91 | 0.66 | 0.42 | 0.84 | *.* | 1.753115e-70 | S_Shelf | Hypomethylated |
| **cg16358696** | 0.91 | 0.63 | 0.32 | 0.73 | *RP11-164C12.1;RP11-266O8.1* | 7.049929e-47 | N_Shore | Hypomethylated |
| **cg07568233** | 0.91 | 0.56 | 0.35 | 0.76 | *RBFOX1* | 1.012143e-63 | . | Hypomethylated |
| **cg27450167** | 0.91 | 0.78 | 0.43 | 0.85 | *TMEM163* | 2.953544e-80 | . | Hypomethylated |
| **cg15392618** | 0.91 | 0.74 | 0.46 | 0.87 | *FMN2* | 1.921874e-67 | N_Shelf | Hypomethylated |
| **cg23462788** | 0.91 | 0.76 | 0.45 | 0.87 | *SCN11A* | 6.058205e-72 | . | Hypomethylated |
| **cg23689630** | 0.91 | 0.54 | 0.30 | 0.72 | *RP11-323P17.2;WDR11-AS1* | 8.549045e-40 | . | Hypomethylated |
| **cg13474719** | 0.91 | 0.65 | 0.39 | 0.81 | *ASTN1* | 1.597565e-65 | . | Hypomethylated |
| **cg13485248** | 0.91 | 0.75 | 0.42 | 0.84 | *.* | 1.989475e-72 | . | Hypomethylated |
| **cg25516542** | 0.91 | 0.54 | 0.34 | 0.76 | *.* | 2.580232e-65 | . | Hypomethylated |
| **cg11595635** | 0.91 | 0.76 | 0.46 | 0.89 | *.* | 1.271587e-80 | . | Hypomethylated |
| **cg03653518** | 0.91 | 0.73 | 0.43 | 0.85 | *CFAP46* | 1.04044e-71 | N_Shore | Hypomethylated |
| **cg15328703** | 0.91 | 0.66 | 0.37 | 0.79 | *TMEM132B* | 1.549957e-65 | . | Hypomethylated |
| **cg01292613** | 0.91 | 0.65 | 0.38 | 0.80 | *NLRP8* | 5.710614e-69 | . | Hypomethylated |
| **cg00584686** | 0.91 | 0.77 | 0.44 | 0.87 | *SNTG2* | 3.30177e-78 | N_Shore | Hypomethylated |
| **cg03522247** | 0.91 | 0.86 | 0.55 | 0.97 | *.* | 9.94759e-78 | . | Hypomethylated |
| **cg27200869** | 0.91 | 0.82 | 0.49 | 0.92 | *PTPRN2* | 9.596115e-83 | . | Hypomethylated |
| **cg01188466** | 0.91 | 0.77 | 0.43 | 0.86 | *RP11-587H10.2* | 8.911176e-74 | . | Hypomethylated |
| **cg20671415** | 0.91 | 0.64 | 0.34 | 0.76 | *SPRR3* | 7.599229e-48 | . | Hypomethylated |
| **cg14582691** | 0.91 | 0.64 | 0.38 | 0.81 | *PTPRN2* | 4.74855e-54 | Island | Hypomethylated |
| **cg27508281** | 0.91 | 0.76 | 0.45 | 0.88 | *.* | 1.603502e-66 | . | Hypomethylated |
| **cg23110407** | 0.91 | 0.66 | 0.41 | 0.83 | *PPP1R17* | 6.791093e-60 | . | Hypomethylated |
| **cg21757970** | 0.91 | 0.82 | 0.46 | 0.88 | *CTD-2046I8.1;RP11-445O3.2* | 6.283571e-72 | S_Shore | Hypomethylated |
| **cg04592560** | 0.91 | 0.66 | 0.39 | 0.82 | *.* | 1.34944e-65 | . | Hypomethylated |
| **cg08721876** | 0.91 | 0.66 | 0.41 | 0.84 | *.* | 1.105872e-83 | . | Hypomethylated |
| **cg09148417** | 0.91 | 0.75 | 0.40 | 0.83 | *LINC01019* | 3.770113e-78 | . | Hypomethylated |
| **cg00946316** | 0.91 | 0.64 | 0.39 | 0.82 | *PTPRN2* | 7.195958e-72 | S_Shore | Hypomethylated |
| **cg20141817** | 0.91 | 0.55 | 0.34 | 0.76 | *SPRR2G* | 1.468413e-59 | . | Hypomethylated |
| **cg05585513** | 0.91 | 0.95 | 0.54 | 0.97 | *LINC01019;RP11-121L11.3* | 1.865066e-73 | . | Hypomethylated |
| **cg24178621** | 0.91 | 0.64 | 0.39 | 0.82 | *SLC6A3* | 2.543672e-70 | . | Hypomethylated |
| **cg12547930** | 0.91 | 0.73 | 0.41 | 0.84 | *EPPIN-WFDC6;HSPD1P21;WFDC6* | 3.715689e-75 | . | Hypomethylated |
| **cg22888848** | 0.91 | 0.67 | 0.37 | 0.80 | *EPHA4* | 8.432028e-66 | . | Hypomethylated |
| **cg14871313** | 0.91 | 0.75 | 0.44 | 0.87 | *RP11-393K12.2* | 1.165705e-74 | . | Hypomethylated |
| **cg25349643** | 0.91 | 0.54 | 0.33 | 0.77 | *MIR181A1HG;MIR181B1* | 1.62354e-45 | . | Hypomethylated |
| **cg00614503** | 0.91 | 0.75 | 0.43 | 0.87 | *CELF2;CELF2-AS2* | 1.175713e-75 | . | Hypomethylated |
| **cg27105990** | 0.91 | 0.44 | 0.26 | 0.70 | *SLC30A8* | 1.66601e-42 | . | Hypomethylated |
| **cg25597535** | 0.91 | 0.46 | 0.30 | 0.73 | *NEUROD6;NEUROD6* | 7.874748e-30 | S_Shelf | Hypomethylated |
| **cg08160063** | 0.91 | 0.64 | 0.41 | 0.84 | *RBFOX1* | 2.6589e-71 | S_Shore | Hypomethylated |
| **cg04436383** | 0.91 | 0.77 | 0.44 | 0.87 | *SLC10A6;SLC10A6* | 3.862647e-78 | . | Hypomethylated |
| **cg12412531** | 0.91 | 0.83 | 0.50 | 0.93 | *CACNA2D4;LRTM2* | 1.635256e-80 | . | Hypomethylated |
| **cg01782790** | 0.91 | 0.93 | 0.51 | 0.95 | *RP11-408E1.1* | 4.064607e-65 | Island | Hypomethylated |
| **cg23520574** | 0.91 | 0.64 | 0.34 | 0.78 | *MMP26;OR51A7* | 5.37743e-40 | . | Hypomethylated |
| **cg25863289** | 0.91 | 0.87 | 0.52 | 0.96 | *TM2D3* | 2.636124e-75 | S_Shore | Hypomethylated |
| **cg08414888** | 0.91 | 0.75 | 0.44 | 0.88 | *PTPRN2* | 6.560899e-83 | . | Hypomethylated |
| **cg15027050** | 0.91 | 0.75 | 0.44 | 0.88 | *.* | 2.659904e-83 | . | Hypomethylated |
| **cg04152326** | 0.91 | 0.73 | 0.44 | 0.88 | *TPO* | 2.708258e-71 | . | Hypomethylated |
| **cg23822732** | 0.91 | 0.63 | 0.34 | 0.78 | *.* | 1.28344e-57 | . | Hypomethylated |
| **cg10976626** | 0.91 | 0.74 | 0.40 | 0.84 | *CD1E* | 6.19819e-72 | . | Hypomethylated |
| **cg26718232** | 0.91 | 0.88 | 0.51 | 0.95 | *.* | 1.240677e-81 | . | Hypomethylated |
| **cg12297440** | 0.91 | 0.83 | 0.49 | 0.93 | *PTPRN2* | 5.324045e-79 | N_Shore | Hypomethylated |
| **cg00212119** | 0.91 | 0.75 | 0.43 | 0.87 | *RP11-281O15.4* | 6.627988e-79 | . | Hypomethylated |
| **cg00186207** | 0.91 | 0.66 | 0.40 | 0.85 | *.* | 5.244691e-80 | . | Hypomethylated |
| **cg24881910** | 0.91 | 0.54 | 0.29 | 0.74 | *FCRL3* | 4.455421e-52 | . | Hypomethylated |
| **cg10961733** | 0.91 | 0.67 | 0.41 | 0.86 | *.* | 1.65926e-62 | . | Hypomethylated |
| **cg18735609** | 0.91 | 0.75 | 0.43 | 0.88 | *.* | 4.595534e-81 | . | Hypomethylated |
| **cg01867950** | 0.91 | 0.74 | 0.38 | 0.83 | *TMEM71* | 2.415085e-69 | . | Hypomethylated |
| **cg08393822** | 0.91 | 0.65 | 0.30 | 0.75 | *UNC80* | 8.040773e-43 | . | Hypomethylated |
| **cg08323777** | 0.91 | 0.64 | 0.38 | 0.83 | *.* | 1.311585e-74 | . | Hypomethylated |
| **cg19602728** | 0.91 | 0.53 | 0.22 | 0.68 | *.* | 3.076981e-30 | S_Shore | Hypomethylated |
| **cg10767420** | 0.91 | 0.64 | 0.33 | 0.79 | *.* | 3.273446e-56 | . | Hypomethylated |
| **cg02456218** | 0.91 | 0.64 | 0.35 | 0.80 | *BTNL9* | 5.69406e-75 | N_Shelf | Hypomethylated |
| **cg01445411** | 0.91 | 0.76 | 0.41 | 0.87 | *SNTG2* | 4.200423e-82 | . | Hypomethylated |
| **cg15486123** | 0.91 | 0.64 | 0.36 | 0.81 | *IVL* | 2.968274e-61 | . | Hypomethylated |
| **cg24646841** | 0.91 | 0.73 | 0.42 | 0.88 | *AC011899.10;PTPRN2* | 1.133916e-74 | . | Hypomethylated |
| **cg21441360** | 0.91 | 0.74 | 0.40 | 0.86 | *.* | 2.396773e-69 | . | Hypomethylated |
| **cg02583228** | 0.91 | 0.65 | 0.32 | 0.78 | *GABRB3* | 9.729873e-57 | . | Hypomethylated |
| **cg27170427** | 0.91 | 0.63 | 0.35 | 0.82 | *RIMBP2* | 1.558705e-51 | . | Hypomethylated |
| **cg06244947** | 0.91 | 0.73 | 0.36 | 0.84 | *.* | 3.251722e-81 | S_Shore | Hypomethylated |
| **cg22946562** | 0.91 | 0.65 | 0.37 | 0.85 | *MYT1L* | 3.133884e-63 | . | Hypomethylated |
| **cg26479667** | 0.91 | 0.52 | 0.24 | 0.72 | *.* | 9.545033e-26 | . | Hypomethylated |
| **cg13608273** | 0.91 | 0.64 | 0.36 | 0.84 | *.* | 4.364333e-67 | . | Hypomethylated |
| **cg26040583** | 0.91 | 0.94 | 0.48 | 0.96 | *AC004006.2* | 3.650213e-72 | . | Hypomethylated |
| **cg27099991** | 0.91 | 0.84 | 0.45 | 0.93 | *NLRP2* | 1.084936e-87 | S_Shore | Hypomethylated |
| **cg06966242** | 0.91 | 0.72 | 0.42 | 0.90 | *AJAP1* | 8.138583e-67 | Island | Hypomethylated |
| **cg02216247** | 0.91 | 0.75 | 0.42 | 0.90 | *NTSR1* | 4.105146e-92 | S_Shore | Hypomethylated |
| **cg24254317** | 0.91 | 0.54 | 0.30 | 0.79 | *PTPRN2* | 2.345923e-62 | . | Hypomethylated |
| **cg10547893** | 0.91 | 0.77 | 0.43 | 0.92 | *RP11-114H7.2* | 2.603881e-79 | Island | Hypomethylated |
| **cg09149672** | 0.91 | 0.64 | 0.35 | 0.84 | *CD1C* | 2.465045e-79 | . | Hypomethylated |
| **cg19335742** | 0.91 | 0.74 | 0.36 | 0.86 | *PAK6* | 2.742636e-53 | . | Hypomethylated |
| **cg12584111** | 0.91 | 0.74 | 0.39 | 0.89 | *MOXD2P* | 1.482144e-75 | . | Hypomethylated |
| **cg24311564** | 0.91 | 0.62 | 0.39 | 0.90 | *DPP6* | 2.332794e-70 | Island | Hypomethylated |
| **cg01775802** | 0.91 | 0.52 | 0.26 | 0.77 | *RGS6* | 2.305876e-31 | . | Hypomethylated |
| **cg03910363** | 0.91 | 0.82 | 0.39 | 0.91 | *IGSF9B* | 7.893172e-86 | N_Shore | Hypomethylated |
| **cg13403271** | 0.91 | 0.76 | 0.37 | 0.89 | *PTPRN2* | 4.869667e-94 | . | Hypomethylated |
| **cg16035036** | 0.91 | 0.82 | 0.43 | 0.96 | *LINC01511* | 2.605825e-87 | . | Hypomethylated |
| **cg06500727** | 0.91 | 0.73 | 0.33 | 0.87 | *TPO* | 8.660143e-69 | . | Hypomethylated |
| **cg23864180** | 0.91 | 0.83 | 0.32 | 0.89 | *ADARB2* | 5.025464e-74 | S_Shelf | Hypomethylated |
| **cg23391785** | 0.91 | 0.31 | 0.54 | 0.12 | *DNM3* | 3.140217e-69 | Island | Hypermethylated |
| **cg22167515** | 0.91 | 0.22 | 0.49 | 0.07 | *.* | 5.967618e-89 | Island | Hypermethylated |
| **cg25032595** | 0.91 | 0.32 | 0.62 | 0.15 | *CLDN10* | 3.1665e-62 | Island | Hypermethylated |
| **cg02970836** | 0.91 | 0.23 | 0.60 | 0.12 | *AJ003147.9* | 1.288828e-45 | N_Shore | Hypermethylated |
| **cg00033551** | 0.91 | 0.43 | 0.79 | 0.25 | *MGRN1* | 1.270709e-58 | NA | Hypermethylated |
| **cg06829686** | 0.91 | 0.32 | 0.59 | 0.14 | *.* | 6.476247e-57 | Island | Hypermethylated |
| **cg03192598** | 0.91 | 0.41 | 0.60 | 0.12 | *CTD-2666L21.1* | 1.009772e-55 | Island | Hypermethylated |
| **cg14644001** | 0.91 | 0.23 | 0.59 | 0.11 | *PRRT1* | 7.839041e-79 | Island | Hypermethylated |
| **cg01368068** | 0.90 | 0.66 | 0.40 | 0.80 | *MAGI2* | 1.442302e-56 | . | Hypomethylated |
| **cg09732255** | 0.90 | 0.54 | 0.30 | 0.70 | *MYT1L* | 1.111989e-49 | S_Shore | Hypomethylated |
| **cg10999347** | 0.90 | 0.64 | 0.35 | 0.75 | *.* | 1.303308e-61 | . | Hypomethylated |
| **cg00263146** | 0.90 | 0.54 | 0.31 | 0.71 | *BASP1* | 8.838217e-58 | S_Shelf | Hypomethylated |
| **cg02484411** | 0.90 | 0.74 | 0.41 | 0.82 | *AC093326.1* | 4.350938e-79 | N_Shore | Hypomethylated |
| **cg27096572** | 0.90 | 0.58 | 0.40 | 0.80 | *SMIM21* | 3.402862e-55 | . | Hypomethylated |
| **cg07448997** | 0.90 | 0.76 | 0.46 | 0.86 | *MAGI2* | 1.567367e-66 | . | Hypomethylated |
| **cg05353571** | 0.90 | 0.65 | 0.42 | 0.82 | *.* | 1.007577e-70 | S_Shore | Hypomethylated |
| **cg24166463** | 0.90 | 0.55 | 0.36 | 0.76 | *MMP26;OR51T1* | 4.904105e-54 | . | Hypomethylated |
| **cg05503824** | 0.90 | 0.67 | 0.45 | 0.85 | *ATP13A5* | 7.764273e-75 | . | Hypomethylated |
| **cg12565335** | 0.90 | 0.74 | 0.45 | 0.86 | *MYT1L* | 4.004291e-73 | . | Hypomethylated |
| **cg19746375** | 0.90 | 0.64 | 0.41 | 0.81 | *PTPRN2* | 2.135174e-58 | . | Hypomethylated |
| **cg03292377** | 0.90 | 0.44 | 0.25 | 0.65 | *MIR7515HG* | 1.846651e-29 | . | Hypomethylated |
| **cg04438395** | 0.90 | 0.54 | 0.26 | 0.67 | *.* | 2.290113e-26 | . | Hypomethylated |
| **cg24694502** | 0.90 | 0.63 | 0.42 | 0.82 | *AJAP1* | 1.828285e-59 | . | Hypomethylated |
| **cg14128018** | 0.90 | 0.65 | 0.38 | 0.78 | *.* | 6.71634e-63 | . | Hypomethylated |
| **cg06382093** | 0.90 | 0.77 | 0.43 | 0.83 | *.* | 1.171896e-41 | N_Shelf | Hypomethylated |
| **cg17840707** | 0.90 | 0.57 | 0.33 | 0.74 | *AGBL1* | 7.479879e-37 | . | Hypomethylated |
| **cg03327829** | 0.90 | 0.64 | 0.39 | 0.79 | *.* | 9.298641e-62 | S_Shelf | Hypomethylated |
| **cg05731713** | 0.90 | 0.94 | 0.57 | 0.97 | *PTPRN2* | 1.313561e-62 | . | Hypomethylated |
| **cg02129712** | 0.90 | 0.77 | 0.48 | 0.89 | *XKR4* | 3.841226e-75 | . | Hypomethylated |
| **cg18650367** | 0.90 | 0.53 | 0.21 | 0.61 | *.* | 2.011188e-42 | . | Hypomethylated |
| **cg14551991** | 0.90 | 0.55 | 0.35 | 0.75 | *.* | 5.635554e-40 | . | Hypomethylated |
| **cg14935163** | 0.90 | 0.84 | 0.53 | 0.93 | *TPO* | 1.30726e-72 | . | Hypomethylated |
| **cg16468388** | 0.90 | 0.63 | 0.36 | 0.76 | *.* | 1.338885e-69 | S_Shore | Hypomethylated |
| **cg22883887** | 0.90 | 0.77 | 0.47 | 0.87 | *RIMBP2* | 1.00702e-73 | . | Hypomethylated |
| **cg26254916** | 0.90 | 0.77 | 0.48 | 0.88 | *RP11-432M8.13* | 3.027946e-73 | . | Hypomethylated |
| **cg22475082** | 0.90 | 0.76 | 0.45 | 0.85 | *NLRP2* | 3.640017e-75 | S_Shelf | Hypomethylated |
| **cg11066776** | 0.90 | 0.87 | 0.51 | 0.92 | *SPRR4* | 1.633566e-72 | . | Hypomethylated |
| **cg12779445** | 0.90 | 0.75 | 0.45 | 0.85 | *MIR153-2;PTPRN2* | 3.044681e-63 | N_Shore | Hypomethylated |
| **cg04804542** | 0.90 | 0.52 | 0.27 | 0.67 | *RBFOX3* | 1.673426e-52 | N_Shelf | Hypomethylated |
| **cg22479532** | 0.90 | 0.69 | 0.42 | 0.83 | *LPHN3* | 6.535227e-60 | . | Hypomethylated |
| **cg09893424** | 0.90 | 0.63 | 0.35 | 0.76 | *CTD-2533K21.3* | 5.767184e-59 | N_Shore | Hypomethylated |
| **cg10078688** | 0.90 | 0.79 | 0.48 | 0.89 | *OXR1;OXR1* | 1.226721e-75 | . | Hypomethylated |
| **cg03345017** | 0.90 | 0.63 | 0.39 | 0.80 | *.* | 3.455711e-68 | . | Hypomethylated |
| **cg23338195** | 0.90 | 0.54 | 0.31 | 0.72 | *SLC30A8* | 3.393185e-39 | . | Hypomethylated |
| **cg15375772** | 0.90 | 0.61 | 0.36 | 0.77 | *TPO* | 7.340815e-49 | . | Hypomethylated |
| **cg21341435** | 0.90 | 0.66 | 0.42 | 0.83 | *PTPRN2* | 7.848089e-65 | . | Hypomethylated |
| **cg12090430** | 0.90 | 0.65 | 0.42 | 0.83 | *ADARB2* | 8.257252e-71 | . | Hypomethylated |
| **cg23694187** | 0.90 | 0.75 | 0.44 | 0.85 | *PTPRN2* | 9.725586e-76 | . | Hypomethylated |
| **cg25628429** | 0.90 | 0.66 | 0.38 | 0.79 | *OR4C12* | 5.259332e-62 | . | Hypomethylated |
| **cg07513789** | 0.90 | 0.68 | 0.41 | 0.82 | *FBN2;SLC27A6* | 1.096672e-67 | . | Hypomethylated |
| **cg13797468** | 0.90 | 0.64 | 0.34 | 0.76 | *.* | 1.440751e-60 | . | Hypomethylated |
| **cg07007544** | 0.90 | 0.66 | 0.41 | 0.82 | *GLRA1* | 1.52539e-54 | N_Shelf | Hypomethylated |
| **cg27572053** | 0.90 | 0.66 | 0.39 | 0.80 | *PTPRN2* | 8.30938e-73 | . | Hypomethylated |
| **cg12378867** | 0.90 | 0.94 | 0.55 | 0.96 | *ADCY2* | 2.845578e-59 | . | Hypomethylated |
| **cg13600716** | 0.90 | 0.76 | 0.44 | 0.85 | *CLEC1A* | 4.134341e-35 | . | Hypomethylated |
| **cg18771659** | 0.90 | 0.75 | 0.46 | 0.87 | *SPRR1B* | 4.882285e-68 | . | Hypomethylated |
| **cg03002586** | 0.90 | 0.65 | 0.35 | 0.77 | *PLD5* | 1.07552e-39 | . | Hypomethylated |
| **cg07048608** | 0.90 | 0.65 | 0.36 | 0.78 | *SPTA1* | 8.716691e-57 | . | Hypomethylated |
| **cg13123585** | 0.90 | 0.68 | 0.36 | 0.77 | *RP11-572C21.1;TMEM132D* | 1.31795e-25 | N_Shore | Hypomethylated |
| **cg12071044** | 0.90 | 0.75 | 0.41 | 0.83 | *PTPRN2* | 1.970058e-74 | . | Hypomethylated |
| **cg08279693** | 0.90 | 0.66 | 0.40 | 0.81 | *LCE2B* | 1.534424e-72 | . | Hypomethylated |
| **cg14036821** | 0.90 | 0.77 | 0.46 | 0.88 | *.* | 4.08645e-75 | . | Hypomethylated |
| **cg19130189** | 0.90 | 0.57 | 0.34 | 0.76 | *MMP16* | 2.343917e-49 | . | Hypomethylated |
| **cg19696718** | 0.90 | 0.65 | 0.39 | 0.81 | *COL6A3* | 4.74907e-62 | . | Hypomethylated |
| **cg02478172** | 0.90 | 0.78 | 0.51 | 0.93 | *PTPRN2* | 4.665094e-84 | . | Hypomethylated |
| **cg19370043** | 0.90 | 0.85 | 0.49 | 0.91 | *PRRX1* | 1.044146e-63 | . | Hypomethylated |
| **cg09553753** | 0.90 | 0.74 | 0.42 | 0.84 | *.* | 1.106067e-77 | S_Shore | Hypomethylated |
| **cg18082788** | 0.90 | 0.42 | 0.19 | 0.61 | *ZC3H12D* | 1.375109e-38 | . | Hypomethylated |
| **cg18580232** | 0.90 | 0.75 | 0.45 | 0.87 | *DPP10* | 8.280208e-74 | . | Hypomethylated |
| **cg11266667** | 0.90 | 0.74 | 0.41 | 0.83 | *CT55* | 3.344685e-68 | . | Hypomethylated |
| **cg22601108** | 0.90 | 0.63 | 0.32 | 0.74 | *OR6K6* | 5.646751e-35 | . | Hypomethylated |
| **cg19278235** | 0.90 | 0.76 | 0.47 | 0.89 | *LINC01249* | 1.259586e-72 | . | Hypomethylated |
| **cg13087761** | 0.90 | 0.73 | 0.43 | 0.85 | *FSCN1* | 2.917606e-52 | S_Shore | Hypomethylated |
| **cg06502659** | 0.90 | 0.67 | 0.40 | 0.83 | *.* | 4.100621e-73 | N_Shelf | Hypomethylated |
| **cg19997181** | 0.90 | 0.77 | 0.43 | 0.86 | *.* | 3.375058e-85 | . | Hypomethylated |
| **cg11476737** | 0.90 | 0.62 | 0.41 | 0.84 | *.* | 2.555475e-61 | Island | Hypomethylated |
| **cg09560465** | 0.90 | 0.65 | 0.40 | 0.82 | *.* | 1.402117e-59 | . | Hypomethylated |
| **cg25806491** | 0.90 | 0.68 | 0.40 | 0.83 | *AC067956.1* | 2.265489e-56 | . | Hypomethylated |
| **cg23639196** | 0.90 | 0.62 | 0.35 | 0.78 | *.* | 1.115167e-53 | S_Shelf | Hypomethylated |
| **cg11901695** | 0.90 | 0.86 | 0.51 | 0.94 | *SNTG2* | 5.664017e-75 | . | Hypomethylated |
| **cg02394701** | 0.90 | 0.64 | 0.38 | 0.80 | *.* | 2.968254e-70 | . | Hypomethylated |
| **cg21208734** | 0.90 | 0.52 | 0.28 | 0.71 | *TENM3* | 1.243777e-41 | . | Hypomethylated |
| **cg21605238** | 0.90 | 0.67 | 0.39 | 0.82 | *FREM2* | 5.810497e-53 | S_Shelf | Hypomethylated |
| **cg07728307** | 0.90 | 0.65 | 0.38 | 0.81 | *ADARB2* | 9.851382e-67 | N_Shore | Hypomethylated |
| **cg17364913** | 0.90 | 0.87 | 0.54 | 0.97 | *PTPRN2* | 4.806108e-81 | . | Hypomethylated |
| **cg03151258** | 0.90 | 0.75 | 0.46 | 0.89 | *CYP4F23P* | 1.413956e-76 | N_Shore | Hypomethylated |
| **cg23170495** | 0.90 | 0.64 | 0.40 | 0.83 | *.* | 5.538955e-66 | . | Hypomethylated |
| **cg17470497** | 0.90 | 0.64 | 0.39 | 0.82 | *OR2M4* | 6.977864e-72 | . | Hypomethylated |
| **cg18627442** | 0.90 | 0.64 | 0.44 | 0.87 | *TMEM132C* | 2.866197e-58 | Island | Hypomethylated |
| **cg00949578** | 0.90 | 0.75 | 0.42 | 0.85 | *SMIM21* | 1.913857e-77 | . | Hypomethylated |
| **cg13510648** | 0.90 | 0.93 | 0.53 | 0.96 | *VCX3A* | 3.366086e-68 | . | Hypomethylated |
| **cg01890568** | 0.90 | 0.42 | 0.27 | 0.70 | *ASIC2;RP11-40A13.1* | 1.156133e-36 | . | Hypomethylated |
| **cg08242576** | 0.90 | 0.66 | 0.39 | 0.82 | *LELP1* | 2.036252e-60 | . | Hypomethylated |
| **cg02638348** | 0.90 | 0.74 | 0.43 | 0.87 | *CFAP46* | 2.736931e-69 | N_Shore | Hypomethylated |
| **cg01973908** | 0.90 | 0.73 | 0.42 | 0.85 | *AC009518.4* | 5.51649e-69 | N_Shore | Hypomethylated |
| **cg05887869** | 0.90 | 0.63 | 0.36 | 0.79 | *CTD-2143L24.1* | 1.973644e-64 | . | Hypomethylated |
| **cg25138418** | 0.90 | 0.83 | 0.50 | 0.93 | *ST8SIA5* | 1.511278e-77 | Island | Hypomethylated |
| **cg22515937** | 0.90 | 0.65 | 0.37 | 0.80 | *FCRL1* | 2.252286e-55 | . | Hypomethylated |
| **cg09302922** | 0.90 | 0.65 | 0.32 | 0.75 | *.* | 1.502916e-57 | S_Shelf | Hypomethylated |
| **cg08689708** | 0.90 | 0.74 | 0.44 | 0.87 | *MYT1L* | 3.206887e-77 | . | Hypomethylated |
| **cg25740250** | 0.90 | 0.63 | 0.37 | 0.81 | *PTPRN2* | 2.338612e-64 | N_Shelf | Hypomethylated |
| **cg13303464** | 0.90 | 0.74 | 0.45 | 0.89 | *.* | 7.642027e-80 | . | Hypomethylated |
| **cg14520280** | 0.90 | 0.66 | 0.39 | 0.83 | *.* | 1.491307e-68 | . | Hypomethylated |
| **cg14566081** | 0.90 | 0.65 | 0.37 | 0.81 | *RP11-259O2.1* | 1.342613e-60 | . | Hypomethylated |
| **cg01578265** | 0.90 | 0.66 | 0.39 | 0.82 | *.* | 3.366281e-60 | . | Hypomethylated |
| **cg16571642** | 0.90 | 0.74 | 0.46 | 0.90 | *PTPRN2* | 1.709365e-81 | . | Hypomethylated |
| **cg24460817** | 0.90 | 0.65 | 0.40 | 0.84 | *OR6F1;RP11-634B7.4* | 6.934258e-74 | . | Hypomethylated |
| **cg14148690** | 0.90 | 0.74 | 0.42 | 0.86 | *AC113607.3* | 5.973953e-58 | . | Hypomethylated |
| **cg05349089** | 0.90 | 0.63 | 0.38 | 0.82 | *.* | 5.05572e-60 | . | Hypomethylated |
| **cg19740859** | 0.90 | 0.82 | 0.48 | 0.92 | *MYT1L* | 8.339449e-72 | Island | Hypomethylated |
| **cg14818277** | 0.90 | 0.65 | 0.38 | 0.83 | *.* | 7.634488e-75 | . | Hypomethylated |
| **cg03420866** | 0.90 | 0.83 | 0.48 | 0.92 | *TMEM132D* | 8.645043e-80 | . | Hypomethylated |
| **cg20159095** | 0.90 | 0.67 | 0.38 | 0.83 | *PTPRN2* | 6.559775e-65 | . | Hypomethylated |
| **cg23207876** | 0.90 | 0.72 | 0.37 | 0.82 | *AC004699.1;CASP14* | 2.646497e-76 | . | Hypomethylated |
| **cg12402831** | 0.90 | 0.76 | 0.39 | 0.84 | *PLD5* | 2.51857e-49 | . | Hypomethylated |
| **cg15115960** | 0.90 | 0.74 | 0.38 | 0.83 | *.* | 1.775232e-73 | Island | Hypomethylated |
| **cg20853771** | 0.90 | 0.62 | 0.35 | 0.80 | *NPTX1* | 4.143922e-44 | N_Shore | Hypomethylated |
| **cg18087023** | 0.90 | 0.65 | 0.36 | 0.82 | *SPRR2D* | 6.223688e-55 | . | Hypomethylated |
| **cg18918116** | 0.90 | 0.66 | 0.44 | 0.89 | *SYT9* | 4.785306e-70 | . | Hypomethylated |
| **cg08246366** | 0.90 | 0.73 | 0.45 | 0.91 | *PTPRN2* | 4.901811e-73 | S_Shelf | Hypomethylated |
| **cg13909949** | 0.90 | 0.73 | 0.40 | 0.86 | *.* | 4.406458e-72 | . | Hypomethylated |
| **cg18781372** | 0.90 | 0.64 | 0.38 | 0.83 | *SCN11A* | 9.303212e-59 | . | Hypomethylated |
| **cg25022271** | 0.90 | 0.84 | 0.49 | 0.95 | *.* | 2.039997e-79 | . | Hypomethylated |
| **cg06937491** | 0.90 | 0.74 | 0.49 | 0.94 | *CACNA2D4;LRTM2* | 1.243167e-74 | . | Hypomethylated |
| **cg10256733** | 0.90 | 0.75 | 0.44 | 0.90 | *MYT1* | 3.085319e-81 | . | Hypomethylated |
| **cg17980184** | 0.90 | 0.74 | 0.42 | 0.88 | *PTPRN2* | 2.178818e-38 | S_Shore | Hypomethylated |
| **cg14215310** | 0.90 | 0.75 | 0.43 | 0.89 | *.* | 5.354825e-76 | . | Hypomethylated |
| **cg14909201** | 0.90 | 0.73 | 0.40 | 0.87 | *LINC00368* | 2.219587e-76 | S_Shelf | Hypomethylated |
| **cg14966325** | 0.90 | 0.64 | 0.32 | 0.78 | *SPRR2A* | 5.829925e-47 | . | Hypomethylated |
| **cg19561972** | 0.90 | 0.64 | 0.36 | 0.83 | *.* | 1.188031e-55 | . | Hypomethylated |
| **cg10416814** | 0.90 | 0.32 | 0.20 | 0.67 | *RFESDP1* | 2.669714e-16 | Island | Hypomethylated |
| **cg08844913** | 0.90 | 0.83 | 0.43 | 0.89 | *.* | 4.604035e-86 | . | Hypomethylated |
| **cg04498153** | 0.90 | 0.73 | 0.36 | 0.83 | *PTPRN2* | 2.696643e-79 | N_Shore | Hypomethylated |
| **cg18730194** | 0.90 | 0.76 | 0.40 | 0.87 | *LINC00701* | 3.110781e-79 | S_Shore | Hypomethylated |
| **cg22506490** | 0.90 | 0.55 | 0.34 | 0.81 | *ASTN1* | 1.294165e-57 | . | Hypomethylated |
| **cg04757389** | 0.90 | 0.73 | 0.45 | 0.92 | *PTPRS* | 5.014748e-78 | . | Hypomethylated |
| **cg13547712** | 0.90 | 0.83 | 0.48 | 0.95 | *.* | 4.05066e-82 | N_Shore | Hypomethylated |
| **cg18820060** | 0.90 | 0.74 | 0.39 | 0.87 | *LINC01019* | 1.316492e-78 | S_Shore | Hypomethylated |
| **cg17277001** | 0.90 | 0.64 | 0.34 | 0.81 | *SLC6A18* | 4.410638e-41 | S_Shore | Hypomethylated |
| **cg04892646** | 0.90 | 0.76 | 0.42 | 0.89 | *.* | 3.338728e-85 | . | Hypomethylated |
| **cg05672569** | 0.90 | 0.74 | 0.41 | 0.88 | *PTPRN2* | 6.576185e-56 | . | Hypomethylated |
| **cg17777531** | 0.90 | 0.74 | 0.39 | 0.88 | *DCAF4L2* | 3.833435e-77 | N_Shore | Hypomethylated |
| **cg08957001** | 0.90 | 0.84 | 0.41 | 0.89 | *TSNARE1* | 7.697957e-76 | S_Shore | Hypomethylated |
| **cg02223801** | 0.90 | 0.73 | 0.36 | 0.84 | *PTPRN2* | 1.075972e-60 | N_Shelf | Hypomethylated |
| **cg18093771** | 0.90 | 0.74 | 0.36 | 0.85 | *ADARB2* | 8.136423e-75 | S_Shelf | Hypomethylated |
| **cg01140416** | 0.90 | 0.74 | 0.38 | 0.88 | *.* | 9.141646e-72 | . | Hypomethylated |
| **cg23643466** | 0.90 | 0.83 | 0.43 | 0.93 | *.* | 2.423047e-85 | . | Hypomethylated |
| **cg15167871** | 0.90 | 0.63 | 0.34 | 0.84 | *TCERG1L* | 1.049732e-52 | N_Shelf | Hypomethylated |
| **cg09363841** | 0.90 | 0.83 | 0.42 | 0.92 | *.* | 7.642906e-76 | . | Hypomethylated |
| **cg20042908** | 0.90 | 0.82 | 0.42 | 0.93 | *.* | 3.029182e-78 | . | Hypomethylated |
| **cg19006220** | 0.90 | 0.83 | 0.40 | 0.93 | *.* | 2.36216e-69 | Island | Hypomethylated |
| **cg15549700** | 0.90 | 0.83 | 0.41 | 0.94 | *AJAP1* | 2.792932e-87 | N_Shelf | Hypomethylated |
| **cg06760904** | 0.90 | 0.83 | 0.36 | 0.90 | *AC093390.1;MYT1L* | 2.807467e-79 | . | Hypomethylated |
| **cg20064139** | 0.90 | 0.83 | 0.38 | 0.93 | *RP1-269M15.3* | 9.445145e-87 | S_Shelf | Hypomethylated |
| **cg27104173** | 0.90 | 0.84 | 0.41 | 0.96 | *PTPRN2* | 4.761082e-89 | . | Hypomethylated |
| **cg13782274** | 0.90 | 0.72 | 0.33 | 0.88 | *KCNQ2* | 4.278515e-73 | Island | Hypomethylated |
| **cg12415687** | 0.90 | 0.72 | 0.31 | 0.87 | *PTPRN2* | 4.566483e-76 | . | Hypomethylated |
| **cg14757228** | 0.90 | 0.35 | 0.65 | 0.22 | *PRRT1* | 3.84008e-53 | Island | Hypermethylated |
| **cg03497652** | 0.90 | 0.63 | 0.91 | 0.51 | *ANKS3* | 8.855726e-13 | NA | Hypermethylated |
| **cg14888916** | 0.90 | 0.26 | 0.54 | 0.14 | *STK3* | 2.1339e-81 | N_Shore | Hypermethylated |
| **cg07779120** | 0.90 | 0.32 | 0.61 | 0.12 | *IGF1R* | 1.891493e-50 | Island | Hypermethylated |
| **cg17400476** | 0.90 | 0.22 | 0.57 | 0.09 | *RP1-170O19.14* | 6.100525e-78 | N_Shore | Hypermethylated |
| **cg04823311** | 0.90 | 0.11 | 0.50 | 0.06 | *AC005013.5;TRIL* | 2.10385e-75 | Island | Hypermethylated |
| **cg18756179** | 0.90 | 0.25 | 0.58 | 0.17 | *CTD-2126E3.3; FLJ26850;ZNF473* | 7.232665e-82 | S_Shore | Hypermethylated |
| **cg01528052** | 0.90 | 0.42 | 0.61 | 0.20 | *.* | 5.899767e-37 | S_Shore | Hypermethylated |
| **cg26149244** | 0.90 | 0.11 | 0.52 | 0.06 | *.* | 1.81825e-88 | Island | Hypermethylated |
| **cg02710296** | 0.90 | 0.55 | 0.81 | 0.41 | *SHCBP1L* | 2.80005e-18 | Island | Hypermethylated |
| **cg22557662** | 0.90 | 0.32 | 0.60 | 0.17 | *PPP1R14A; SPINT2* | 4.676559e-44 | Island | Hypermethylated |
| **cg19852958** | 0.90 | 0.23 | 0.55 | 0.08 | *NKX3-2* | 7.671553e-79 | Island | Hypermethylated |
| **cg17264670** | 0.90 | 0.52 | 0.64 | 0.22 | *RGS17* | 1.808268e-39 | Island | Hypermethylated |
| **cg11660826** | 0.90 | 0.23 | 0.50 | 0.10 | *ULBP1* | 4.222683e-66 | Island | Hypermethylated |
| **cg03544320** | 0.90 | 0.31 | 0.56 | 0.14 | *CRMP1* | 2.32195e-49 | Island | Hypermethylated |
| **cg03679755** | 0.90 | 0.33 | 0.60 | 0.19 | *TPM4P1* | 3.446249e-41 | Island | Hypermethylated |

**Table J. Top 147 RNA transcripts (LCN-RNA-AUC) based on Single gene threshold based approach for cancer versus normal classification.**

| **Ensemble_ID** | **Class** | **Gene symbol** | **AUC** | **Threshold (log2FPKM)** | **Mean in Cancer (log2FPKM)** | **Mean in normal(log2FPKM)** | **mean_difference** | **Standard deviation in cancer** | **Standard deviation in normal** | **Bonferroni adjusted p-value** |
| --- | --- | --- | --- | --- | --- | --- | --- | --- | --- | --- |
| **ENSG00000182566.11** | protein coding | *CLEC4G* | 0.99 | 2.90 | 0.46 | 4.83 | -4.37 | 0.88 | 0.69 | 1.03E-36 |
| **ENSG00000104938.15** | protein coding | *CLEC4M* | 0.98 | 0.80 | 0.12 | 2.53 | -2.41 | 0.40 | 0.73 | 8.23E-19 |
| **ENSG00000130300.7** | protein coding | *PLVAP* | 0.97 | 3.81 | 5.24 | 2.17 | 3.07 | 0.94 | 0.60 | 2.60E-34 |
| **ENSG00000165682.13** | protein coding | *CLEC1B* | 0.97 | 1.20 | 0.13 | 2.20 | -2.07 | 0.38 | 0.58 | 2.55E-20 |
| **ENSG00000184374.2** | protein coding | *COLEC10* | 0.97 | 2.60 | 0.71 | 3.63 | -2.92 | 0.86 | 0.43 | 2.56E-48 |
| **ENSG00000160339.14** | protein coding | *FCN2* | 0.97 | 3.90 | 0.87 | 5.30 | -4.44 | 1.15 | 0.71 | 1.79E-40 |
| **ENSG00000185633.9** | protein coding | *NDUFA4L2* | 0.96 | 1.49 | 2.90 | 0.88 | 2.02 | 1.19 | 0.25 | 9.06E-71 |
| **ENSG00000160801.12** | protein coding | *PTH1R* | 0.96 | 1.80 | 0.82 | 2.90 | -2.07 | 0.68 | 0.49 | 2.58E-28 |
| **ENSG00000143369.13** | protein coding | *ECM1* | 0.96 | 3.02 | 1.46 | 3.76 | -2.30 | 0.91 | 0.38 | 7.31E-50 |
| **ENSG00000263761.2** | protein coding | *GDF2* | 0.96 | 1.20 | 0.22 | 2.79 | -2.58 | 0.59 | 0.62 | 1.04E-24 |
| **ENSG00000138315.11** | protein coding | *OIT3* | 0.96 | 4.03 | 2.17 | 4.80 | -2.63 | 0.96 | 0.64 | 6.42E-28 |
| **ENSG00000145708.9** | protein coding | *CRHBP* | 0.96 | 3.10 | 0.68 | 4.25 | -3.57 | 0.93 | 0.77 | 7.31E-29 |
| **ENSG00000019169.10** | protein coding | *MARCO* | 0.96 | 2.90 | 0.74 | 4.87 | -4.13 | 1.06 | 1.05 | 6.48E-24 |
| **ENSG00000077152.8** | protein coding | *UBE2T* | 0.95 | 1.04 | 2.96 | 0.60 | 2.36 | 1.08 | 0.36 | 3.28E-59 |
| **ENSG00000134057.13** | protein coding | *CCNB1* | 0.95 | 1.06 | 2.87 | 0.60 | 2.27 | 1.21 | 0.26 | 2.31E-78 |
| **ENSG00000126759.11** | protein coding | *CFP* | 0.95 | 1.88 | 0.73 | 2.78 | -2.05 | 0.61 | 0.62 | 1.50E-20 |
| **ENSG00000145824.11** | protein coding | *CXCL14* | 0.95 | 2.80 | 0.76 | 3.86 | -3.10 | 1.19 | 0.62 | 8.03E-37 |
| **ENSG00000101057.14** | protein coding | *MYBL2* | 0.94 | 0.57 | 2.48 | 0.26 | 2.22 | 1.54 | 0.16 | 9.91E-70 |
| **ENSG00000131747.13** | protein coding | *TOP2A* | 0.94 | 0.64 | 2.51 | 0.31 | 2.20 | 1.31 | 0.22 | 1.09E-77 |
| **ENSG00000112312.8** | protein coding | *GMNN* | 0.94 | 2.06 | 3.79 | 1.62 | 2.17 | 1.06 | 0.37 | 4.74E-52 |
| **ENSG00000142748.11** | protein coding | *FCN3* | 0.94 | 4.58 | 1.38 | 5.45 | -4.06 | 1.30 | 0.85 | 2.13E-32 |
| **ENSG00000175063.15** | protein coding | *UBE2C* | 0.93 | 0.95 | 3.06 | 0.45 | 2.61 | 1.45 | 0.32 | 2.27E-73 |
| **ENSG00000199753.1** | snoRNA | *SNORD104* | 0.93 | 2.90 | 4.42 | 1.85 | 2.57 | 1.36 | 0.73 | 5.65E-26 |
| **ENSG00000117399.12** | protein coding | *CDC20* | 0.93 | 0.85 | 2.75 | 0.34 | 2.41 | 1.43 | 0.24 | 5.49E-79 |
| **ENSG00000164611.11** | protein coding | *PTTG1* | 0.93 | 0.91 | 2.79 | 0.47 | 2.32 | 1.28 | 0.29 | 3.57E-73 |
| **ENSG00000089685.13** | protein coding | *BIRC5* | 0.93 | 0.82 | 2.44 | 0.29 | 2.15 | 1.28 | 0.21 | 7.83E-79 |
| **ENSG00000107562.15** | protein coding | *CXCL12* | 0.93 | 3.88 | 2.38 | 4.85 | -2.46 | 1.35 | 0.51 | 4.63E-41 |
| **ENSG00000249173.4** | lincRNA | *LINC01093* | 0.93 | 3.50 | 1.27 | 4.85 | -3.58 | 1.45 | 0.78 | 3.79E-34 |
| **ENSG00000109576.12** | protein coding | *AADAT* | 0.92 | 3.11 | 1.46 | 3.54 | -2.08 | 0.90 | 0.49 | 1.91E-31 |
| **ENSG00000213398.6** | protein coding | *LCAT* | 0.92 | 5.18 | 3.50 | 6.08 | -2.58 | 1.39 | 0.43 | 1.72E-53 |
| **ENSG00000175336.9** | protein coding | *APOF* | 0.92 | 6.12 | 3.96 | 7.44 | -3.48 | 2.05 | 0.56 | 1.67E-56 |
| **ENSG00000140107.10** | protein coding | *SLC25A47* | 0.92 | 6.91 | 3.82 | 7.88 | -4.06 | 2.63 | 0.48 | 5.17E-68 |
| **ENSG00000105697.6** | protein coding | *HAMP* | 0.92 | 5.00 | 1.81 | 6.77 | -4.96 | 2.09 | 1.38 | 9.30E-25 |
| **ENSG00000140505.6** | protein coding | *CYP1A2* | 0.92 | 5.00 | 1.96 | 7.13 | -5.18 | 2.38 | 1.11 | 1.54E-36 |
| **ENSG00000101412.12** | protein coding | *E2F1* | 0.91 | 0.64 | 2.74 | 0.42 | 2.32 | 1.28 | 0.36 | 8.41E-60 |
| **ENSG00000167900.10** | protein coding | *TK1* | 0.91 | 2.33 | 3.93 | 1.62 | 2.31 | 1.23 | 0.51 | 1.63E-37 |
| **ENSG00000154096.12** | protein coding | *THY1* | 0.91 | 1.45 | 2.92 | 0.89 | 2.03 | 0.99 | 0.58 | 3.49E-25 |
| **ENSG00000196139.10** | protein coding | *AKR1C3* | 0.91 | 5.16 | 6.08 | 4.07 | 2.01 | 1.05 | 0.46 | 9.62E-35 |
| **ENSG00000234741.6** | processed_transcript | *GAS5* | 0.91 | 3.64 | 4.67 | 2.66 | 2.00 | 1.10 | 0.51 | 1.30E-30 |
| **ENSG00000120057.4** | protein coding | *SFRP5* | 0.91 | 1.40 | 0.82 | 3.03 | -2.21 | 1.63 | 0.64 | 7.33E-27 |
| **ENSG00000146233.6** | protein coding | *CYP39A1* | 0.91 | 3.50 | 1.78 | 4.13 | -2.35 | 1.40 | 0.39 | 8.27E-55 |
| **ENSG00000163687.12** | protein coding | *DNASE1L3* | 0.91 | 3.55 | 2.00 | 4.60 | -2.60 | 1.19 | 0.68 | 6.42E-28 |
| **ENSG00000156006.4** | protein coding | *NAT2* | 0.91 | 3.40 | 1.81 | 4.58 | -2.76 | 1.47 | 0.58 | 5.42E-40 |
| **ENSG00000138115.12** | protein coding | *CYP2C8* | 0.91 | 7.94 | 5.35 | 8.73 | -3.37 | 2.38 | 0.51 | 8.97E-56 |
| **ENSG00000172425.9** | protein coding | *TTC36* | 0.91 | 4.80 | 2.30 | 5.85 | -3.55 | 1.97 | 0.56 | 2.01E-57 |
| **ENSG00000113600.9** | protein coding | *C9* | 0.91 | 7.60 | 4.16 | 8.52 | -4.36 | 2.96 | 0.57 | 4.92E-62 |
| **ENSG00000171848.12** | protein coding | *RRM2* | 0.90 | 0.98 | 2.47 | 0.46 | 2.02 | 1.11 | 0.38 | 1.10E-46 |
| **ENSG00000186910.3** | protein coding | *SERPINA11* | 0.90 | 7.12 | 5.59 | 8.00 | -2.41 | 1.98 | 0.41 | 1.06E-45 |
| **ENSG00000187048.11** | protein coding | *CYP4A11* | 0.90 | 7.21 | 5.30 | 7.86 | -2.56 | 1.94 | 0.43 | 2.18E-49 |
| **ENSG00000125740.12** | protein coding | *FOSB* | 0.90 | 2.54 | 1.37 | 4.18 | -2.81 | 1.26 | 1.33 | 3.09E-12 |
| **ENSG00000187193.8** | protein coding | *MT1X* | 0.90 | 6.97 | 4.58 | 8.22 | -3.64 | 2.16 | 1.24 | 9.88E-21 |
| **ENSG00000198417.6** | protein coding | *MT1F* | 0.90 | 3.71 | 2.05 | 6.01 | -3.96 | 1.82 | 1.47 | 3.00E-17 |
| **ENSG00000125144.12** | protein coding | *MT1G* | 0.90 | 7.33 | 4.13 | 9.67 | -5.54 | 2.95 | 1.77 | 5.69E-22 |
| **ENSG00000147257.12** | protein coding | *GPC3* | 0.89 | 3.76 | 6.34 | 1.46 | 4.88 | 2.86 | 0.89 | 9.97E-49 |
| **ENSG00000110492.14** | protein coding | *MDK* | 0.89 | 3.08 | 5.35 | 1.88 | 3.47 | 1.88 | 0.93 | 2.20E-28 |
| **ENSG00000087237.9** | protein coding | *CETP* | 0.89 | 2.02 | 1.50 | 3.53 | -2.03 | 0.90 | 0.90 | 1.90E-13 |
| **ENSG00000147003.5** | protein coding | *TMEM27* | 0.89 | 2.52 | 1.39 | 3.43 | -2.04 | 1.17 | 0.54 | 1.64E-29 |
| **ENSG00000166840.12** | protein coding | *GLYATL1* | 0.89 | 4.70 | 3.23 | 5.34 | -2.12 | 1.56 | 0.32 | 5.09E-54 |
| **ENSG00000039537.12** | protein coding | *C6* | 0.89 | 6.28 | 4.84 | 7.04 | -2.20 | 1.77 | 0.37 | 9.15E-47 |
| **ENSG00000123454.9** | protein coding | *DBH* | 0.89 | 2.43 | 1.14 | 3.34 | -2.20 | 0.90 | 0.89 | 4.72E-15 |
| **ENSG00000165140.8** | protein coding | *FBP1* | 0.89 | 7.75 | 6.25 | 8.53 | -2.28 | 1.76 | 0.45 | 1.01E-42 |
| **ENSG00000011465.15** | protein coding | *DCN* | 0.89 | 3.40 | 1.77 | 4.11 | -2.34 | 1.50 | 0.53 | 2.78E-37 |
| **ENSG00000073754.5** | protein coding | *CD5L* | 0.89 | 3.00 | 1.50 | 4.43 | -2.93 | 1.47 | 1.00 | 8.80E-20 |
| **ENSG00000162840.4** | processed pseudogene | *MT2P1* | 0.89 | 2.40 | 1.57 | 4.67 | -3.10 | 1.28 | 1.30 | 2.42E-14 |
| **ENSG00000169715.13** | protein coding | *MT1E* | 0.89 | 6.62 | 4.43 | 8.58 | -4.15 | 2.49 | 0.99 | 1.99E-34 |
| **ENSG00000205358.3** | protein coding | *MT1H* | 0.89 | 2.50 | 1.51 | 6.45 | -4.94 | 2.01 | 2.11 | 5.53E-14 |
| **ENSG00000233929.1** | processed pseudogene | *MT1XP1* | 0.88 | 1.40 | 0.82 | 2.94 | -2.12 | 0.85 | 1.08 | 6.30E-11 |
| **ENSG00000160932.9** | protein coding | *LY6E* | 0.88 | 6.07 | 4.50 | 6.95 | -2.45 | 1.93 | 0.51 | 6.20E-40 |
| **ENSG00000111713.2** | protein coding | *GYS2* | 0.88 | 4.61 | 2.85 | 5.31 | -2.45 | 1.79 | 0.41 | 1.91E-50 |
| **ENSG00000101981.9** | protein coding | *F9* | 0.88 | 6.70 | 4.88 | 7.38 | -2.50 | 2.27 | 0.34 | 9.42E-45 |
| **ENSG00000197408.7** | protein coding | *CYP2B6* | 0.88 | 5.30 | 3.69 | 6.59 | -2.90 | 1.90 | 1.03 | 7.23E-20 |
| **ENSG00000125148.6** | protein coding | *MT2A* | 0.88 | 8.20 | 6.92 | 10.16 | -3.24 | 1.88 | 1.02 | 4.74E-23 |
| **ENSG00000205364.3** | protein coding | *MT1M* | 0.88 | 4.10 | 1.77 | 5.95 | -4.17 | 1.95 | 1.65 | 7.11E-16 |
| **ENSG00000176907.4** | protein coding | *C8orf4* | 0.87 | 4.88 | 3.73 | 5.81 | -2.08 | 1.31 | 0.63 | 4.42E-25 |
| **ENSG00000257017.7** | protein coding | *HP* | 0.87 | 10.94 | 9.10 | 11.79 | -2.69 | 2.38 | 0.52 | 1.67E-39 |
| **ENSG00000159650.7** | protein coding | *UROC1* | 0.87 | 4.40 | 2.43 | 5.23 | -2.80 | 1.98 | 0.68 | 1.32E-34 |
| **ENSG00000116882.13** | protein coding | *HAO2* | 0.87 | 5.80 | 3.08 | 6.41 | -3.33 | 2.28 | 0.56 | 6.66E-52 |
| **ENSG00000175003.11** | protein coding | *SLC22A1* | 0.87 | 6.94 | 4.35 | 8.09 | -3.74 | 2.73 | 0.74 | 5.26E-43 |
| **ENSG00000170509.10** | protein coding | *HSD17B13* | 0.87 | 6.20 | 3.34 | 7.36 | -4.02 | 2.84 | 0.51 | 9.67E-61 |
| **ENSG00000160868.13** | protein coding | *CYP3A4* | 0.87 | 7.60 | 4.72 | 9.28 | -4.57 | 3.54 | 0.95 | 4.18E-40 |
| **ENSG00000150656.13** | protein coding | *CNDP1* | 0.86 | 1.40 | 0.62 | 2.63 | -2.00 | 0.90 | 0.98 | 1.26E-11 |
| **ENSG00000112964.12** | protein coding | *GHR* | 0.86 | 3.79 | 2.57 | 4.65 | -2.08 | 1.25 | 0.78 | 6.74E-18 |
| **ENSG00000266964.4** | protein coding | *FXYD1* | 0.86 | 3.90 | 2.36 | 4.48 | -2.12 | 1.70 | 0.44 | 4.92E-40 |
| **ENSG00000171234.12** | protein coding | *UGT2B7* | 0.86 | 7.42 | 5.76 | 8.09 | -2.33 | 2.06 | 0.42 | 1.75E-41 |
| **ENSG00000249948.5** | polymorphic_pseudogene | *GBA3* | 0.86 | 4.00 | 2.50 | 4.85 | -2.35 | 1.62 | 0.61 | 1.74E-31 |
| **ENSG00000138109.9** | protein coding | *CYP2C9* | 0.86 | 7.42 | 5.73 | 8.10 | -2.37 | 2.22 | 0.46 | 5.41E-37 |
| **ENSG00000099769.5** | protein coding | *IGFALS* | 0.86 | 4.10 | 2.25 | 5.60 | -3.35 | 1.84 | 1.13 | 1.06E-20 |
| **ENSG00000198099.7** | protein coding | *ADH4* | 0.86 | 8.51 | 6.08 | 9.52 | -3.44 | 2.90 | 0.68 | 3.17E-40 |
| **ENSG00000213886.3** | protein coding | *UBD* | 0.85 | 3.20 | 5.26 | 2.38 | 2.88 | 1.85 | 1.48 | 3.39E-11 |
| **ENSG00000162365.10** | protein coding | *CYP4A22* | 0.85 | 4.70 | 3.60 | 5.83 | -2.23 | 1.64 | 0.61 | 4.02E-29 |
| **ENSG00000166391.13** | protein coding | *MOGAT2* | 0.85 | 3.40 | 2.03 | 4.29 | -2.26 | 1.57 | 0.64 | 4.88E-28 |
| **ENSG00000148965.7** | protein coding | *SAA4* | 0.85 | 7.60 | 5.81 | 8.11 | -2.30 | 2.13 | 0.55 | 3.99E-32 |
| **ENSG00000126838.8** | protein coding | *PZP* | 0.85 | 0.41 | 0.45 | 2.84 | -2.38 | 0.89 | 1.77 | 1.04E-05 |
| **ENSG00000196616.11** | protein coding | *ADH1B* | 0.85 | 8.12 | 6.53 | 8.98 | -2.45 | 2.31 | 0.50 | 9.49E-36 |
| **ENSG00000149124.9** | protein coding | *GLYAT* | 0.85 | 5.10 | 3.31 | 5.84 | -2.53 | 2.11 | 0.52 | 2.16E-39 |
| **ENSG00000139547.7** | protein coding | *RDH16* | 0.85 | 6.35 | 4.27 | 6.81 | -2.54 | 2.09 | 0.63 | 1.55E-32 |
| **ENSG00000170345.8** | protein coding | *FOS* | 0.85 | 4.67 | 3.49 | 6.38 | -2.89 | 1.56 | 1.36 | 1.38E-12 |
| **ENSG00000215644.8** | protein coding | *GCGR* | 0.84 | 3.60 | 2.28 | 4.33 | -2.05 | 1.94 | 0.40 | 1.73E-36 |
| **ENSG00000112936.17** | protein coding | *C7* | 0.84 | 3.80 | 2.45 | 5.10 | -2.65 | 1.97 | 0.94 | 3.09E-20 |
| **ENSG00000167244.16** | protein coding | *IGF2* | 0.84 | 6.07 | 4.28 | 7.08 | -2.80 | 3.62 | 0.62 | 2.92E-23 |
| **ENSG00000124253.10** | protein coding | *PCK1* | 0.84 | 7.51 | 5.23 | 8.11 | -2.88 | 2.44 | 0.68 | 3.25E-34 |
| **ENSG00000183549.9** | protein coding | *ACSM5* | 0.83 | 4.90 | 3.57 | 5.60 | -2.04 | 1.75 | 0.59 | 1.04E-26 |
| **ENSG00000205866.3** | lincRNA | *FAM99A* | 0.83 | 2.20 | 1.60 | 3.72 | -2.13 | 1.68 | 0.69 | 2.68E-23 |
| **ENSG00000111700.11** | protein coding | *SLCO1B3* | 0.83 | 2.70 | 1.49 | 3.72 | -2.23 | 1.69 | 0.84 | 1.65E-18 |
| **ENSG00000072080.9** | protein coding | *SPP2* | 0.83 | 6.10 | 4.70 | 7.02 | -2.32 | 2.33 | 0.68 | 6.03E-25 |
| **ENSG00000260549.1** | unitary_pseudogene | *MT1L* | 0.83 | 3.60 | 2.45 | 4.86 | -2.41 | 1.74 | 1.26 | 5.64E-11 |
| **ENSG00000120738.7** | protein coding | *EGR1* | 0.83 | 5.85 | 4.28 | 6.71 | -2.43 | 1.50 | 1.12 | 3.53E-13 |
| **ENSG00000248144.4** | protein coding | *ADH1C* | 0.83 | 8.12 | 6.23 | 8.79 | -2.56 | 2.74 | 0.46 | 3.89E-33 |
| **ENSG00000168389.16** | protein coding | *MFSD2A* | 0.83 | 3.84 | 2.14 | 4.84 | -2.70 | 1.85 | 1.05 | 1.97E-17 |
| **ENSG00000109758.7** | protein coding | *HGFAC* | 0.82 | 5.31 | 3.09 | 5.88 | -2.79 | 2.44 | 0.75 | 3.63E-29 |
| **ENSG00000166741.6** | protein coding | *NNMT* | 0.82 | 7.86 | 5.44 | 8.54 | -3.10 | 2.44 | 1.01 | 5.67E-23 |
| **ENSG00000248709.1** | lincRNA | *CTC-505O3.2* | 0.81 | 3.60 | 2.49 | 4.50 | -2.01 | 1.80 | 0.72 | 5.75E-20 |
| **ENSG00000161031.11** | protein coding | *PGLYRP2* | 0.81 | 5.51 | 4.33 | 6.35 | -2.01 | 2.04 | 0.58 | 3.64E-25 |
| **ENSG00000079557.4** | protein coding | *AFM* | 0.81 | 6.50 | 5.13 | 7.19 | -2.07 | 1.93 | 0.61 | 1.08E-25 |
| **ENSG00000129214.13** | protein coding | *SHBG* | 0.81 | 3.78 | 2.75 | 4.83 | -2.08 | 1.73 | 0.93 | 2.81E-14 |
| **ENSG00000151790.7** | protein coding | *TDO2* | 0.81 | 5.44 | 4.07 | 6.34 | -2.27 | 2.11 | 0.56 | 7.54E-31 |
| **ENSG00000117594.8** | protein coding | *HSD11B1* | 0.81 | 7.24 | 5.66 | 7.97 | -2.31 | 2.91 | 0.35 | 5.69E-27 |
| **ENSG00000237949.1** | lincRNA | *LINC00844* | 0.81 | 3.80 | 2.66 | 4.98 | -2.32 | 2.29 | 0.99 | 2.86E-15 |
| **ENSG00000100652.4** | protein coding | *SLC10A1* | 0.81 | 6.53 | 4.56 | 6.94 | -2.37 | 2.54 | 0.54 | 1.84E-29 |
| **ENSG00000180432.5** | protein coding | *CYP8B1* | 0.81 | 6.01 | 4.43 | 7.00 | -2.57 | 2.67 | 0.46 | 2.46E-34 |
| **ENSG00000175793.11** | protein coding | *SFN* | 0.80 | 1.00 | 3.32 | 0.74 | 2.58 | 2.17 | 0.85 | 1.79E-22 |
| **ENSG00000181019.11** | protein coding | *NQO1* | 0.80 | 1.00 | 3.46 | 0.89 | 2.57 | 2.72 | 0.83 | 9.05E-22 |
| **ENSG00000184999.10** | protein coding | *SLC22A10* | 0.80 | 2.60 | 2.09 | 4.09 | -2.01 | 1.40 | 1.14 | 1.24E-09 |
| **ENSG00000166148.3** | protein coding | *AVPR1A* | 0.80 | 1.90 | 1.16 | 3.22 | -2.06 | 1.37 | 1.39 | 4.97E-07 |
| **ENSG00000136872.16** | protein coding | *ALDOB* | 0.80 | 10.65 | 9.25 | 11.34 | -2.10 | 2.46 | 0.52 | 5.90E-25 |
| **ENSG00000122787.13** | protein coding | *AKR1D1* | 0.80 | 4.60 | 3.15 | 5.50 | -2.35 | 1.97 | 0.77 | 8.19E-23 |
| **ENSG00000211895.4** | IG_C_gene | *IGHA1* | 0.80 | 6.23 | 4.30 | 7.09 | -2.78 | 2.42 | 1.54 | 4.74E-10 |
| **ENSG00000151365.2** | protein coding | *THRSP* | 0.80 | 4.30 | 3.14 | 6.20 | -3.06 | 2.71 | 1.08 | 2.21E-20 |
| **ENSG00000068366.18** | protein coding | *ACSL4* | 0.79 | 3.22 | 4.36 | 2.06 | 2.29 | 2.07 | 1.21 | 6.98E-11 |
| **ENSG00000132465.9** | protein coding | *IGJ* | 0.79 | 2.70 | 2.09 | 4.15 | -2.06 | 1.83 | 1.28 | 3.16E-08 |
| **ENSG00000130649.8** | protein coding | *CYP2E1* | 0.79 | 7.93 | 5.62 | 8.86 | -3.23 | 3.31 | 0.86 | 4.05E-27 |
| **ENSG00000118785.12** | protein coding | *SPP1* | 0.78 | 4.16 | 5.55 | 3.02 | 2.53 | 3.21 | 1.09 | 1.28E-13 |
| **ENSG00000173702.6** | protein coding | *MUC13* | 0.78 | 1.50 | 2.99 | 0.49 | 2.50 | 2.60 | 0.66 | 6.30E-27 |
| **ENSG00000237988.3** | unprocessed pseudogene | *OR2I1P* | 0.78 | 3.73 | 4.94 | 2.55 | 2.39 | 1.99 | 1.60 | 3.84E-07 |
| **ENSG00000198077.9** | protein coding | *CYP2A7* | 0.78 | 2.30 | 2.10 | 4.26 | -2.16 | 2.36 | 1.57 | 7.67E-06 |
| **ENSG00000211899.6** | IG_C_gene | *IGHM* | 0.78 | 3.30 | 2.79 | 5.00 | -2.22 | 2.07 | 1.36 | 2.62E-08 |
| **ENSG00000145692.13** | protein coding | *BHMT* | 0.78 | 6.47 | 5.02 | 7.39 | -2.37 | 2.31 | 0.79 | 8.54E-22 |
| **ENSG00000255974.5** | protein coding | *CYP2A6* | 0.78 | 7.51 | 5.37 | 8.69 | -3.32 | 3.46 | 1.15 | 3.47E-20 |
| **ENSG00000198074.8** | protein coding | *AKR1B10* | 0.77 | 4.70 | 6.03 | 2.39 | 3.64 | 3.38 | 1.92 | 5.87E-11 |
| **ENSG00000148346.10** | protein coding | *LCN2* | 0.77 | 2.87 | 4.37 | 1.80 | 2.57 | 2.79 | 1.08 | 3.23E-15 |
| **ENSG00000106366.8** | protein coding | *SERPINE1* | 0.77 | 5.45 | 4.46 | 6.60 | -2.14 | 1.74 | 1.37 | 1.06E-07 |
| **ENSG00000198650.9** | protein coding | *TAT* | 0.77 | 6.95 | 5.54 | 7.87 | -2.34 | 2.78 | 0.68 | 5.12E-22 |
| **ENSG00000124713.5** | protein coding | *GNMT* | 0.75 | 5.11 | 4.67 | 6.76 | -2.09 | 2.26 | 1.08 | 4.24E-11 |
| **ENSG00000163993.6** | protein coding | *S100P* | 0.74 | 0.80 | 2.72 | 0.58 | 2.14 | 2.80 | 0.45 | 2.03E-23 |
| **ENSG00000101210.9** | protein coding | *EEF1A2* | 0.73 | 1.30 | 3.33 | 0.86 | 2.47 | 2.82 | 1.22 | 1.06E-11 |
| **ENSG00000135094.9** | protein coding | *SDS* | 0.73 | 4.28 | 4.88 | 7.10 | -2.23 | 3.06 | 1.33 | 4.91E-08 |
| **ENSG00000172016.14** | protein coding | *REG3A* | 0.72 | 0.20 | 2.12 | 0.10 | 2.02 | 3.41 | 0.32 | 8.12E-16 |

**Table K. Top 53 RNA transcripts (with AUC = or > 0.9) based on Single gene threshold based approach for cancer versus normal classification.**

| **Ensemble ID** | **AUC** | **Threshold (log2(FPKM))** | **Mean in cancer (log2(FPKM))** | **Mean in Norma**  **l(log2(FPKM))** | **Gene Symbol** | **Class** | **Regulation** |
| --- | --- | --- | --- | --- | --- | --- | --- |
| **ENSG00000182566.11** | 0.99 | 2.90 | 0.46 | 4.83 | *CLEC4G* | protein coding | Downregulated |
| **ENSG00000104938.15** | 0.98 | 0.80 | 0.12 | 2.53 | *CLEC4M* | protein coding | Downregulated |
| **ENSG00000160339.14** | 0.97 | 3.90 | 0.87 | 5.30 | *FCN2* | protein coding | Downregulated |
| **ENSG00000184374.2** | 0.97 | 2.60 | 0.71 | 3.63 | *COLEC10* | protein coding | Downregulated |
| **ENSG00000165682.13** | 0.97 | 1.20 | 0.13 | 2.20 | *CLEC1B* | protein coding | Downregulated |
| **ENSG00000130300.7** | 0.97 | 3.81 | 5.24 | 2.17 | *PLVAP* | protein coding | Upregulated |
| **ENSG00000019169.10** | 0.96 | 2.90 | 0.74 | 4.87 | *MARCO* | protein coding | Downregulated |
| **ENSG00000145708.9** | 0.96 | 3.10 | 0.68 | 4.25 | *CRHBP* | protein coding | Downregulated |
| **ENSG00000138315.11** | 0.96 | 4.03 | 2.17 | 4.80 | *OIT3* | protein coding | Downregulated |
| **ENSG00000263761.2** | 0.96 | 1.20 | 0.22 | 2.79 | *GDF2* | protein coding | Downregulated |
| **ENSG00000143369.13** | 0.96 | 3.02 | 1.46 | 3.76 | *ECM1* | protein coding | Downregulated |
| **ENSG00000160801.12** | 0.96 | 1.80 | 0.82 | 2.90 | *PTH1R* | protein coding | Downregulated |
| **ENSG00000185633.9** | 0.96 | 1.49 | 2.90 | 0.88 | *NDUFA4L2* | protein coding | Upregulated |
| **ENSG00000145824.11** | 0.95 | 2.80 | 0.76 | 3.86 | *CXCL14* | protein coding | Downregulated |
| **ENSG00000126759.11** | 0.95 | 1.88 | 0.73 | 2.78 | *CFP* | protein coding | Downregulated |
| **ENSG00000134057.13** | 0.95 | 1.06 | 2.87 | 0.60 | *CCNB1* | protein coding | Upregulated |
| **ENSG00000077152.8** | 0.95 | 1.04 | 2.96 | 0.60 | *UBE2T* | protein coding | Upregulated |
| **ENSG00000142748.11** | 0.94 | 4.58 | 1.38 | 5.45 | *FCN3* | protein coding | Downregulated |
| **ENSG00000112312.8** | 0.94 | 2.06 | 3.79 | 1.62 | *GMNN* | protein coding | Upregulated |
| **ENSG00000131747.13** | 0.94 | 0.64 | 2.51 | 0.31 | *TOP2A* | protein coding | Upregulated |
| **ENSG00000101057.14** | 0.94 | 0.57 | 2.48 | 0.26 | *MYBL2* | protein coding | Upregulated |
| **ENSG00000249173.4** | 0.93 | 3.50 | 1.27 | 4.85 | *LINC01093* | lincRNA | Downregulated |
| **ENSG00000107562.15** | 0.93 | 3.88 | 2.38 | 4.85 | *CXCL12* | protein coding | Downregulated |
| **ENSG00000089685.13** | 0.93 | 0.82 | 2.44 | 0.29 | *BIRC5* | protein coding | Upregulated |
| **ENSG00000164611.11** | 0.93 | 0.91 | 2.79 | 0.47 | *PTTG1* | protein coding | Upregulated |
| **ENSG00000117399.12** | 0.93 | 0.85 | 2.75 | 0.34 | *CDC20* | protein coding | Upregulated |
| **ENSG00000199753.1** | 0.93 | 2.90 | 4.42 | 1.85 | *SNORD104* | snoRNA | Upregulated |
| **ENSG00000175063.15** | 0.93 | 0.95 | 3.06 | 0.45 | *UBE2C* | protein coding | Upregulated |
| **ENSG00000140505.6** | 0.92 | 5.00 | 1.96 | 7.13 | *CYP1A2* | protein coding | Downregulated |
| **ENSG00000105697.6** | 0.92 | 5.00 | 1.81 | 6.77 | *HAMP* | protein coding | Downregulated |
| **ENSG00000140107.10** | 0.92 | 6.91 | 3.82 | 7.88 | *SLC25A47* | protein coding | Downregulated |
| **ENSG00000175336.9** | 0.92 | 6.12 | 3.96 | 7.44 | *APOF* | protein coding | Downregulated |
| **ENSG00000213398.6** | 0.92 | 5.18 | 3.50 | 6.08 | *LCAT* | protein coding | Downregulated |
| **ENSG00000109576.12** | 0.92 | 3.11 | 1.46 | 3.54 | *AADAT* | protein coding | Downregulated |
| **ENSG00000113600.9** | 0.91 | 7.60 | 4.16 | 8.52 | *C9* | protein coding | Downregulated |
| **ENSG00000172425.9** | 0.91 | 4.80 | 2.30 | 5.85 | *TTC36* | protein coding | Downregulated |
| **ENSG00000138115.12** | 0.91 | 7.94 | 5.35 | 8.73 | *CYP2C8* | protein coding | Downregulated |
| **ENSG00000156006.4** | 0.91 | 3.40 | 1.81 | 4.58 | *NAT2* | protein coding | Downregulated |
| **ENSG00000163687.12** | 0.91 | 3.55 | 2.00 | 4.60 | *DNASE1L3* | protein coding | Downregulated |
| **ENSG00000146233.6** | 0.91 | 3.50 | 1.78 | 4.13 | *CYP39A1* | protein coding | Downregulated |
| **ENSG00000120057.4** | 0.91 | 1.40 | 0.82 | 3.03 | *SFRP5* | protein coding | Downregulated |
| **ENSG00000234741.6** | 0.91 | 3.64 | 4.67 | 2.66 | *GAS5* | processed_transcript | Upregulated |
| **ENSG00000196139.10** | 0.91 | 5.16 | 6.08 | 4.07 | *AKR1C3* | protein coding | Upregulated |
| **ENSG00000154096.12** | 0.91 | 1.45 | 2.92 | 0.89 | *THY1* | protein coding | Upregulated |
| **ENSG00000167900.10** | 0.91 | 2.33 | 3.93 | 1.62 | *TK1* | protein coding | Upregulated |
| **ENSG00000101412.12** | 0.91 | 0.64 | 2.74 | 0.42 | *E2F1* | protein coding | Upregulated |
| **ENSG00000125144.12** | 0.90 | 7.33 | 4.13 | 9.67 | *MT1G* | protein coding | Downregulated |
| **ENSG00000198417.6** | 0.90 | 3.71 | 2.05 | 6.01 | *MT1F* | protein coding | Downregulated |
| **ENSG00000187193.8** | 0.90 | 6.97 | 4.58 | 8.22 | *MT1X* | protein coding | Downregulated |
| **ENSG00000125740.12** | 0.90 | 2.54 | 1.37 | 4.18 | *FOSB* | protein coding | Downregulated |
| **ENSG00000187048.11** | 0.90 | 7.21 | 5.30 | 7.86 | *CYP4A11* | protein coding | Downregulated |
| **ENSG00000186910.3** | 0.90 | 7.12 | 5.59 | 8.00 | *SERPINA11* | protein coding | Downregulated |
| **ENSG00000171848.12** | 0.90 | 0.98 | 2.47 | 0.46 | *RRM2* | protein coding | Upregulated |

**Table L-A. Functional importance of 10 signatures (LCN-RNA-AUC and LCN-5RNA) and their implication in liver cancer or other malignancies.**

| **RNA transcript** | **Type of transcript** | **Regulation in late stage** | **GO terms associated with Gene** | **Function** | **Reported in Previous Literature** | **Reported in case of**  **Liver cancer** |
| --- | --- | --- | --- | --- | --- | --- |
| *CLEC4G* | Protein Coding | Underexpressed in Cancer | regulation of immune response (GO:0050776) | It encodes C-type lectin domain family 4 member G, that binds to mannose, N-acetylglucosamine (GlcNAc) and fucose in a Ca2+-dependent manner. It act as receptor for various viruses like Evola,SARS etc ^1,53–55^ . | YES | YES^56^ |
| *CLEC4M* | Protein Coding | Underexpressed in Cancer | modification by symbiont of host morphology or physiology (GO:0044003); virion attachment to host cell (GO:0019062); modulation by virus of host morphology or physiology (GO:0019048); leukocyte cell-cell adhesion (GO:0007159); peptide transport (GO:0015833); viral genome replication (GO:0019079); integral component of plasma membrane (GO:0005887) | It encodes C-type lectin domain family 4 member M, a pathogen-recognition receptor that involved in peripheral immunity in liver. It stimulates the endocytosis of pathogens that are further degraded in lysosomes ^1,53–55^ . | YES | YES^47^ |
| *PLVAP* | Protein Coding | Overexpressed in Cancer | caveola (GO:0005901); positive regulation of cellular extravasation (GO:0002693); positive regulation of leukocyte migration (GO:0002687); cellular response to tumor necrosis factor (GO:0071356); MAPK cascade (GO:0000165); cytokine-mediated signaling pathway (GO:0019221) | It encodes Plasmalemma vesicle-associated protein, which is involved in the formation of stomatal and fenestral diaphragms of caveolae. Further it may involved in microvascular permeability ^1,57^. | YES | YES^58^ |
| *CLEC1B* | Protein Coding | Underexpressed in Cancer | NA | It encodes C-type lectin domain family 1 member B, which acts as a platelet receptor for the lymphatic endothelial marker, PDPN. After ligand activation, signals via sequential activation of SRC and SYK tyrosine kinases leading to activation of PLCG2 ^58^ | YES | YES ^47,56^ |
| *COLEC10* | Protein Coding | Underexpressed in Cancer | mannose binding (GO:0005537); serine-type peptidase activity (GO:0008236); complement activation, lectin pathway (GO:0001867); | It encodes Collectin-10 and which binds to various carbohydrates like galactose, mannose, fucose, N-acetylglucosamine and N-acetylgalactosamine. It also acts as chemoattractant and may regualte cell migration ^1,59,60^ . | YES | YES ^61^ |
| *FCN2* | Protein  coding | Underexpressed in Cancer | serine-type peptidase activity (GO:0008236); endopeptidase activity (GO:0004175); complement activation, lectin pathway (GO:0001867); apoptotic cell clearance (GO:0043277); defense response to Gram-positive bacterium (GO:0050830); | It encodes Ficolin-2 , which function in innate immunity through activation of the lectin complement pathway. Calcium-dependent and GlcNAc-binding lectin. Enhances phagocytosis of S.typhimurium by neutrophils, suggesting an opsonic effect via the collagen region ^61^ . | YES | YES^47,62^ |
| *NDUFA4L2* | Protein Coding | Underexpressed in Cancer | mitochondrial respiratory chain complex IV (GO:0005751); | It encodes NADH dehydrogenase [ubiquinone] 1 alpha subcomplex subunit 4-like 2 that participate in electron transport chain ^1^. | YES^63^ | NO |
| *PTH1R* | Protein Coding | Underexpressed in Cancer | protein homodimerization activity (GO:0042803); G-protein coupled receptor activity (GO:0004930); integral component of plasma membrane (GO:0005887); adenylate cyclase-activating G-protein coupled receptor signaling pathway (GO:0007189); cAMP-mediated signaling (GO:0019933); positive regulation of cellular biosynthetic process (GO:0031328); G-protein coupled receptor signaling pathway, coupled to cyclic nucleotide second messenger (GO:0007187); phospholipase C-activating G-protein coupled receptor signaling pathway (GO:0007200) | It encodes Parathyroid hormone/parathyroid hormone-related peptide receptor and whose activity stimulated by G proteins which subsequently activate adenylyl cyclase and phosphatidylinositol-calcium secondory messenger system ^1,64–66^ | YES | YES ^47^ |
| *ECM1* | Protein Coding | Underexpressed in Cancer | protease binding (GO:0002020); platelet dense granule lumen (GO:0031089); secretory granule lumen (GO:0034774); platelet degranulation (GO:0002576); positive regulation of I-kappaB kinase/NF-kappaB signaling (GO:0043123); positive regulation of intracellular signal transduction (GO:1902533) | It encodes Extracellular matrix protein 1, which Involved in endochondral bone formation. Stimulates the proliferation of endothelial cells and mediates angiogenesis. It Inhibits MMP9 proteolytic activity ^1,67–69^ | YES | YES^70,71^ |
| *GDF2* | Protein Coding | Underexpressed in Cancer | transforming growth factor beta receptor binding (GO:0005160); cytokine activity (GO:0005125); negative regulation of cell growth (GO:0030308); regulation of MAPK cascade (GO:0043408); positive regulation of protein phosphorylation (GO:0001934); positive regulation of nucleic acid-templated transcription (GO:1903508); regulation of cell growth (GO:0001558); regulation of protein phosphorylation (GO:0001932); regulation of programmed cell death (GO:0043067) | It encodes Growth/differentiation factor 2, which is a potent circulating inhibitor of angiogenesis^1,72,73^. | NO | NO |

**Table L-B. Enrichment of 53 signature genes or LCN-RNA-AUC (Cancer v/s Normal classification) in different MSigDB Oncogenic signatures.**

| **Term** | **Adjusted P-value** | **Genes** |
| --- | --- | --- |
| **35 downregulated genes** | | |
| CSR_LATE_UP.V1_UP | 0.007313 | *CXCL12;MT1F;MT1G;MT1X* |
| IL15_UP.V1_UP | 0.007313 | *ECM1;CXCL12;C9;PTH1R* |
| IL21_UP.V1_UP | 0.007313 | *ECM1;CXCL12;FOSB;PTH1R* |
| P53_DN.V2_UP | 0.034411 | *CYP2C8;CXCL12;MT1G* |
| **18 upregulated genes** | | |
| CORDENONSI_YAP_CONSERVED_SIGNATURE | 6.77E-06 | *CDC20;TOP2A;BIRC5;TK1* |
| CSR_LATE_UP.V1_UP | 6.77E-06 | *RRM2;UBE2C;GMNN;BIRC5;MYBL2* |
| RB_P130_DN.V1_UP | 0.001978 | *UBE2C;BIRC5;TK1* |
| RB_DN.V1_UP | 0.001978 | *RRM2;UBE2T;AKR1C3* |
| RB_P107_DN.V1_UP | 0.001978 | *RRM2;GMNN;E2F1* |
| MTOR_UP.V1_UP | 0.00253 | *CDC20;RRM2;UBE2C* |
| GCNP_SHH_UP_LATE.V1_UP | 0.00253 | *TOP2A;RRM2;TK1* |
| VEGF_A_UP.V1_DN | 0.00253 | *CCNB1;RRM2;BIRC5* |
| RPS14_DN.V1_DN | 0.00253 | *CDC20;MYBL2;TK1* |
| E2F1_UP.V1_UP | 0.00253 | *TOP2A;UBE2T;TK1* |
| PIGF_UP.V1_DN | 0.036024 | *CDC20;THY1* |
| ATF2_S_UP.V1_UP | 0.036024 | *TOP2A;TK1* |
| GCNP_SHH_UP_EARLY.V1_UP | 0.036024 | *TOP2A;UBE2T* |
| JNK_DN.V1_DN | 0.036024 | *UBE2C;THY1* |

**Table M.** The identified CpG sites signatures that are implicated in liver cancer and other cancers.

| Malignancy | Signatures identified for stage classification | Signatures identified for Cancer v/s normal |
| --- | --- | --- |
|  | CpG sites or their associated genes | CpG sites |
| Liver Cancer | cg16657244 associated with NOLC1 ^83^b (23970161) | 2CpG sites out of 15CpG sites (cg06353345, cg07274716) ^84,85^(22234943, 23437062) |
| Other cancers | cg07132710, cg11232136, cg18578954, cg07402003 and cg06176471 associated with *GATA2, ZNF566* , *TRIM27, TOX3,* and *ATP1B1* respectively ^86–91^ (24807155, 22284968, 16007088, 23054610, 27806084, 24452105) | PTPRN2 ^92^ (27926516), |

**Table N. The performance Random forest model in form of confusion matrix developed for classifying normal, early and late stage samples. Model was developed using 284 RNA transcripts.**

| **Training dataset** | | | | | |
| --- | --- | --- | --- | --- | --- |
| **Predicted as** | | | **Actual** | **Accuracy** | **Weighted average AUC** |
| **Late Stage** | **Early Stage** | **Normal** |
| 106 | 34 | 1 | **Late Stage** | 78.99 | 0.88 |
| 32 | 106 | 0 | **Early Stage** |
| 0 | 0 | 40 | **Normal** |
| **Independent validation dataset** | | | | | |
| **Predicted as** | | | **Actual** | **Accuracy** | **Weighted average AUC** |
|
| **Late Stage** | **Early Stage** | **Normal** |
| 24 | 12 | 0 | **Late Stage** | 70.37 | 0.8 |
| 10 | 23 | 2 | **Early Stage** |
| 0 | 0 | 10 | **Normal** |

**Table O. The Performance of prediction models using 5 & 284 RNA transcripts and 33 CpG sites for classifying normal, early and late stage samples.**

| **Technique** | **Dataset** | **TP Rate** | **FP Rate** | **Precision** | **Recall** | **F-Measure** | **MCC** | **Accuracy (%)** | **Weighted average AUC** |
| --- | --- | --- | --- | --- | --- | --- | --- | --- | --- |
| **5 RNA transcripts** | | | | | | | | | |
| **RF** | Training | 0.67 | 0.26 | 0.67 | 0.67 | 0.67 | 0.41 | 66.77 | 0.77 |
|  | validation | 0.69 | 0.24 | 0.69 | 0.69 | 0.69 | 0.45 | 69.14 | 0.81 |
| **SMO** | Training | 0.71 | 0.22 | 0.71 | 0.71 | 0.71 | 0.49 | 71.15 | 0.75 |
|  | validation | 0.68 | 0.25 | 0.68 | 0.68 | 0.68 | 0.43 | 67.90 | 0.72 |
| **Naïve bayes** | Training | 0.73 | 0.21 | 0.73 | 0.73 | 0.73 | 0.52 | 72.73 | 0.81 |
|  | validation | 0.73 | 0.21 | 0.76 | 0.73 | 0.72 | 0.54 | 72.84 | 0.80 |
| **284 RNA transcripts** | | | | | | | | | |
| **RF** | Training | 0.72 | 0.22 | 0.72 | 0.72 | 0.72 | 0.50 | 71.79 | 0.82 |
|  | validation | 0.70 | 0.23 | 0.71 | 0.70 | 0.70 | 0.48 | 70.37 | 0.82 |
| **SMO** | Training | 0.68 | 0.25 | 0.68 | 0.68 | 0.68 | 0.44 | 68.34 | 0.72 |
|  | validation | 0.68 | 0.25 | 0.68 | 0.68 | 0.68 | 0.43 | 67.90 | 0.73 |
| **Naïve bayes** | Training | 0.57 | 0.34 | 0.57 | 0.57 | 0.46 | 0.26 | 56.73 | 0.77 |
|  | validation | 0.62 | 0.31 | 0.72 | 0.62 | 0.54 | 0.39 | 61.72 | 0.79 |
| **33 CpG sites** | | | | | | | | | |
| **RF** | Training | 0.74 | 0.20 | 0.74 | 0.74 | 0.74 | 0.54 | 73.67 | 0.86 |
|  | validation | 0.74 | 0.20 | 0.75 | 0.74 | 0.74 | 0.54 | 74.07 | 0.83 |
| **SMO** | Training | 0.72 | 0.19 | 0.72 | 0.72 | 0.72 | 0.52 | 72.10 | 0.78 |
|  | validation | 0.70 | 0.22 | 0.70 | 0.70 | 0.70 | 0.48 | 70.37 | 0.75 |
| **Naïve bayes** | Training | 0.77 | 0.17 | 0.77 | 0.77 | 0.77 | 0.61 | 77.43 | 0.88 |
|  | validation | 0.77 | 0.17 | 0.77 | 0.77 | 0.76 | 0.59 | 76.54 | 0.86 |

**Table P. Functional importance of 10 signatures (LCN-RNA-AUC and LCN-5RNA) and their**

**implication in liver cancer or other malignancies.**

| **RNA transcript** | **Type of transcript** | **Regulation in late stage** | **GO terms associated with Gene** | **Function** | **Reported in Previous Literature** | **Reported in case of**  **Liver cancer** |
| --- | --- | --- | --- | --- | --- | --- |
| *C7* | Protein Coding | Underexpressed in Cancer | regulation of immune effector process (GO:0002697); regulation of humoral immune response (GO:0002920); regulation of protein activation cascade (GO:2000257); regulation of protein processing (GO:0070613); membrane attack complex (GO:0005579); integral component of plasma membrane (GO:0005887) | It encodes complement component C7, a constituent of the membrane attack complex (MAC) that plays a key role in the innate and adaptive immune response by forming pores in the plasma membrane of target cells. It also acts as a membrane anchor ^1^. | YES^74^ | YES^75^ |
| *GMNN* | Protein Coding | Underexpressed in Cancer | positive regulation of chromatin binding (GO:0035563); regulation of chromatin binding (GO:0035561); negative regulation of DNA replication (GO:0008156); negative regulation of DNA-dependent DNA replication (GO:2000104);  negative regulation of cell cycle (GO:0045786) | It encodes Geminin, Inhibits DNA replication by preventing the incorporation of MCM complex into pre-replication complex (pre-RC). It is degraded during the mitotic phase of the cell cycle. Its destruction at the metaphase-anaphase transition permits replication in the succeeding cell cycle.  Inhibits the transcriptional activity of a subset of Hox proteins, enrolling them in cell proliferative control^1^ | YES | YES^47,76^ |
| *EEF1A2* | Protein Coding | Overexpressed in Cancer | regulation of chaperone-mediated autophagy (GO:1904714); regulation of lipid kinase activity (GO:0043550); positive regulation of lipid metabolic process (GO:0045834); regulation of cellular amide metabolic process (GO:0034248); positive regulation of kinase activity (GO:0033674); GTPase activity (GO:0003924); lysosomal membrane (GO:0005765) | It encodes Elongation factor 1-alpha 2, which promotes the GTP-dependent binding of aminoacyl-tRNA to the A-site of ribosomes during protein biosynthesis ^1^. | YES | YES^77^ |
| *MT1E* | Protein Coding | Underexpressed in Cancer | cellular divalent inorganic cation homeostasis (GO:0072503); negative regulation of growth (GO:0045926); cellular response to zinc ion (GO:0071294); response to copper ion (GO:0046688); cellular response to cadmium ion (GO:0071276); perinuclear region of cytoplasm (GO:0048471) | It encodes Metallothionein-1E, Metallothioneins are rich in cysteine residues which bind to various heavy metals. They are transcriptionally regulated by both heavy metals and glucocorticoids ^1^. | YES^78,79^ | NO |
| *CNDP1* | Protein Coding | Underexpressed in Cancer | regulation of protein metabolic process (GO:0051246); regulation of cellular metabolic process (GO:0031323); proteolysis (GO:0006508); dipeptidase activity (GO:0016805); exopeptidase activity (GO:0008238) | It encodes Beta-Ala-His dipeptidase, which hydrolyze the carnosine (beta-Ala-|-His dipeptide), anserine (Xaa-|-His) dipeptides and other dipeptides including homocarnosine ^80,81^. | YES^82^ | NO |

**Table Q. The Performance of prediction models using 51 features & 25 features ( containing both RNA transcripts and CpG sites) for classifying stage-I, stage-II and stage-III-IV samples.**

| **Technique** | **Dataset** | **TP Rate** | **FP Rate** | **Precision** | **Recall** | **F-Measure** | **MCC** | **Accuracy (%)** | **Weighted average AUC** |
| --- | --- | --- | --- | --- | --- | --- | --- | --- | --- |
| **51 features** | | | | | | | | | |
| **RF** | Training | 0.59 | 0.26 | 0.55 | 0.59 | 0.56 | 0.34 | 59.14 | 0.74 |
|  | validation | 0.65 | 0.21 | 0.63 | 0.65 | 0.63 | 0.45 | 64.79 | 0.76 |
| **SMO** | Training | 0.53 | 0.43 | 0.56 | 0.53 | 0.41 | 0.20 | 53.41 | 0.71 |
|  | validation | 0.52 | 0.43 | 0.51 | 0.52 | 0.42 | 0.48 | 52.12 | 0.66 |
| **Naïve bayes** | Training | 0.59 | 0.25 | 0.54 | 0.59 | 0.56 | 0.36 | 59.13 | 0.79 |
|  | validation | 0.66 | 0.21 | 0.64 | 0.66 | 0.64 | 0.47 | 66.19 | 0.84 |
| **25 features** | | | | | | | | | |
| **RF** | Training | 0.62 | 0.25 | 0.61 | 0.62 | 0.61 | 0.4 | 63.63 | 0.8 |
|  | validation | 0.56 | 0.26 | 0.51 | 0.55 | 0.56 | 0.31 | 56.34 | 0.75 |
| **SMO** | Training | 0.53 | 0.43 | 0.56 | 0.53 | 0.41 | 0.20 | 53.41 | 0.71 |
|  | validation | 0.52 | 0.43 | 0.51 | 0.52 | 0.42 | 0.15 | 52.11 | 0.66 |
| **Naïve bayes** | Training | 0.61 | 0.27 | 0.59 | 0.61 | 0.59 | 0.37 | 61.29 | 0.79 |
|  | validation | 0.54 | 0.29 | 0.49 | 0.54 | 0.5 | 0.25 | 53.52 | 0.72 |

**Table R. The Performance of prediction models using 51 features & 25 features ( containing both RNA transcripts and CpG sites) for classifying stage-I, stage-II and stage-III samples (removing stage-IV samples).**

| **Technique** | **Dataset** | **TP Rate** | **FP Rate** | **Precision** | **Recall** | **F-Measure** | **MCC** | **Accuracy (%)** | **Weighted average AUC** |
| --- | --- | --- | --- | --- | --- | --- | --- | --- | --- |
| **51 features** | | | | | | | | | |
| **RF** | Training | 0.59 | 0.26 | 0.55 | 0.59 | 0.56 | 0.34 | 59.14 | 0.74 |
|  | validation | 0.65 | 0.21 | 0.63 | 0.65 | 0.63 | 0.45 | 64.79 | 0.76 |
| **SMO** | Training | 0.56 | 0.34 | 0.51 | 0.56 | 0.5 | 0.26 | 56 | 0.70 |
|  | validation | 0.67 | 0.22 | 0.65 | 0.67 | 0.65 | 0.48 | 67.14 | 0.76 |
| **Naïve bayes** | Training | 0.63 | 0.25 | 0.59 | 0.63 | 0.60 | 0.4 | 62.55 | 0.80 |
|  | validation | 0.64 | 0.22 | 0.62 | 0.64 | 0.62 | 0.44 | 64.29 | 0.83 |
| **25 features** | | | | | | | | | |
| **RF** | Training | 0.64 | 0.25 | 0.62 | 0.64 | 0.61 | 0.41 | 63.63 | 0.77 |
|  | validation | 0.54 | 0.32 | 0.51 | 0.54 | 0.51 | 0.24 | 54.29 | 0.7 |
| **SMO** | Training | 0.56 | 0.41 | 0.6 | 0.56 | 0.47 | 0.26 | 56.36 | 0.69 |
|  | validation | 0.56 | 0.37 | 0.5 | 0.56 | 0.48 | 0.23 | 55.71 | 0.67 |
| **Naïve bayes** | Training | 0.62 | 0.27 | 0.6 | 0.62 | 0.6 | 0.38 | 62.18 | 0.78 |
|  | validation | 0.54 | 0.29 | 0.5 | 0.54 | 0.51 | 0.27 | 54.28 | 0.72 |

**References**

1. UniProt Consortium, T. UniProt: the universal protein knowledgebase. *Nucleic Acids Res.* **46,** 2699 (2018).

2. Yin, L. *et al.* NCAPH plays important roles in human colon cancer. *Cell Death Dis.* **8,** e2680 (2017).

3. Zhan, S.-J., Liu, B. & Linghu, H. Identifying genes as potential prognostic indicators in patients with serous ovarian cancer resistant to carboplatin using integrated bioinformatics analysis. *Oncol. Rep.* **39,** 2653–2663 (2018).

4. Pikuleva, I. A. & Waterman, M. R. Cytochromes p450: roles in diseases. *J. Biol. Chem.* **288,** 17091–8 (2013).

5. Kawashima, H. *et al.* Human fatty acid omega-hydroxylase, CYP4A11: determination of complete genomic sequence and characterization of purified recombinant protein. *Arch. Biochem. Biophys.* **378,** 333–9 (2000).

6. Kimura, K., Cuvier, O. & Hirano, T. Chromosome condensation by a human condensin complex in Xenopus egg extracts. *J. Biol. Chem.* **276,** 5417–20 (2001).

7. Chetyrkin, S. V, Hu, J., Gough, W. H., Dumaual, N. & Kedishvili, N. Y. Further characterization of human microsomal 3alpha-hydroxysteroid dehydrogenase. *Arch. Biochem. Biophys.* **386,** 1–10 (2001).

8. Huang, X. F. & Luu-The, V. Molecular characterization of a first human 3(alpha--&gt;beta)-hydroxysteroid epimerase. *J. Biol. Chem.* **275,** 29452–7 (2000).

9. Yao, F., Zhang, C., Du, W., Liu, C. & Xu, Y. Identification of Gene-Expression Signatures and Protein Markers for Breast Cancer Grading and Staging. *PLoS One* **10,** e0138213 (2015).

10. Matsuo, M. *et al.* Designation of enzyme activity of glycine-N-acyltransferase family genes and depression of glycine-N-acyltransferase in human hepatocellular carcinoma. *Biochem. Biophys. Res. Commun.* **420,** 901–6 (2012).

11. Seimiya, M. *et al.* Identification of novel immunohistochemical tumor markers for primary hepatocellular carcinoma; clathrin heavy chain and formiminotransferase cyclodeaminase. *Hepatology* **48,** 519–30 (2008).

12. Gimm, T. *et al.* Hypoxia-inducible protein 2 is a novel lipid droplet protein and a specific target gene of hypoxia-inducible factor-1. *FASEB J.* **24,** 4443–58 (2010).

13. Togashi, A. *et al.* Hypoxia-inducible protein 2 (HIG2), a novel diagnostic marker for renal cell carcinoma and potential target for molecular therapy. *Cancer Res.* **65,** 4817–26 (2005).

14. Shah, M. B. *et al.* Conformational adaptation of human cytochrome P450 2B6 and rabbit cytochrome P450 2B4 revealed upon binding multiple amlodipine molecules. *Biochemistry* **51,** 7225–38 (2012).

15. Gay, S. C. *et al.* Crystal structure of a cytochrome P450 2B6 genetic variant in complex with the inhibitor 4-(4-chlorophenyl)imidazole at 2.0-A resolution. *Mol. Pharmacol.* **77,** 529–38 (2010).

16. Shah, M. B., Pascual, J., Zhang, Q., Stout, C. D. & Halpert, J. R. Structures of cytochrome P450 2B6 bound to 4-benzylpyridine and 4-(4-nitrobenzyl)pyridine: insight into inhibitor binding and rearrangement of active site side chains. *Mol. Pharmacol.* **80,** 1047–55 (2011).

17. Esposti, D. D. *et al.* Identification of novel long non-coding RNAs deregulated in hepatocellular carcinoma using RNA-sequencing. *Oncotarget* **7,** 31862–77 (2016).

18. Dai, M. *et al.* Diagnosis, prognosis and bioinformatics analysis of lncRNAs in hepatocellular carcinoma. *Oncotarget* **8,** 95799–95809 (2017).

19. Nizon, M. *et al.* Further delineation of CANT1 phenotypic spectrum and demonstration of its role in proteoglycan synthesis. *Hum. Mutat.* **33,** 1261–6 (2012).

20. Smith, T. M., Hicks-Berger, C. A., Kim, S. & Kirley, T. L. Cloning, expression, and characterization of a soluble calcium-activated nucleotidase, a human enzyme belonging to a new family of extracellular nucleotidases. *Arch. Biochem. Biophys.* **406,** 105–15 (2002).

21. Yang, M. & Kirley, T. L. Site-directed mutagenesis of human soluble calcium-activated nucleotidase 1 (hSCAN-1): identification of residues essential for enzyme activity and the Ca(2+)-induced conformational change. *Biochemistry* **43,** 9185–94 (2004).

22. Xing, Y. *et al.* CANT1 lncRNA Triggers Efficient Therapeutic Efficacy by Correcting Aberrant lncing Cascade in Malignant Uveal Melanoma. *Mol. Ther.* **25,** 1209–1221 (2017).

23. Zhang, Z.-G., Chen, W.-X., Wu, Y.-H., Liang, H.-F. & Zhang, B.-X. MiR-132 prohibits proliferation, invasion, migration, and metastasis in breast cancer by targeting HN1. *Biochem. Biophys. Res. Commun.* **454,** 109–14 (2014).

24. Varisli, L., Ozturk, B. E., Akyuz, G. K. & Korkmaz, K. S. HN1 negatively influences the β-catenin/E-cadherin interaction, and contributes to migration in prostate cells. *J. Cell. Biochem.* **116,** 170–8 (2015).

25. Hamaguchi, T. *et al.* Glycolysis module activated by hypoxia-inducible factor 1alpha is related to the aggressive phenotype of hepatocellular carcinoma. *Int. J. Oncol.* **33,** 725–31 (2008).

26. Chaudhary, K., Poirion, O. B., Lu, L. & Garmire, L. X. Deep Learning-Based Multi-Omics Integration Robustly Predicts Survival in Liver Cancer. *Clin. Cancer Res.* **24,** 1248–1259 (2018).

27. Michibata, H. *et al.* Identification and characterization of a novel component of the cornified envelope, cornifelin. *Biochem. Biophys. Res. Commun.* **318,** 803–13 (2004).

28. Kim, Y. H. *et al.* SLC2A2 (GLUT2) as a novel prognostic factor for hepatocellular carcinoma. *Oncotarget* **8,** 68381–68392 (2017).

29. Hamm, A. *et al.* Frequent expression loss of Inter-alpha-trypsin inhibitor heavy chain (ITIH) genes in multiple human solid tumors: a systematic expression analysis. *BMC Cancer* **8,** 25 (2008).

30. Canales, N. A. G. *et al.* A1BG and C3 are overexpressed in patients with cervical intraepithelial neoplasia III. *Oncol. Lett.* **8,** 939–947 (2014).

31. Gómez-Maldonado, L. *et al.* EFNA3 long noncoding RNAs induced by hypoxia promote metastatic dissemination. *Oncogene* **34,** 2609–20 (2015).

32. Li, H.-J. *et al.* Identification of metabolism-associated genes and pathways involved in different stages of clear cell renal cell carcinoma. *Oncol. Lett.* **15,** 2316–2322 (2018).

33. Torres, L. *et al.* Liver-specific methionine adenosyltransferase MAT1A gene expression is associated with a specific pattern of promoter methylation and histone acetylation: implications for MAT1A silencing during transformation. *FASEB J.* **14,** 95–102 (2000).

34. Mato, J. M., Alvarez, L., Ortiz, P. & Pajares, M. A. S-adenosylmethionine synthesis: molecular mechanisms and clinical implications. *Pharmacol. Ther.* **73,** 265–80 (1997).

35. Lu, S. C. & Mato, J. M. Role of methionine adenosyltransferase and S-adenosylmethionine in alcohol-associated liver cancer. *Alcohol* **35,** 227–34 (2005).

36. Sabini, E., Hazra, S., Konrad, M. & Lavie, A. Elucidation of different binding modes of purine nucleosides to human deoxycytidine kinase. *J. Med. Chem.* **51,** 4219–25 (2008).

37. Staub, M. [Special function of deoxycytidine kinase (dCK) in the activation of chemotherapeutic nucleoside analogs and in the inhibition of cell proliferation]. *Magy. Onkol.* **48,** 229–34 (2004).

38. Woo, S. M. *et al.* DCK expression, a potential predictive biomarker in the adjuvant gemcitabine chemotherapy for biliary tract cancer after surgical resection: results from a phase II study. *Oncotarget* **8,** 81394–81404 (2017).

39. Schmitz, J., Watrin, E., Lénárt, P., Mechtler, K. & Peters, J.-M. Sororin is required for stable binding of cohesin to chromatin and for sister chromatid cohesion in interphase. *Curr. Biol.* **17,** 630–6 (2007).

40. Rankin, S., Ayad, N. G. & Kirschner, M. W. Sororin, a substrate of the anaphase-promoting complex, is required for sister chromatid cohesion in vertebrates. *Mol. Cell* **18,** 185–200 (2005).

41. Shen, Z. *et al.* CDCA5 regulates proliferation in hepatocellular carcinoma and has potential as a negative prognostic marker. *Onco. Targets. Ther.* **11,** 891–901 (2018).

42. Yang, H.-J., Vainshtein, A., Maik-Rachline, G. & Peles, E. G protein-coupled receptor 37 is a negative regulator of oligodendrocyte differentiation and myelination. *Nat. Commun.* **7,** 10884 (2016).

43. Meyer, R. C., Giddens, M. M., Schaefer, S. A. & Hall, R. A. GPR37 and GPR37L1 are receptors for the neuroprotective and glioprotective factors prosaptide and prosaposin. *Proc. Natl. Acad. Sci. U. S. A.* **110,** 9529–34 (2013).

44. Kosek, A. B., Durbin, D. & Jonas, A. Binding affinity and reactivity of lecithin cholesterol acyltransferase with native lipoproteins. *Biochem. Biophys. Res. Commun.* **258,** 548–51 (1999).

45. Hirsch-Reinshagen, V. *et al.* LCAT synthesized by primary astrocytes esterifies cholesterol on glia-derived lipoproteins. *J. Lipid Res.* **50,** 885–93 (2009).

46. Piper, D. E. *et al.* The high-resolution crystal structure of human LCAT. *J. Lipid Res.* **56,** 1711–9 (2015).

47. Yin, F. *et al.* Microarray-based identification of genes associated with cancer progression and prognosis in hepatocellular carcinoma. *J. Exp. Clin. Cancer Res.* **35,** 127 (2016).

48. Lenhart, P. M., Broselid, S., Barrick, C. J., Leeb-Lundberg, L. M. F. & Caron, K. M. G-protein-coupled receptor 30 interacts with receptor activity-modifying protein 3 and confers sex-dependent cardioprotection. *J. Mol. Endocrinol.* **51,** 191–202 (2013).

49. Barrick, C. J., Lenhart, P. M., Dackor, R. T., Nagle, E. & Caron, K. M. Loss of receptor activity-modifying protein 3 exacerbates cardiac hypertrophy and transition to heart failure in a sex-dependent manner. *J. Mol. Cell. Cardiol.* **52,** 165–74 (2012).

50. Szklarczyk, D. *et al.* STRING v10: protein-protein interaction networks, integrated over the tree of life. *Nucleic Acids Res.* **43,** D447-52 (2015).

51. Ha, M. J., Baladandayuthapani, V. & Do, K.-A. Prognostic gene signature identification using causal structure learning: applications in kidney cancer. *Cancer Inform.* **14,** 23–35 (2015).

52. Hu, L. *et al.* Upregulation of NETO2 expression correlates with tumor progression and poor prognosis in colorectal carcinoma. *BMC Cancer* **15,** 1006 (2015).

53. Liu, W. *et al.* Characterization of a novel C-type lectin-like gene, LSECtin: demonstration of carbohydrate binding and expression in sinusoidal endothelial cells of liver and lymph node. *J. Biol. Chem.* **279,** 18748–58 (2004).

54. Shimojima, M., Takenouchi, A., Shimoda, H., Kimura, N. & Maeda, K. Distinct usage of three C-type lectins by Japanese encephalitis virus: DC-SIGN, DC-SIGNR, and LSECtin. *Arch. Virol.* **159,** 2023–31 (2014).

55. Gramberg, T. *et al.* LSECtin interacts with filovirus glycoproteins and the spike protein of SARS coronavirus. *Virology* **340,** 224–36 (2005).

56. Ho, D. W.-H., Kai, A. K.-L. & Ng, I. O.-L. TCGA whole-transcriptome sequencing data reveals significantly dysregulated genes and signaling pathways in hepatocellular carcinoma. *Front. Med.* **9,** 322–30 (2015).

57. Stan, R. V, Tkachenko, E. & Niesman, I. R. PV1 is a key structural component for the formation of the stomatal and fenestral diaphragms. *Mol. Biol. Cell* **15,** 3615–30 (2004).

58. Wang, Y.-H. *et al.* Plasmalemmal Vesicle Associated Protein (PLVAP) as a therapeutic target for treatment of hepatocellular carcinoma. *BMC Cancer* **14,** 815 (2014).

59. Munye, M. M. *et al.* COLEC10 is mutated in 3MC patients and regulates early craniofacial development. *PLoS Genet.* **13,** e1006679 (2017).

60. Ohtani, K. *et al.* Molecular cloning of a novel human collectin from liver (CL-L1). *J. Biol. Chem.* **274,** 13681–9 (1999).

61. Zhang, B. & Wu, H. Decreased expression of COLEC10 predicts poor overall survival in patients with hepatocellular carcinoma. *Cancer Manag. Res.* **10,** 2369–2375 (2018).

62. Roy, D. R. Effect of magnesium loading on magnesium delivery to the juxtamedullary end-descending limb. *Am. J. Physiol.* **248,** F145-51 (1985).

63. Lv, Y. *et al.* Overexpression of NDUFA4L2 is associated with poor prognosis in patients with colorectal cancer. *ANZ J. Surg.* **87,** E251–E255 (2017).

64. Pioszak, A. A., Harikumar, K. G., Parker, N. R., Miller, L. J. & Xu, H. E. Dimeric arrangement of the parathyroid hormone receptor and a structural mechanism for ligand-induced dissociation. *J. Biol. Chem.* **285,** 12435–44 (2010).

65. Shimomura-Kuroki, J., Farooq, M., Sekimoto, T., Amizuka, N. & Shimomura, Y. Characterization of a PTH1R missense mutation responsible for Jansen type metaphyseal chondrodysplasia. *Odontology* **105,** 150–154 (2017).

66. Schneider, H., Feyen, J. H., Seuwen, K. & Movva, N. R. Cloning and functional expression of a human parathyroid hormone receptor. *Eur. J. Pharmacol.* **246,** 149–55 (1993).

67. Deckers, M. M. *et al.* Recombinant human extracellular matrix protein 1 inhibits alkaline phosphatase activity and mineralization of mouse embryonic metatarsals in vitro. *Bone* **28,** 14–20 (2001).

68. Han, Z. *et al.* Extracellular matrix protein 1 (ECM1) has angiogenic properties and is expressed by breast tumor cells. *FASEB J.* **15,** 988–94 (2001).

69. Fujimoto, N. *et al.* Extracellular matrix protein 1 inhibits the activity of matrix metalloproteinase 9 through high-affinity protein/protein interactions. *Exp. Dermatol.* **15,** 300–7 (2006).

70. Chen, H. *et al.* Extracellular matrix protein 1, a novel prognostic factor, is associated with metastatic potential of hepatocellular carcinoma. *Med. Oncol.* **28 Suppl 1,** S318-25 (2011).

71. Chen, H., Jia, W. & Li, J. ECM1 promotes migration and invasion of hepatocellular carcinoma by inducing epithelial-mesenchymal transition. *World J. Surg. Oncol.* **14,** 195 (2016).

72. David, L. *et al.* Bone morphogenetic protein-9 is a circulating vascular quiescence factor. *Circ. Res.* **102,** 914–22 (2008).

73. Wei, Z., Salmon, R. M., Upton, P. D., Morrell, N. W. & Li, W. Regulation of bone morphogenetic protein 9 (BMP9) by redox-dependent proteolysis. *J. Biol. Chem.* **289,** 31150–9 (2014).

74. Ying, L. *et al.* Complement component 7 (C7), a potential tumor suppressor, is correlated with tumor progression and prognosis. *Oncotarget* **7,** 86536–86546 (2016).

75. Seol, H. S. *et al.* Complement proteins C7 and CFH control the stemness of liver cancer cells via LSF-1. *Cancer Lett.* **372,** 24–35 (2016).

76. Kim, H.-E. *et al.* Frequent amplification of CENPF, GMNN and CDK13 genes in hepatocellular carcinomas. *PLoS One* **7,** e43223 (2012).

77. Pellegrino, R. *et al.* EEF1A2 inactivates p53 by way of PI3K/AKT/mTOR-dependent stabilization of MDM4 in hepatocellular carcinoma. *Hepatology* **59,** 1886–99 (2014).

78. Demidenko, R. *et al.* Decreased expression of MT1E is a potential biomarker of prostate cancer progression. *Oncotarget* **8,** 61709–61718 (2017).

79. Wu, Y., Siadaty, M. S., Berens, M. E., Hampton, G. M. & Theodorescu, D. Overlapping gene expression profiles of cell migration and tumor invasion in human bladder cancer identify metallothionein 1E and nicotinamide N-methyltransferase as novel regulators of cell migration. *Oncogene* **27,** 6679–89 (2008).

80. Lenney, J. F. *et al.* Human serum carnosinase: characterization, distinction from cellular carnosinase, and activation by cadmium. *Clin. Chim. Acta.* **123,** 221–31 (1982).

81. Teufel, M. *et al.* Sequence identification and characterization of human carnosinase and a closely related non-specific dipeptidase. *J. Biol. Chem.* **278,** 6521–31 (2003).

82. Arner, P. *et al.* Circulating Carnosine Dipeptidase 1 Associates with Weight Loss and Poor Prognosis in Gastrointestinal Cancer. *PLoS One* **10,** e0123566 (2015).

83. Duan, X. *et al.* Methylation of nucleolar and coiled-body phosphoprotein 1 is associated with the mechanism of tumorigenesis in hepatocellular carcinoma. *Oncol. Rep.* **30,** 2220–8 (2013).

84. Shen, J. *et al.* Genome-wide DNA methylation profiles in hepatocellular carcinoma. *Hepatology* **55,** 1799–808 (2012).

85. Song, M.-A. *et al.* Elucidating the landscape of aberrant DNA methylation in hepatocellular carcinoma. *PLoS One* **8,** e55761 (2013).

86. Weber, M. *et al.* Chromosome-wide and promoter-specific analyses identify sites of differential DNA methylation in normal and transformed human cells. *Nat. Genet.* **37,** 853–62 (2005).

87. Heyn, H. *et al.* DNA methylation profiling in breast cancer discordant identical twins identifies DOK7 as novel epigenetic biomarker. *Carcinogenesis* **34,** 102–8 (2013).

88. Kandimalla, R. *et al.* Genome-wide analysis of CpG island methylation in bladder cancer identified TBX2, TBX3, GATA2, and ZIC4 as pTa-specific prognostic markers. *Eur. Urol.* **61,** 1245–56 (2012).

89. Selvakumar, P. *et al.* Epigenetic silencing of Na,K-ATPase β 1 subunit gene ATP1B1 by methylation in clear cell renal cell carcinoma. *Epigenetics* **9,** 579–86 (2014).

90. Tessema, M. *et al.* GATA2 is epigenetically repressed in human and mouse lung tumors and is not requisite for survival of KRAS mutant lung cancer. *J. Thorac. Oncol.* **9,** 784–93 (2014).

91. Han, Y.-J., Zhang, J., Zheng, Y., Huo, D. & Olopade, O. I. Genetic and Epigenetic Regulation of TOX3 Expression in Breast Cancer. *PLoS One* **11,** e0165559 (2016).

92. Kajiura, K. *et al.* Frequent silencing of the candidate tumor suppressor TRIM58 by promoter methylation in early-stage lung adenocarcinoma. *Oncotarget* **8,** 2890–2905 (2017).
