# Supplementary material for: Classification of early and late stage liver hepatocellular carcinoma patients from their genomics and epigenomics profiles
Source: PLoS One. 2019 Sep 6;14(9):e0221476. doi: 10.1371/journal.pone.0221476 (PMC6730898; doi:10.1371/journal.pone.0221476)
Supplement: S2 File — (DOCX) [file pone.0221476.s002.docx]

**Supplementary Information**

**Classification of early and late stage Liver Hepatocellular Carcinoma patients from their genomics and epigenomics profiles.**

Harpreet Kaur^1^, Sherry Bhalla^2,3^, Gajendra P.S. Raghava^2*^

1. Bioinformatics Centre, CSIR-Institute of Microbial Technology, Sector 39A, Chandigarh-160036, India
2. Department of Computational Biology, Indraprastha Institute of Information Technology, New Delhi, India.
3. Centre for Systems Biology and Bioinformatics, Panjab University, Sector 14, Chandigarh-160014, India

Figure A. Clinical characteristics of LIHC patients used in current study.

**
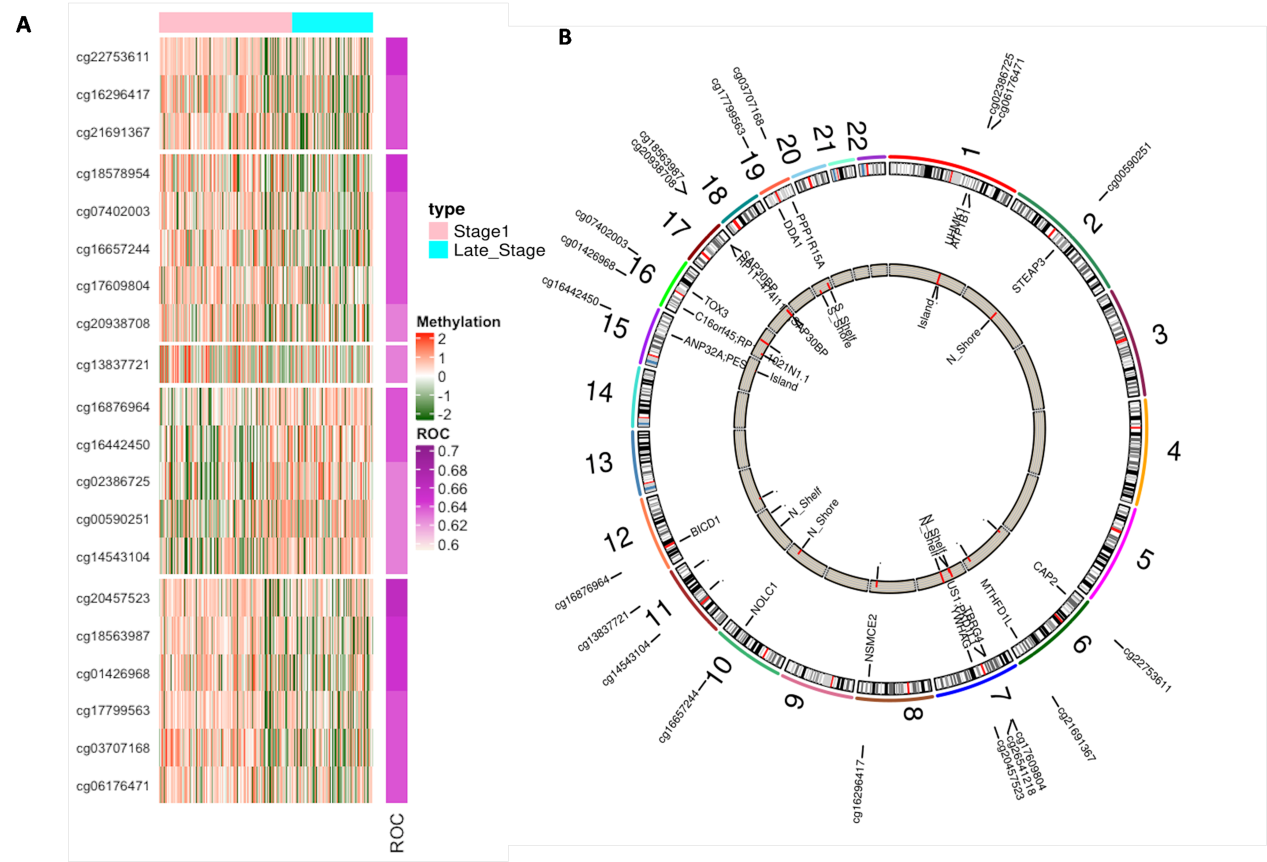
**

Figure B. A) Heatmap displaying the differential methylation pattern (with FDR < 0.05) and B) Circos plot representing the chromosome location of top 20 CpG sites (LS-CPG-AUROC) in early versus late stage of LIHC.

**
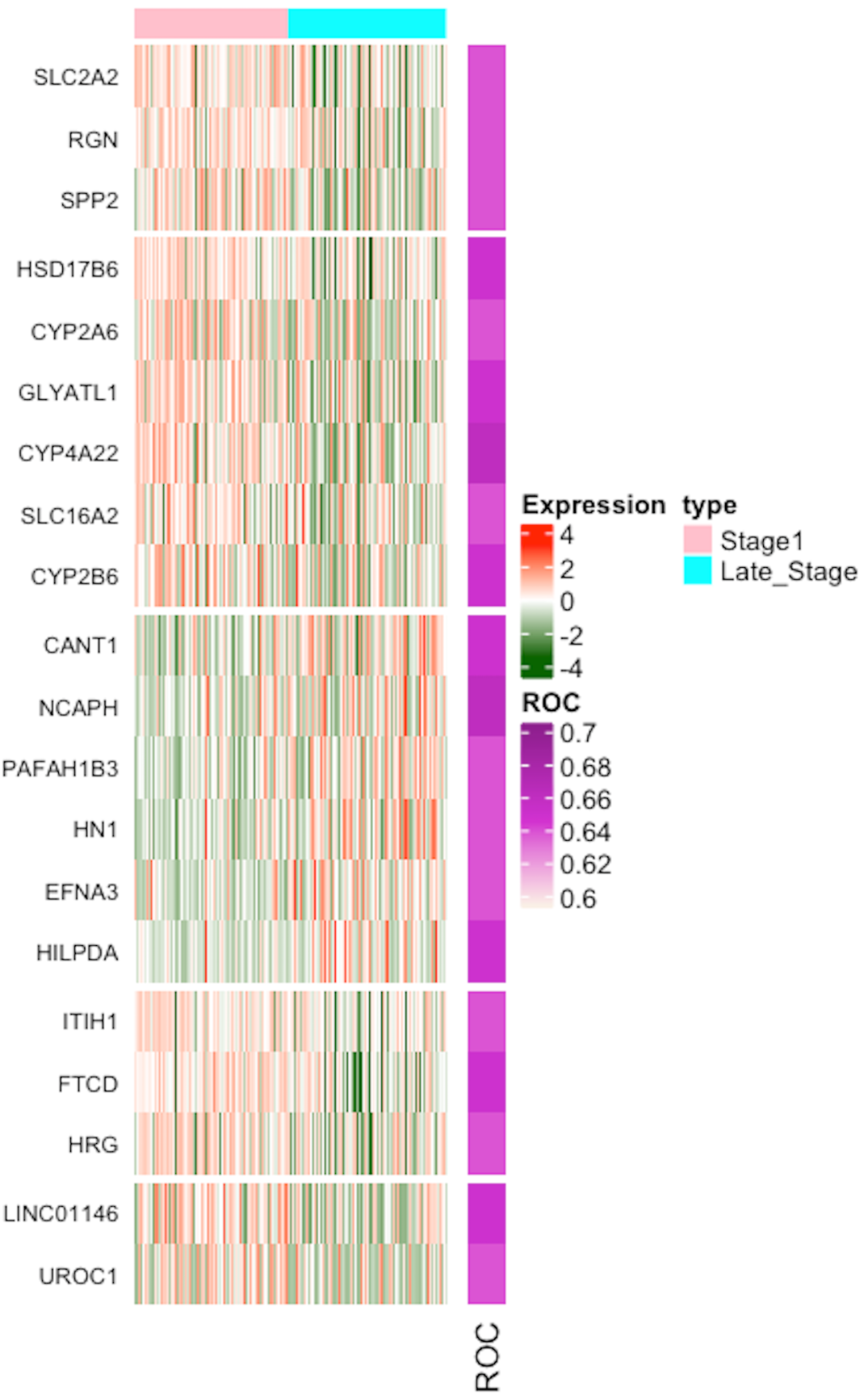
**

Figure C. Heatmap displaying the differential methylation pattern of top 20 RNA transcripts (LS-RNA-AUROC) in early versus late stage of LIHC.


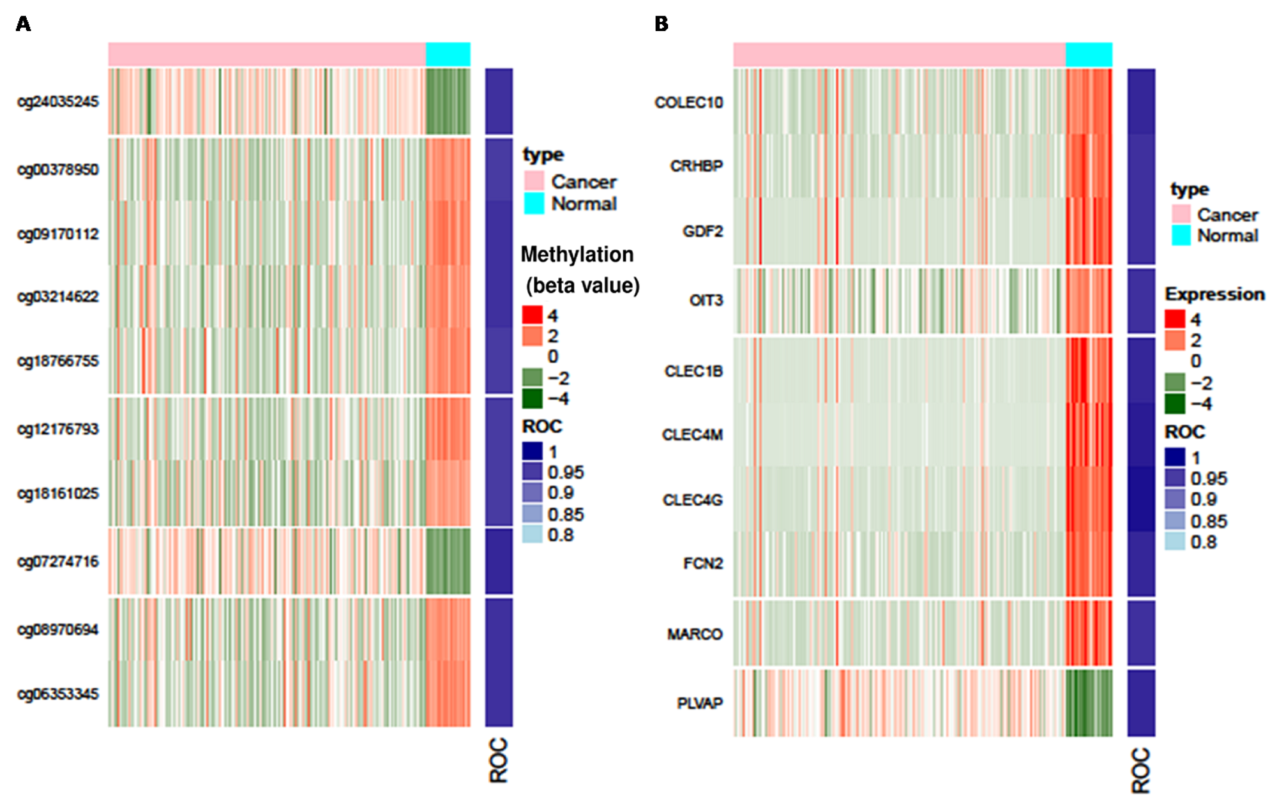


Figure D. Heatmap representing the A) differential methylation pattern (with Bonferroni *p-value* <0.001) of top 10 CpG sites (LCN-CPG-AUROC); and B) differential expression pattern (with Bonferroni *p-value* <0.001) of top 10 RNA transcripts (LCN-RNA-AUROC) in LIHC versus normal tissue samples.

Figure E. Cicos plot representing the chromosome locations and associated genes of top 10CpG sites in LIHC versus normal tissue samples.

Figure F. (A) Gene Enrichment analysis of LCN-RNA-AUROC: 18 upregulated RNA transcripts in LIHC and normal tissue samples.

Figure F. (B) Gene Enrichment analysis of LCN-RNA-AUROC: 35 downregulated RNA transcripts in LIHC and normal tissue samples.

A

B

Figure G. Boxplots represents (A) the methylation pattern of multiclass-CpG sites (33 CpG sites); and (B) the expression pattern of multiclass-RNA transcripts (5 RNA transcripts) in normal, stage-1 (early stage) and late stage tissue samples of LIHC.

A

B

Figure H. (A) Gene Enrichment analysis of multiclass-RNA, 2 upregulated RNA transcripts; and (B) 3 downregulated RNA transcripts with progression of cancer.
